# Helically Arranged Chiral Molecular Nanographenes

**DOI:** 10.1021/jacs.1c05977

**Published:** 2021-07-20

**Authors:** Patricia Izquierdo-García, Jesús M. Fernández-García, Israel Fernández, Josefina Perles, Nazario Martín

**Affiliations:** † Departamento de Química Orgánica I, Facultad de Ciencias Químicas, Universidad Complutense, 28040 Madrid, Spain; ‡ IMDEA-Nanociencia, C/Faraday, 9, Campus de Cantoblanco, 28049 Madrid, Spain; # Single Crystal X-ray Diffraction Laboratory, Interdepartmental Research Service (SIdI), Universidad Autónoma, Campus de Cantoblanco, 28049 Madrid, Spain

## Abstract

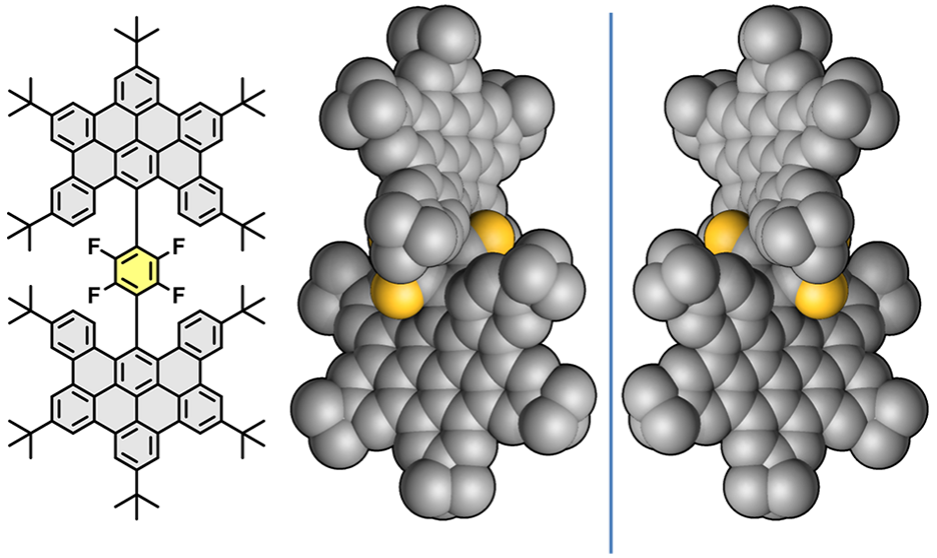

A benchtop solution-phase synthesis of molecular nanographenes composed of two orthogonal dibenzo[fg,ij]phenanthro[9,10,1,2,3-pqrst]pentaphene (DBPP) moieties covalently connected through a tetrafluorobenzene ring is described. The helical arrangement of these three covalently linked molecular fragments leads to the existence of a chiral axis which gives rise to a racemic mixture, even with the molecular moieties being symmetrically substituted. X-ray diffraction studies show that both enantiomers cocrystallize in a single crystal, and the racemic mixture can be resolved by chiral HPLC. Asymmetric substitution in DBPP moieties affords a pair of diastereoisomers whose rotational isomerization has been studied by ^1^H NMR. Additionally, the electrochemical and photophysical properties derived from these new molecular nanographenes reveal an electroactive character and a significant fluorescent behavior.

## Introduction

The groundbreaking discovery of 2D graphene as a new nanoform of carbon by A. Geim and K. Novoselov in 2004 had an extraordinary impact in the field of materials science.^1^ Actually, it paved the way for the development of unprecedented monolayer materials involving other chemical elements of the periodic table, thus starting the emergent age of 2D materials.^2^ Despite the outstanding chemical and physical properties of pristine graphene, the zero bandgap between its conduction (CB) and valence (VB) bands has limited its use in the search for optoelectronic properties and applications such as field-effect transistors,^3^ sensing,^4^ or photovoltaics,^5^ just to name a few.

In contrast to pristine graphene, the quantum confinement of electrons in smaller C(sp^2^) lattice structures, the so-called carbon nanoribbons and nanographenes (NGs) or graphene quantum dots (GQDs), increases the HOMO–LUMO gap, thus broadening the range of potential applications.^6^

Initially, carbon flakes without size control were formed from graphene by using an oxidative top-down approach.^7^ More recently, bottom-up synthesis of molecular nanographenes by accessing the realm of modern organic reactions allows the precise control of morphology and size and, therefore, the fine-tuning of electronic properties at will.^8^ In this way, this bottom-up approach has led to the preparation of NGs with a wide variety of shapes, namely planar nanographenes,^9^ bilayers,^10^ bowls,^11^ saddles,^12^ helical nanographenes,^13^ nanoribbons,^14^ nanobelts,^15^ and propellers.^16^ In the past few years, the introduction of chiral elements in nanographenes synthesized by the bottom-up approach and the study of their chiroptical properties have shown a special relevance.^17^

Chirality in molecular nanographenes is a consequence of morphological defects in the hexagonal honeycomb structure, namely stemming from (i) the presence of helicene moieties and/or (ii) the presence of nonhexagonal rings, which leads to either positive Gaussian curvature (five-membered or smaller rings) and/or negative Gaussian curvature (seven-membered or larger rings).^18^

**Chart 1:**
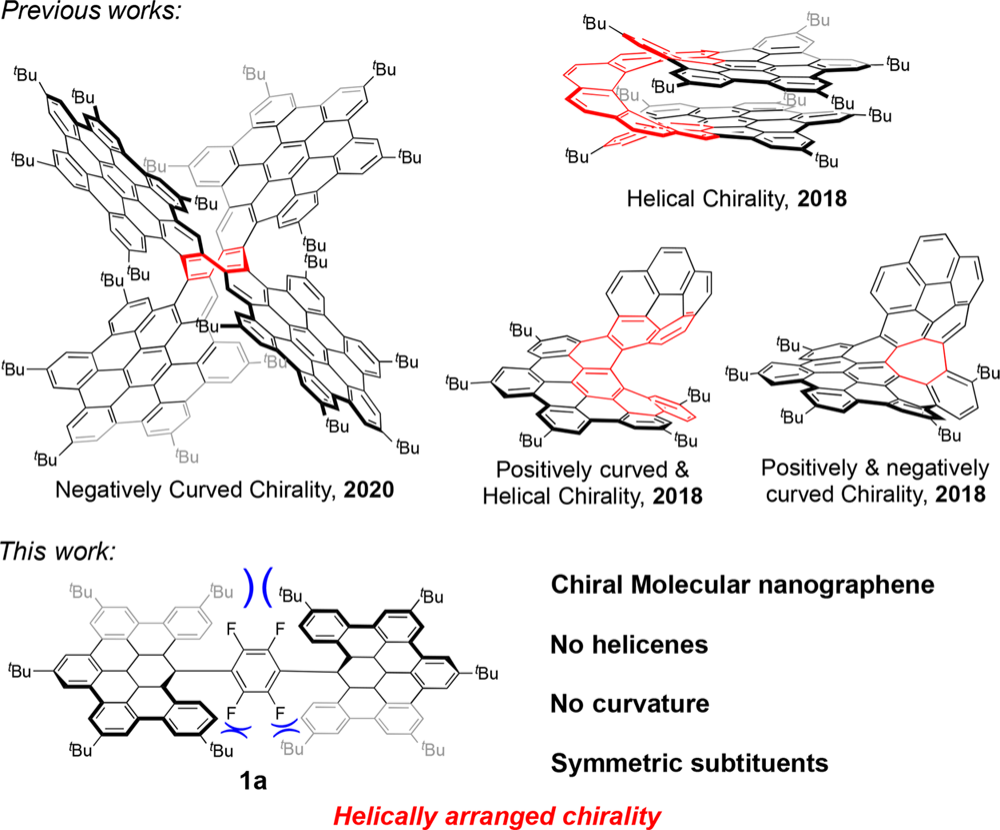
Chiral Molecular Nanographenes Previously Synthesized in Our Group and New Structure of Nanographene **1a**

Recently, our research group described the formation of molecular nanographenes with chirality derived from the presence of one or both aforementioned morphological defects (Chart 1).^19^ Herein, we describe the synthesis of a new family of chiral molecular nanographenes **1a**–**c** lacking both helicene and curvature features. Interestingly, nanographene **1a** is symmetrically substituted, and its chirality stems from a chiral axis formed as a result of the helical arrangement of the different moieties of the molecule.^20^ Actually, the kinetics of the racemization in **1a** is related to the steric rotational barriers existing between the two polyaromatic fragments and also with the central tetrafluorobenzene ring, resulting in a singular new type of orthogonally arranged chiral molecular nanographenes.

## Results and Discussion

### Synthetic Procedure

**Scheme 1:**
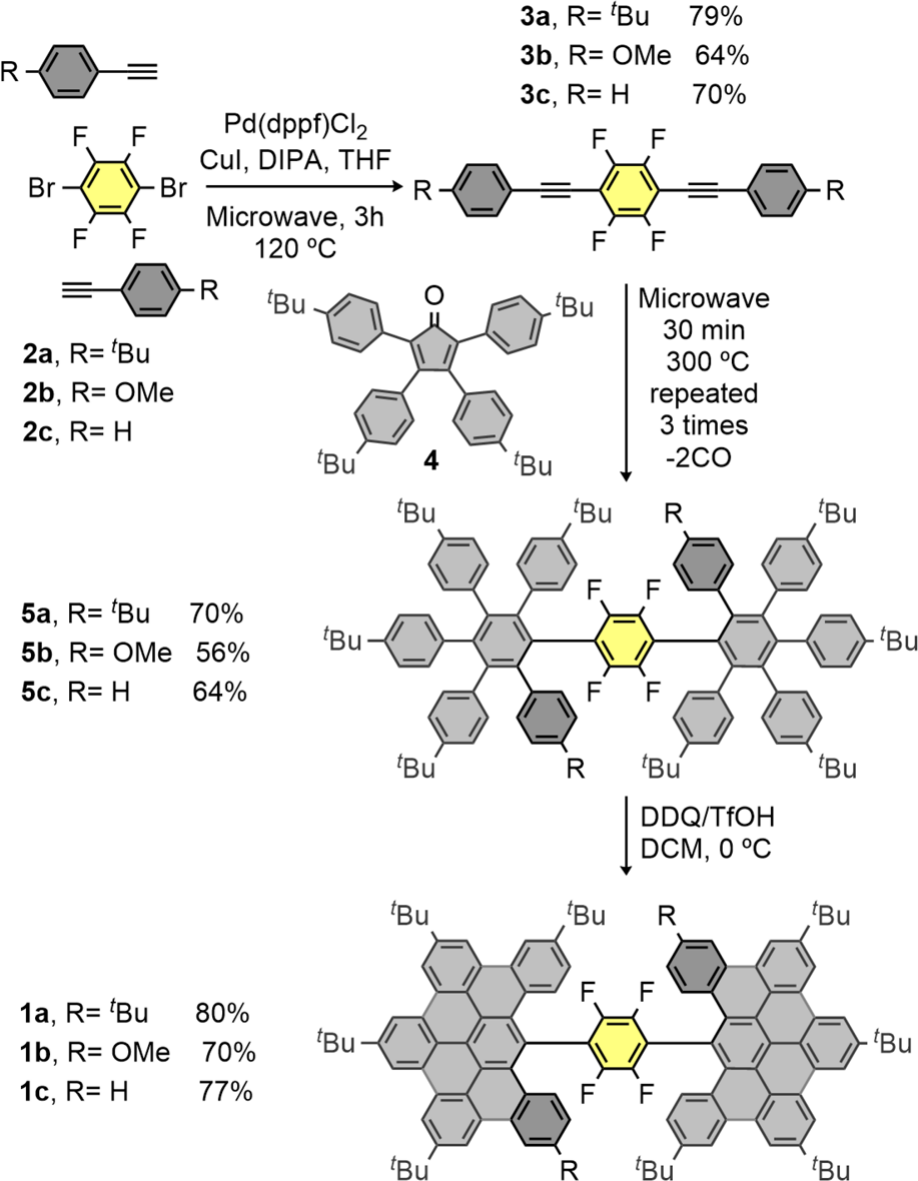
General Synthetic Pathway toward Molecular Nanographenes **1a**–**c**

Our synthetic strategy is based on three main synthetic steps involving Sonogashira coupling, Diels–Alder cycloaddition plus retrochelotropic reaction with carbon monoxide extrusion, and a final Scholl cyclodehydrogenation to achieve the graphitized molecular structures **1a**–**c** (Scheme 1).

The first step involves a double Pd-catalyzed Sonogashira cross-coupling reaction of two equivalents of a suitably substituted phenylacetylene **2a**–**c** and 1,4-dibromotetrafluorobenzene. The resulting bis[aryl(ethynyl)]tetrafluorobenzenes **3a**–**c** are able to undergo a 2-fold [4 + 2] cycloaddition reaction with cyclopentadienone **4** endowed with *tert*-butyl groups in order to improve solubility, thus affording polyaromatic compounds **5a**–**c** in good yields. The final step is the Scholl cyclodehydrogenation of polyphenylenes **5a**–**c** by reaction with dichlorodicyano-*p*-benzoquinone (DDQ) in the presence of triflic acid at 0 °C, which provides the graphene-like shape by forming eight aryl–aryl bonds in a single reaction step to afford **1a**–**c** in very good yields.

All final products and nonpreviously described intermediates have been characterized by ^1^H and ^13^C NMR, ^19^F-NMR, FT-IR, and high-resolution mass spectrometry. NMR spectra of **1a** reveal only three ^*t*^Bu groups with 2:2:1 relative intensities and only one ^19^F signal (see Figure S7 in the Supporting Information). These signals correspond to a quarter of the molecule, revealing its highly symmetric nature with three orthogonal *C*_2_ rotational axes. Because of the lack of symmetry planes and inversion centers, this compound is framed in the chiral space-group of symmetry *D*_2_. Moreover, NMR spectra of **1b** and **1c** (see SI, figures S15 and S22) show a 1:1 mixture of isomers. The presence of four 1:1:1:1 ^*t*^Bu signals for each isomer that correspond to a half molecule indicates a loss of symmetry compared to **1a**. These compounds have two *C*_2_ rotational axes. As they have neither symmetry planes nor inversion points, they are included in the chiral space-group of symmetry *C*_2_.

**Figure 1:**
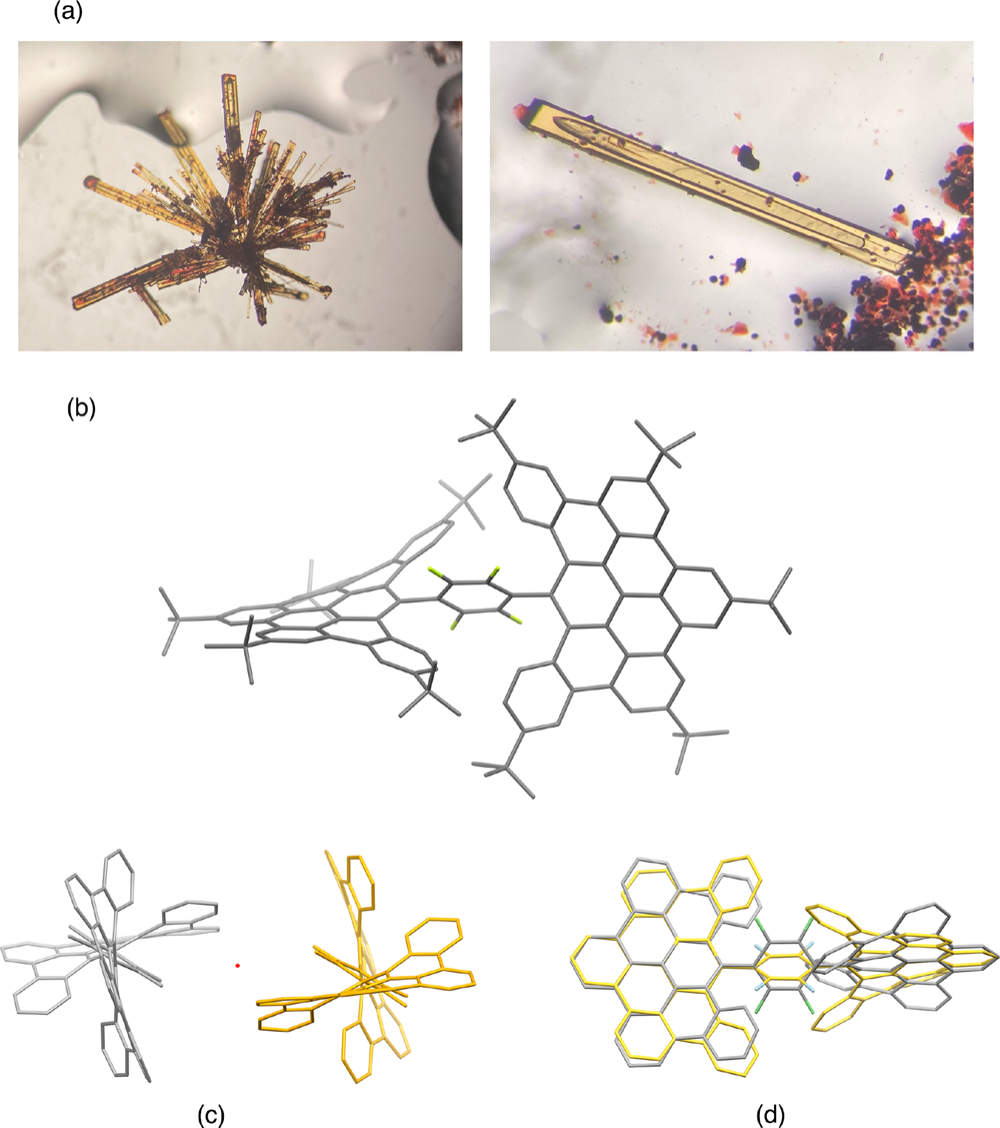
(a) Aggregate of crystals of **1a** (left) and individual needle with fluid inclusion (right); (b) X-ray crystal structure of **1a**; (c) view of the cores of two adjacent enantiomers related by an inversion center (in red); and (d) superimposed cores of both enantiomers of **1a**. Hydrogen atoms and solvent molecules have been omitted for clarity.

Additionally, the structure of **1a** has been unequivocally established by single-crystal X-ray diffraction (see section 4 of the Supporting Information). Crystals of **1a** with acicular habit (see Figure 1a) were obtained from a dichloroethane solution. The compound crystallizes in the centrosymmetric monoclinic *C*2/*c* space group, with one molecule in the asymmetric unit surrounded by several solvent ones (dichloroethane and water) displaying various degrees of disorder. As discussed before, **1a** displays chirality (Figure 1c,d) because of the disposition of the two DBPP moieties around the central tetrafluorobenzene ring, with dihedral angles of 54.04° (C1–C6 with C7–C12) and 50.87° (C1–C6 with C63–C68). Additionally, the DBPP fragments deviate greatly from the planarity (Figure 1b), adding also helicity to the structure. The supramolecular arrangement of the molecules is achieved by C–H···π interactions, as the presence of the bulky *tert-*butyl substituents prevents the formation of π–π stacking (see Table S3 and Figures S31 and S32 for details).

**Figure 2:**
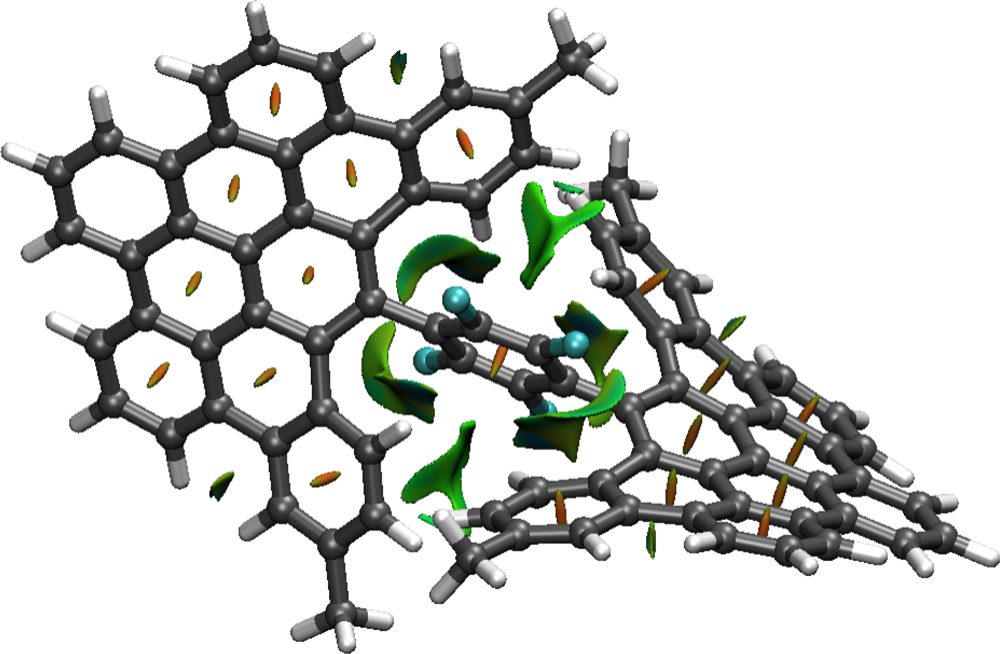
Contour plots of the reduced density gradient isosurfaces (density cutoff of 0.04 au) for compound **1a′**. The greenish surfaces indicate attractive noncovalent interactions (for different orientations, see Figure S45 in the Supporting Information).

Density functional theory (DFT) calculations on the model system **1a′**, where the bulky *tert*-butyl groups were replaced by methyl groups (see section 9 in the Supporting Information), reveal the occurrence of stabilizing noncovalent C–H···π interactions in this novel nanographene. As clearly shown in Figure 2, there exist significant attractive interactions (greenish surfaces) between the central C_6_F_4_ fragment and the closest C–H bonds of the adjacent DBPP moieties. According to the second order perturbation theory (SOPT) of the natural bond order (NBO) method, this attractive interaction results from the π(C=C) → σ*(C–H) and σ(C–H) → π*(C=C) electronic delocalizations, whose associated stabilization energies, Δ*E*^(2)^, amount to −1.04 and −0.68 kcal/mol, respectively. The occurrence of these (4-fold) interactions is key for the stereoisomerism found in these species (see below).

### Stereoisomerism of Nanographenes **1a**–**c**

Despite the apparent lack of the typical asymmetry elements of chiral nanographenes, namely chiral centers, helixes, or curvature, nanographenes **1a**–**c** are chiral molecules. The existence of rotational barriers between the central tetrafluorobenzene ring and the two DBPP moieties and between both DBPPs generates a chiral axis around which the three planes containing each of the three components of the DBPP–C_6_F_4_–DBPP fragment are disposed in a helical arrangement. The symmetrical substitution pattern of the central C_6_F_4_ ring does not allow the application of the Cahn–Ingold–Prelog rules to set the priority of the substituents. Therefore, nanographenes **1a**–**c** cannot be formally considered as atropoisomeric molecules endowed with two chiral axes, as in the case of terphenyl derivatives atropoisomerism.^21^

**Figure 3:**
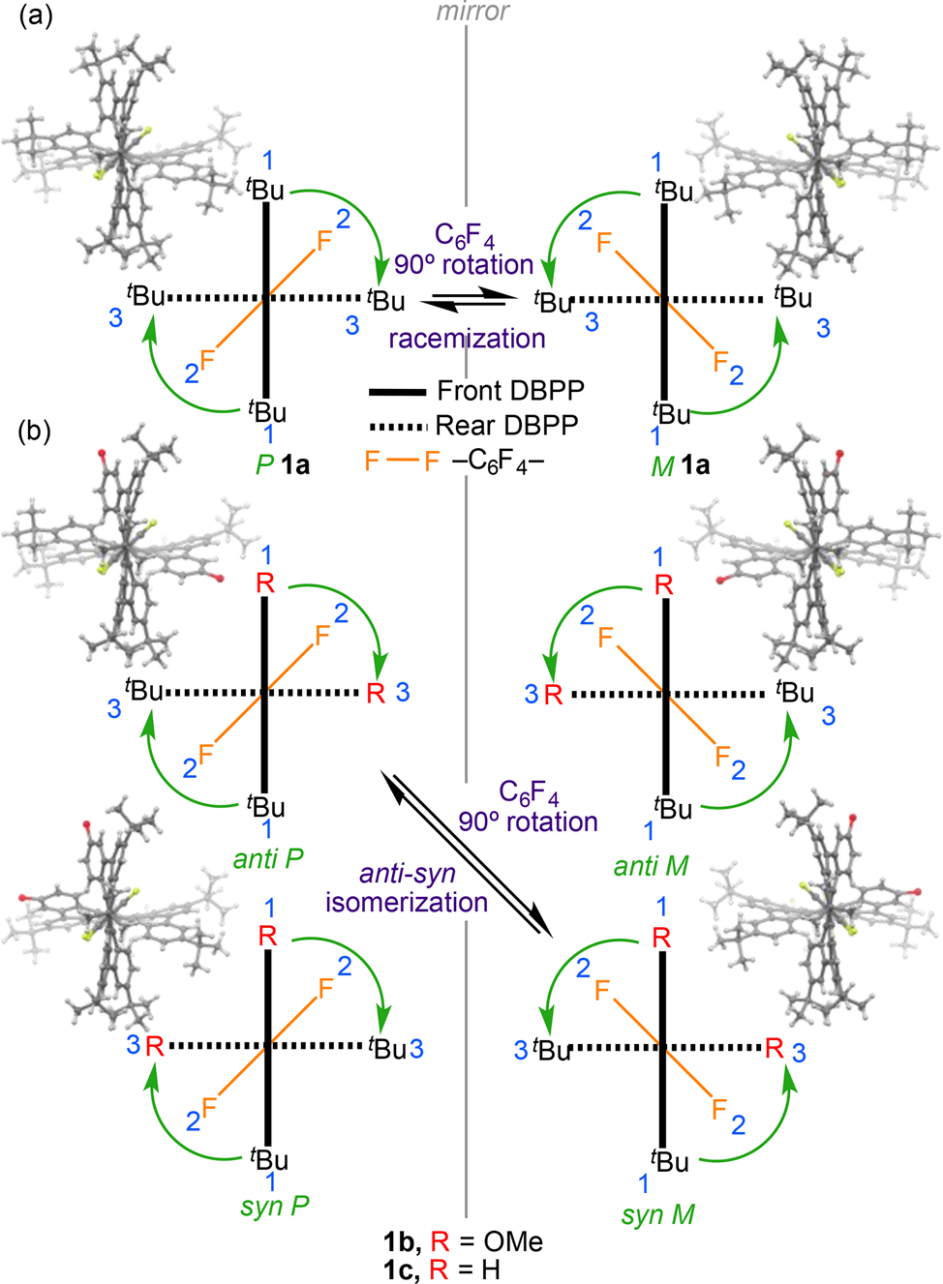
Newman-like projections of **1a**, **1b**, and **1c** (priorities from front to rear, blue numbers; absolute configurations, green arrows). (a) Both **1a** enantiomers absolute configuration. (b) **1b** and **1c***anti* and *syn* stereoisomers.

The rotational barrier involving the two DBPPs is hampered by the occurrence of additional noncovalent interactions between the closest aryl groups of both DBPPs (see Figure 2), which lock these moieties in an orthogonal array. Furthermore, the existence of additional rotational barriers between the central C_6_F_4_ ring and each DBPP unit by steric hindrance enforces the tetrafluorobenzene ring to adopt a +45 or −45° angle, thus giving rise to the existence of stereoisomers for nanographenes **1a**–**c** (Figure 3). In this way, nanographene **1a** has two enantiomeric structures that could be resolved by chiral HPLC (see Figure S39). By establishing priorities from front to rear, the absolute configuration of these enantiomers can be described by the “helical” arrangement of the fragments around the chiral axis, i.e., *P* if DBPP–C_6_F_4_–DBPP planes have clockwise rotation or *M* in the opposite scenario (Figure 3a). The presence of a substituent different from the ^*t*^Bu group in each DBPP (**1b**, R = OMe; **1c**, R = H) results in the appearance of two new stereoisomers: *anti* when the substituents are placed in different sides of the C_6_F_4_ plane, and *syn* when they are located at the same side (Figure 3b). Interestingly, nanographenes **1b** and **1c** were isolated as a 1:1 isomeric mixture, determined by ^1^H NMR, when the Scholl reaction was performed at 0 °C. DFT calculations on a model of **1c** indicate that the corresponding *syn* and *anti* isomers are nearly degenerate (ΔΔ*E* = 0.8 kcal/mol, favoring **1c′-***syn*), which is consistent with the experimental results. The 90° rotation of the central C_6_F_4_ ring from +45° to −45° leads to the racemization of nanographene **1a** (Figure 3a). However, the same 90° rotation in nanographenes **1b** and **1c** gives rise to the *anti*–*syn* isomerization with a change in the absolute configuration, but not involving racemization (Figure 3b).

Symmetrically substituted nanographenes with axial chirality have previously been described by Campaña et al.^22^ and Wang et al.^23^ In these examples, chirality stems from the strain arising from their planar conformation which forces these nanoribbons to twist around the chiral axis. Additionally, the absolute configuration of these twisted nanoribbons has been assigned by the presence of helicenes in their respective structures. However, axial chirality of nanographenes **1a**–**c** arises from rotational barriers between DBPP–C_6_F_4_–DBPP fragments, and it represents the first example of “atropoisomeric-like” chirality in symmetrically substituted nanographenes.

### ^1^H NMR Study of the *anti–syn* Isomerization (C_6_F_4_ Ring Rotational Barrier)

**Figure 4:**
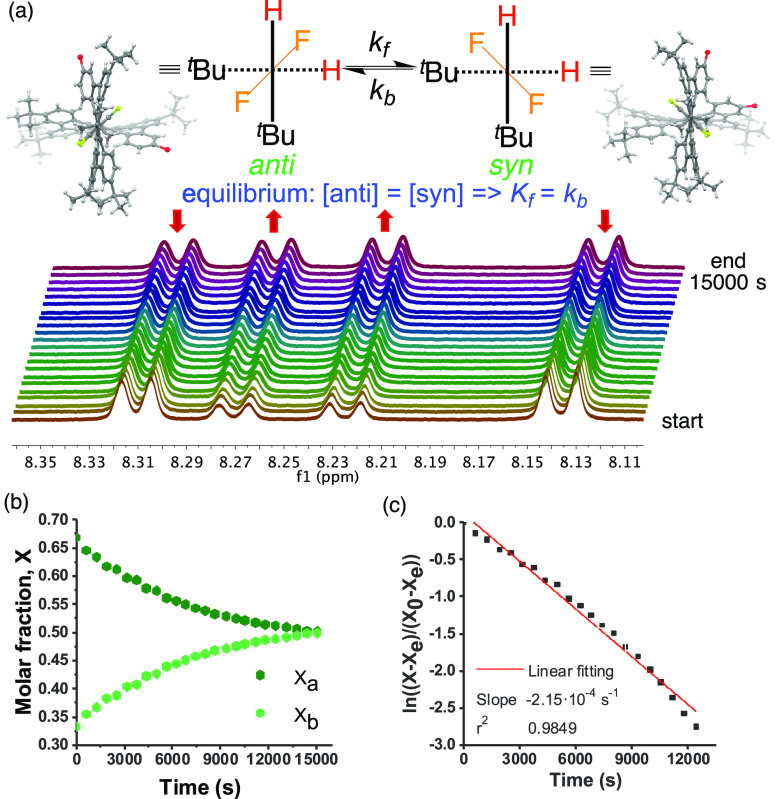
(a) ^1^H NMR isomerization experiment of **1c** performed in CH_2_Cl_4_ at 40 °C. Each ^1^H NMR spectrum was collected every 10 min approximately. (b) Variation of the isomers concentration vs *t*. (c) Fitting of ln((*χ*_a_ – χ_eq_)/(χ_0_ – χ_a_)) vs *t*.

As previously mentioned, compound **1c** (R = H) was isolated as 1:1 *anti-syn* isomeric mixture when the Scholl reaction of polyphenylene **5c** was carried out at 0 °C. However, at −65 °C, the Scholl reaction of **5c** led to a 70:30 *anti*/*syn* isomeric mixture (assigned by 2D NMR, see section 5 of the Supporting Information) with noncomplete conversion (see Figure S34). After workup to remove the DDQ, the mixture was warmed at 40 °C and monitored by ^1^H NMR every 10 min approximately. Under these conditions, the ratio of isomers decreases over time until reaching 50:50 at equilibrium (Figure 4a). The kinetic constants determined for the *anti*–*syn* isomerization are *k*_f_ = *k*_b_ = 1.08 × 10^–4^ s^–1^. According to the Eyring equation, the rotational barrier of the C_6_F_4_ central ring is Δ*G*^⧧^ = 24.6 kcal/mol at 40 °C with a half-life of *t*_1/2_ = 107 min (Figure 4b,c).

**Figure 5:**
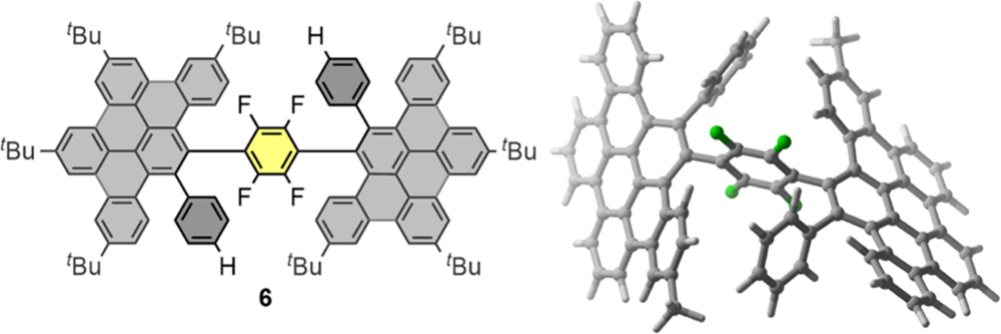
Structure of compound **6** and DFT calculations showing orthogonal arrangement of the DBPP–C_6_F_4_–DBPP fragments (^*t*^Bu groups were omitted for clarity).

Moreover, when the Scholl reaction of **5c** was carried out at −78 °C, it led to a unique compound **6** (Figure 5), characterized by ^1^H, ^13^C, ^19^F, and 2D NMR (see Figure S25). The lack of stereogenic elements in **6** is because the C–C bond involving the unsubstituted phenyl group was not formed in any DBPP, allowing the free rotation of the three DBPP–C_6_F_4_–DBPP fragments. In this case, the most stable conformation calculated by DFT (Figure 5) shows a nonchiral orthogonal arrangement, in contrast to the helical arrangement of chiral isomers **1c**.

### Electrochemical Properties of Nanographenes **1a**–**c**

The electrochemical properties of nanographenes **1a**–**c** were explored by cyclic voltammetry in atoluene/acetonitrile 4:1 mixture with tetrabutylammonium hexafluorophosphate as supporting electrolyte and Ag/AgNO_3_ as reference electrode at room temperature. Table 1 shows their respective reduction and oxidation potentials vs Fc/Fc^+^ compared to hexa-*tert*-butylhexa-*peri*-hexabenzocoronene (^*t*^Bu-HBC).

**Table 1:** First Oxidation and Reduction Potentials of ^*t*^Bu-HBC and Nanographenes **1a**–**c** vs Fc/Fc^+^^a^

compound	*E*^1^_red_ (V)	*E*^1^_ox_ (V)
^*t*^Bu-HBC	–2.26	0.80
**1a**	–2.42	0.92
**1b**	–2.39	0.94
**1c**	–2.34	0.97

a Measurements carried out in toluene/acetonitrile 4:1 mixture at room temperature using tetrabutylammonium hexafluorophosphate as supporting electrolyte, a glassy carbon as working electrode, platinum wire as counter electrode, and Ag/AgNO_3_ as reference electrode.

Molecular nanographenes **1a**–**c** show nonreversible first reduction waves at −2.42, −2.39, and −2.34 V vs Fc/Fc^+^, respectively, and quasi-reversible first oxidation waves at 0.92, 0.94, and 0.97 V vs Fc/Fc^+^. Compared to ^*t*^Bu-HBC, these compounds are poorer electron acceptors and electron donors as a consequence of the more π-extended structure of ^*t*^Bu-HBC. This fact suggests that the conjugation between the DBPP–C_6_F_4_–DBPP is hampered as a result of the noncoplanarity of these fragments. The observed electrochemical trend nicely correlates with the DFT-computed energy of the corresponding HOMO (the orbital from which the electron is released): −5.54 eV (**1a′**) < −5.21 eV (^*t*^Bu-HBC), thus showing that a more stabilized HOMO (i.e., more negative) is translated into a higher oxidation potential.

### Spectroscopic Properties of Nanographenes **1a**–**c**: Absorption, Fluorescence, and Optical Energy Gap

**Figure 6:**
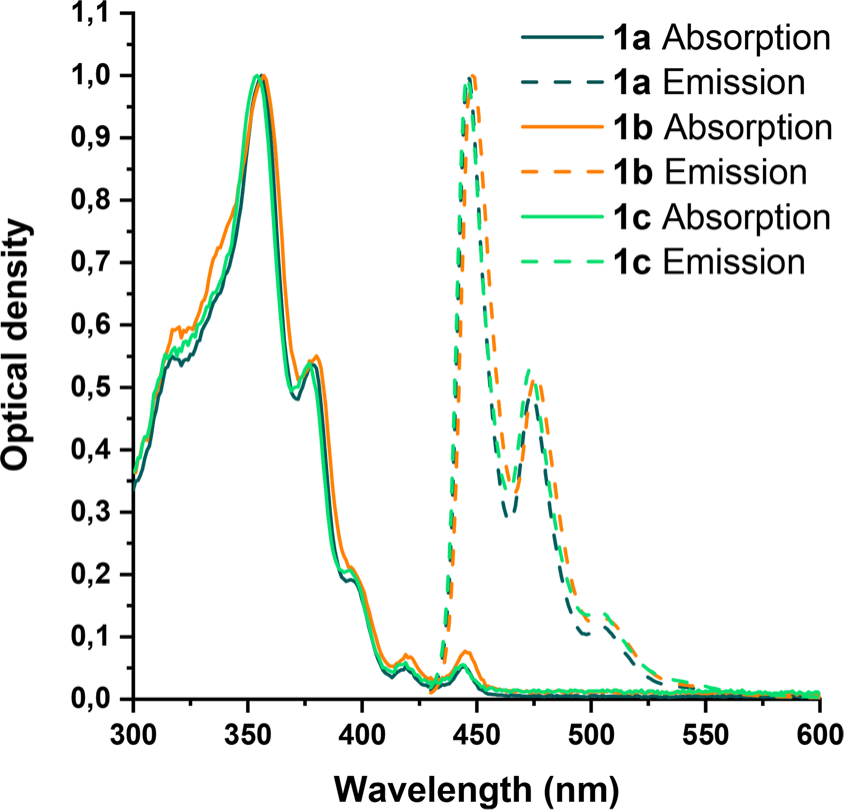
Normalized absorption (solid lines) and emission (dashed lines) spectra of **1a**–**c** in CHCl_3_ recorded at room temperature.

The UV–vis absorption and emission spectra of molecular nanographenes **1a**–**c** are shown in Figure 6. The wavelengths of the absorption and emission maxima are collected in Table 2.

**Table 2:** UV–Vis Absorption and Emission Spectra of **1a**–**c**^a^

compound	absorption λ_abs_^max^ (nm)	emission λ_em_^max^ (nm)	*E*_0–0_ (eV)^c^
**1a**	317, 356, 378, 395, 419, 444	446, 474, 505	2.79 (2.79)
**1b**	320, 357, 380, 397, 419, 445	448, 476, 507	2.77 (2.78)
**1c**	318, 354, 377, 395, 419, 444	446, 473, 504	2.79 (2.79)
^*t*^Bu HBC^b^	344, 360, 390, 439, 441, 443	493, 519, 553	2.65 (2.69)

a Measurements carried out in CHCl_3_ at room temperature.

b Data extracted from ref (19b).

c Values within parentheses are the energy gaps calculated from the corresponding intersections between the normalized absorption and emission spectra of each compound (see the Supporting Information, section 7).

The absorption spectra of molecular nanographenes **1a**–**c** show similar shapes and energies with three sharp absorptions in the UV region (**1a**: 317, 356, and 378 nm; **1b**: 320, 357, and 380 nm; **1c**: 318, 354, and 377 nm) and three weak bands in the vis region (**1a**: 395, 419, and 444 nm; **1b**: 397, 419, and 445 nm; **1c**: 395, 419, and 444 nm). In comparison, the spectrum of ^*t*^Bu-HBC^19b^ shows a similar shape, with three sharp absorptions in the UV region (344, 360, and 390 nm) and weak bands in the vis region (439, 441, and 443 nm). The bands of the spectra of ^*t*^Bu-HBC are red-shifted in comparison to nanographenes **1a**–**c** because of the more conjugated structure of ^*t*^Bu-HBC, which is in agreement with that observed in the electrochemical analysis.

**Figure 7:**
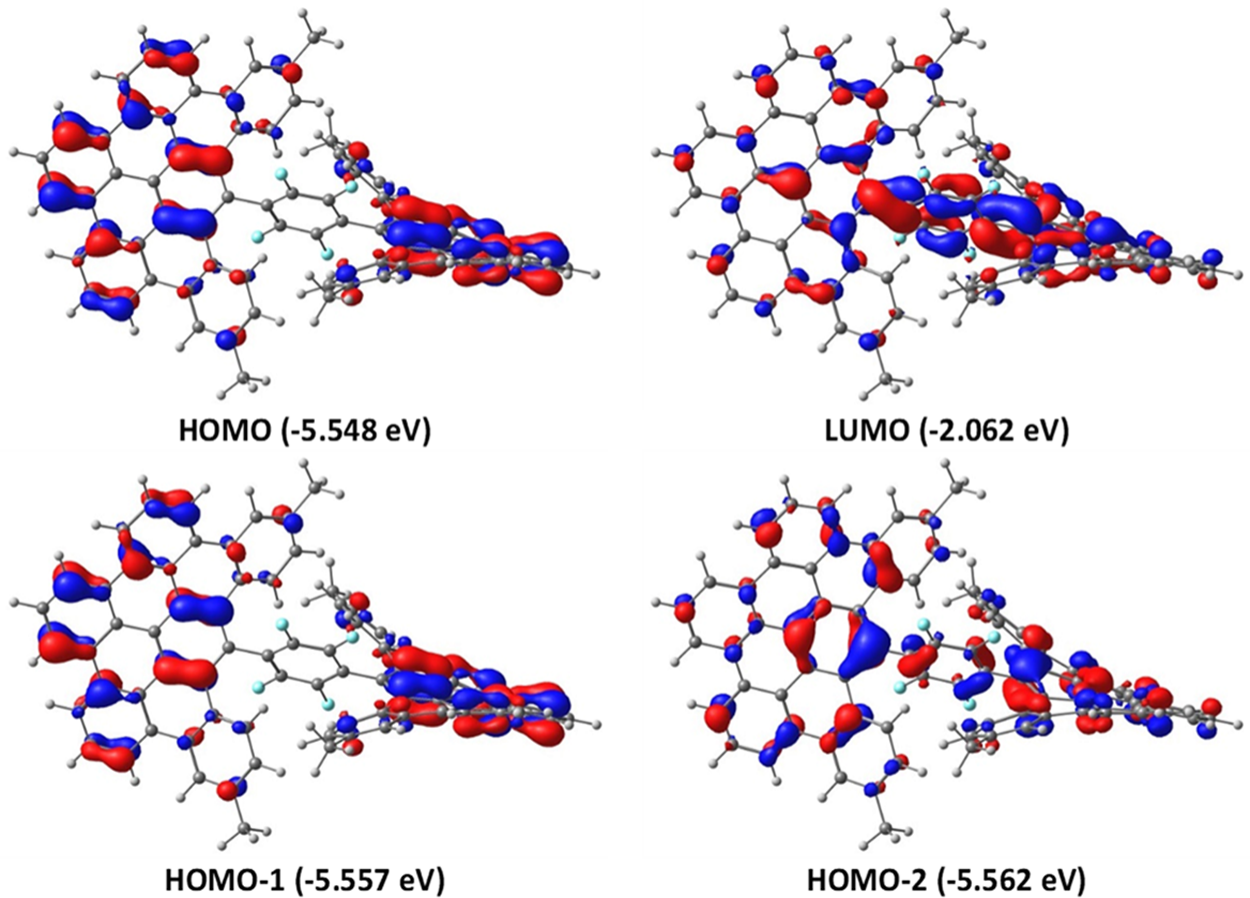
Computed molecular orbitals for **1a′** involved in the main UV/vis absorptions (isosurface value of 0.03 au).

Time-dependent (TD) DFT calculations were carried out on the model **1a′** to determine the nature of the vertical transitions associated with the observed UV/vis absorptions. Our TD-DFT calculations nicely reproduce the occurrence of the two bands at 419 and 444 nm (λ_calc_ = 418 and 420 nm, respectively), having a rather low oscillator strength (*f* = 0.028 and 0.020, respectively), which agrees with rather low ε observed experimentally (see Figure 6). These transitions are the result of the one-electron transition from the nearly degenerate HOMO and HOMO–1 (π-molecular orbitals delocalized in both DBPP moieties with no measurable coefficient in the central C_6_F_4_ fragment, see Figure 7) to the LUMO, respectively. Interestingly, the LUMO is a π*-molecular orbital delocalized along the entire molecule because of the presence of twisted π-orbitals connecting the central aryl ring with the DBPP fragments. The more intense band at ca. 395 nm (λ_calc_ = 410 nm, *f* = 0.18) is assigned to the HOMO–2 → LUMO transition. In this occasion, the HOMO–2 does exhibit coefficients in the central C_6_F_4_ fragment (Figure 7), thus confirming that the electronic communication in this compound by π-conjugation is not entirely suppressed despite the lack of coplanarity of the DBPP–C_6_F_4_–DBPP fragments.

Emission spectra of nanographenes **1a**–**c** are also very similar. The spectra show three bands (**1a**: 446, 474, and 505 nm; **1b**: 448, 476, and 507 nm; **1c**: 446, 473, and 504 nm) in the vis region. Once again, in comparison to the emission spectra of ^*t*^Bu-HBC,^19b^ the bands are red-shifted in the case of ^*t*^Bu-HBC. In addition, the optical energy gap of nanographenes **1a**–**c** was calculated by the intersection of the absorption and the emission spectra (**1a**, 2.79; **1b**, 2.78; and **1c**, 2.79 eV). These energy gaps are higher than that of ^*t*^Bu-HBC (2.69 eV) because of the lower extension of the π-conjugation between the DBPP–C_6_F_4_–DBPP. The noncoplanarity of these fragments leads to a HOMO and LUMO spatial separation that has special interest in delayed fluorescence applications.

## Conclusions

The synthesis of a new family of molecular nanographenes (**1a**–**c**) constituted by two orthogonal DBPP units covalently connected through a tetrafluorobenzene ring is reported. Interestingly, the new nanographenes have a chiral nature stemming from the chiral axis existing along the whole molecule. As expected, X-ray crystal analysis of **1a** reveals the existence of both enantiomers cocrystallizing in a single monocrystal because of the disposition of the two DBPP moieties around the central tetrafluorobenzene ring.

The absolute configuration of the obtained enantiomers can be described by the helical arrangement of the fragments around the chiral axis DBPP–C_6_F_4_–DBPP. Furthermore, both enantiomers were resolved by chiral HPLC. Notably, replacement of a *t-*butyl group in each DBPP unit leads to a new pair of diastereoisomers (*syn*–*anti*) which are nearly degenerate and whose rotational barrier has been determined experimentally by ^1^H NMR from the Eyring equation to be Δ*G*^⧧^ = 24.6 kcal/mol at 40 °C with a half-life of *t*_1/2_ = 107 min.

The new series of compounds **1a**–**c** shows interesting electrochemical and photophysical properties. Density functional theory (DFT) calculations nicely predict the occurrence of the two bands at 444 and 419 nm in the UV–vis spectra stemming from the HOMO and HOMO–1 to the LUMO transitions, respectively. Interestingly, the LUMO orbital is delocalized along the entire molecule because of the presence of twisted π-orbitals connecting the central aryl ring with the DBPP fragments, which allows the electronic communication along the entire molecule.

By performing the Scholl reaction of **5c** at −78 °C, the free rotation of the three DBPP–C_6_F_4_–DBPP fragments in the readily formed compound **6** is possible because of the lack of the C–C bond in the unsubstituted phenyl group. In agreement with the aforementioned results, the most stable conformation calculated by DFT for **6** shows a nonchiral orthogonal arrangement, which is in sharp contrast to the helical arrangement observed for chiral compound **1c**.

These so-far unknown molecular nanographenes represent one step further in the family of chiral nanographenes and pave the way to an alternative methodology to obtain molecular nanographenes with control on their chemical structure and, therefore, on their chiral and optoelectronic properties.

## Supporting Information Available

Synthetic procedures, additional figures and schemes of physical properties and characterization data, computational details, and Cartesian coordinates of all species described in the text (PDF)

## Accession Codes

CCDC 2070237 contains the supplementary crystallographic data for this paper. These data can be obtained free of charge via www.ccdc.cam.ac.uk/data_request/cif, or by emailing data_request@ccdc.cam.ac.uk, or by contacting The Cambridge Crystallographic Data Centre, 12 Union Road, Cambridge CB2 1EZ, UK; fax: +44 1223 336033.
