# Supplementary material for: Helically Arranged Chiral Molecular Nanographenes
Source: J Am Chem Soc. 2021 Jul 20;143(30):11864–70. doi: 10.1021/jacs.1c05977 (PMC9490840; doi:10.1021/jacs.1c05977)
Supplement: Supplementary file 1 — ja1c05977_si_001.pdf [file ja1c05977_si_001.pdf]

Helically Arranged Chiral Molecular Nanographenes

Patricia Izquierdo-García,[†] Jesús M. Fernández-García,[†] Israel Fernández,[†] Josefina Perles,[#] Nazario Martín* [† ‡]

[†]Departamento de Química Orgánica I, Facultad de Ciencias Químicas, Universidad Complutense, 28040 Madrid, Spain.

[‡]IMDEA-Nanociencia, C/ Faraday, 9, Campus de Cantoblanco, 28049 Madrid , Spain.

[#]Single Crystal X-ray Diffraction Laboratory, Interdepartmental Research Service (SIdI), Universidad Autónoma, Campus de Cantoblanco, 28049 Madrid, Spain.

Table of Contents

1. General S2
2. Synthetic procedures S3
3. NMR and FT-IR spectra S9
4. Single Crystal X-ray structure determination S32
5. Isomerization barrier determination S38
6. HPLC separation S47
7. Photophysical study S48
8. Electrochemical study S52
9. Computational Details S58

1. General

Unless otherwise noted, all materials including solvents were obtained from commercial suppliers and used without further purification. 99% 1,4-Dibromo tetrafluorobenzene, 96% 4-*tert*-butylphenylacetylene, 98% phenylacetylene, 99% CuI and 98% DDQ from Aldrich, 98% 4-methoxyphenylacetylene, dichloro[1,1'-bis(diphenylphosphino)ferrocene]palladium(II) complex with dichloromethane (1:1) Pd 13% and 98% TfOH from Alfa Aesar. Tetra-2,3,4,5-tetrakis[4-(1,1-dimethylethyl)phenyl]2,4-cyclopentadien-1-one was prepared according to the procedure reported in the literature.[1] Unless otherwise noted, all reactions were performed with dry solvents (dried by filtration through alumina according to the method described)[2] and under an atmosphere of argon in dried glassware with standard vacuum-line techniques. Microwave reactions were performed in an Anton-Parr Monowave 300 microwave reactor. All work-up and purification procedures were carried out with reagent-grade solvents in air. Silica column chromatography was conducted with Scharlau 40-60 µm silica gel. Analytical thin-layer chromatography (TLC) was performed using E. Merck silica gel 60 F254 precoated plates (0.25 mm). The developed chromatogram was analyzed by UV lamp (254 nm and 365 nm). IR spectra were recorded on a FT-IR Nicolet Magna 750 spectrometer. The matrices used for MALDI-TOF were trans-2-[3-(4-*tert*-butylphenyl)-2-methyl-2-propenylidene]-malononitrile (DCTB) or 1,8-dihidroxi-9,10-dihydroanthracen-9-ona (dithranol) and mass analysis were performed in a Bruker Ultraflex II using a LTB MNL 106 laser source. ^{1}H NMR spectra were recorded at 700 (Bruker AVIII), 500 (Bruker AV), or 300 (Bruker AVIII), ^{19}F NMR spectra were recorded at 471 (Bruker AV) or 282 (Bruker AVIII) and ^{13}C NMR spectra were recorded at 126 (Bruker AV). Chemical shifts for ^{1}H NMR, ^{19}F NMR and ^{13}C NMR are expressed in parts per million (ppm) relative to the solvent. Data are reported as follows: chemical shift, multiplicity (s = singlet, d = doublet, dd = doublet of doublets, t = triplet, q = quartet, m = multiplet,), coupling constant (Hz), and integration.

[1] Lungerich, D.; Hitzenberger, J. F.; Marcia, M.; Hampel, F.; Drewello, T.; Jux, N. *Angewandte Chemie International Edition* **2014**, 53 (45), 12231.
[2] Pangborn, A. B.; Giardello, M. A.; Grubbs, R. H.; Rosen, R. K.; Timmers, F. J. *Organometallics* **1996**, 15 (5), 1518.

2. Synthetic procedures

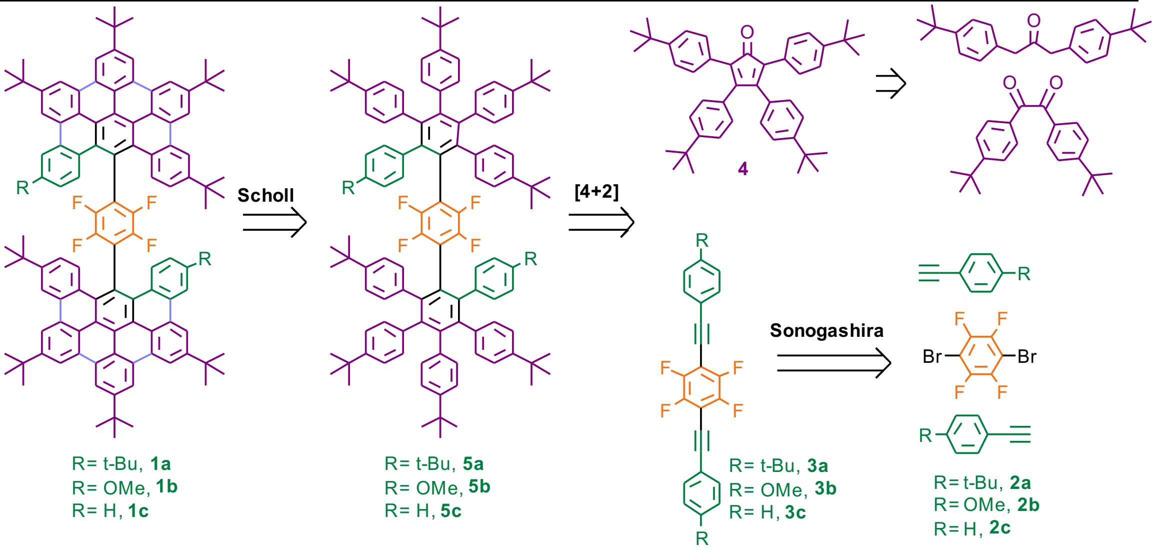

Scheme S1. Retrosynthetic scheme for nanografenes **1a**, **1b** and **1c**.

1,4-bis[(4-(*tert*-butyl)phenyl)ethynyl]tetrafluorobenzene 3a. To a dry 10 mL

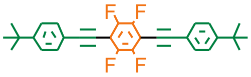

microwave reactor vial under argon atmosphere and provided with a magnetic stir bar, CuI (0.06 mmol, 12 mg, 0.4 equiv.), Pd(dppf)Cl_2 (0.03 mmol, 27 mg, 0.2 equiv.), 1,4-dibromotetrafluorobencene (0.16 mmol, 50 mg, 1 equiv.), 4-(*tert*-butyl)phenylacetylene **2a** (0.41 mmol, 64 mg, 2.5 equiv.), 1.7 mL of anhydrous previously deoxygenated THF and 0.4 mL of distilled DIPA were added. The reaction was carried out for 3 hours at 120 ºC and 600 rpm in a microwave reactor. The resulting crude was extracted with DCM and washed with NH_4Cl (50 mL), water (50 mL) and brine (50 mL). The organic phase was dried with $MgSO_4$, filtered through a silica gel plug column using dichloromethane as eluent and the solvent was removed under reduced pressure. The product was dissolved in the minimum amount of $CHCl_3$ and then methanol was added to obtain a precipitate that was filtered to reach **3a** as white solid (59.4 mg, 79%). ^{1}H NMR (300 MHz, Chloroform-d) δ= 7.54 (d, J = 8.5 Hz, 4H), 7.42 (d, J = 8.5 Hz, 4H), 1.34 (s, 18H). ^{19}F NMR (471 MHz, Chloroform-d) δ= -138.00 (s). ^{13}C{^{1}H} NMR (126 MHz, Chloroform-d) δ= 153.3, 146.6 (dm, $^{1}J_{C\text{-}F}$ = 243.5 Hz), 131.9, 125.7, 118.8, 104.9 (m) 103.4, 74.3, 35.1, 31.2. FT-IR (cm^{-1}) 3030, 2959, 2904, 2867, 2229, 2210, 1605, 1522, 1512, 1487, 980, 949, 835. HRMS: Calculated for $C_{30}H_{26}F_4$=462.1971, found=462.1958.

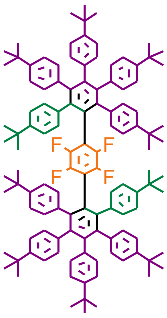

5a. To a 10 mL dry microwave reactor vial, provided with a magnetic stir bar, 2,3,4,5-tetrakis[4-(*tert*-butyl)phenyl]cyclopenta-2,4-dien-1-one **4** (0.34 mmol, 200 mg, 3.1 equiv.) and **3a** (0.09 mmol, 45 mg, 1 equiv.) were added. The reaction was carried out in a microwave reactor at 300 ºC and 600 rpm for 30 minutes, repeating these conditions for a total of four sequential times and removing carbon monoxide under vacuum between each repetition. The resulting crude was dissolved in the minimum amount of $CHCl_3$ and methanol

was added to obtain a precipitate that was filtered, affording **5a** as a white solid (110.9 mg, 70%). ^{1}H NMR (500 MHz, Chloroform-d) δ= 7.18 (d, *J* = 8.1 Hz, 4H), 6.83 (d, *J* = 8.3 Hz, 4H), 6.78 (d, *J* = 8.6 Hz, 12H), 6.68 (d, *J* = 8.3 Hz, 4H), 6.61 (d, *J* = 8.1 Hz, 4H), 6.59 – 6.48 (m, 12H), 1.25 (s, 36H), 1.08 – 1.07 (m, 54H). ^{19}F NMR (282 MHz, Chloroform-d) δ= -137.48 (s). $^{13}C\{^{1}H\}$ NMR (126 MHz, Chloroform-d) δ= 148.5, 147.7, 147.6, 143.1 (dm, $^{1}J_{C\text{-}F}$ =256.2 Hz), 142.6, 141.9, 141.3, 137.7, 137.6, 137.0, 132.39, 131.2, 131.0, 128.9, 125.6, 123.6, 123.2, 122.8, 119.0 (m), 34.5, 34.2, 31.6, 31.3. FT-IR (cm^{-1}) 3089, 3039, 2962, 2903, 2869, 1899, 1773, 1657, 1609, 1512, 1469, 1391, 1362, 1268, 1020, 978, 831. HRMS: Calculated for $C_{118}H_{130}F_{4}$=1623.0109, found=1623.0033.

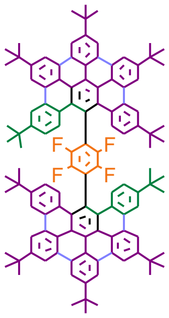

1a. To a 100 mL dry flask, under argon atmosphere and provided with a magnetic stir bar, **5a** (0.01 mmol, 20 mg, 1 equiv.), 37 mL of DCM and DDQ (0.54 mmol, 123 mg, 44 equiv.) were added. The reaction mixture was cooled to 0 °C and stirred for 5 minutes. Then, under Ar bubbling, TfOH was added dropwise (5.07 mmol, 760 mg, 412 equiv.) and the reaction was carried out at 0 °C maintaining the Ar bubbling for 25 minutes. After that time, a $HNaCO_3$ saturated solution was added to the reaction flask and the crude was extracted with DCM and washed twice with a $HNaCO_3$ saturated solution (40 mL) and twice with brine (40 mL). The organic phase was dried with $MgSO_4$ and the solvent was removed under reduced pressure. The crude was purified by aluminum oxide column chromatography using pentane as eluent, affording **1a** as a yellow solid (12.8 mg, 80%). ^{1}H NMR (300 MHz, Chloroform-d) δ= 9.16 (s, 4H), 9.11 (d, *J* = 1.6 Hz, 4H), 9.01 (d, *J* = 1.6 Hz, 4H), 8.81 (d, *J* = 1.9 Hz, 4H), 8.26 (d, *J* = 8.8 Hz, 4H), 7.64 (dd, *J* = 8.8, 1.9 Hz, 4H), 1.82 (s, 18H), 1.76 (s, 36H), 1.39 (s, 36H). ^{19}F NMR (282 MHz, Chloroform-d) δ= -137.26 (s). $^{13}C\{^{1}H\}$ NMR (126 MHz, Chloroform-d) δ= 150.5, 149.9, 149.8, 146.97 (dm, $^{1}J_{C\text{-}F}$ = 241.1 Hz), 132.3, 130.7, 130.2, 130.0, 128.3, 127.5, 126.3, 124.2 (m), 124.2, 123.3, 123.2, 122.6, 122.1, 122.0, 120.2, 119.3, 119.2, 118.8, 118.5, 35.9, 35.8, 35.1, 32.2, 32.1, 31.3, 29.9. FT-IR (cm^{-1}) 3082, 2952, 2921, 2852, 1608, 1588, 1479, 1463, 1374, 1246, 981, 871. HRMS: Calculated for $C_{118}H_{114}F_{4}$=1606.8857, found=1606.8866.

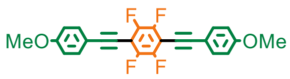

1,4-bis[(4-methoxyphenyl)ethynyl]tetrafluorobenzene 3b. To a dry 10 mL microwave reactor vial under argon atmosphere and provided with a magnetic stir bar, CuI (0.06 mmol, 12 mg, 0.4 equiv.), Pd(dppf)Cl_2 (0.03 mmol, 27 mg, 0.2 equiv.), 1,4-dibromotetra fluorobencene (0.16 mmol, 50 mg, 1 equiv.), 4-(methoxy)phenylacetylene **2b** (0.41 mmol, 54 mg, 2.5 equiv.), 1.7 mL of anhydrous previously deoxygenated THF and 0.4 mL of distilled DIPA were added. The reaction was carried out for 3 hours at 120 °C and 600 rpm in a microwave reactor. The resulting crude was extracted with DCM and washed twice with NH_4Cl (50 mL), twice with water (50 mL) and twice with brine (50 mL). The organic phase was dried with $MgSO_4$, filtered through a silica gel plug

column using dichloromethane as eluent and solvent was removed under reduced pressure. The resulting solid was dissolved in the minimum amount of $CHCl_3$ and then methanol was added to obtain a precipitate that was filtered to reach **3b** as a brown solid (42.3 mg, 64%). ^{1}H NMR (300 MHz, Chloroform-d) δ= 7.54 (d, *J* = 8.8 Hz, 4H), 6.91 (d, *J* = 8.8 Hz, 4H), 3.85 (s, 6H). ^{19}F NMR (282 MHz, Chloroform-d) δ= -138.3 (s). ^{13}C{^{1}H} NMR (126 MHz, Chloroform-d) δ= 160.9, 146.6 (dm, $^1J_{C-F}$ = 261.9 Hz), 133.8, 114.3, 113.9, 104.8 (m), 103.3, 73.9, 55.5. FT-IR (cm^{-1}) 3022, 3002, 2970, 2937, 2909, 2840, 2228, 2210, 1601, 1518, 1481, 1246, 1172, 1024, 965, 830. HRMS: Calculated for $C_{24}H_{14}F_4O_2$=410.0930, found=410.0910.

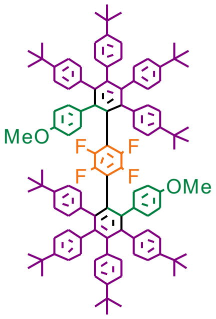

5b. To a 10 mL dry microwave reactor vial, provided with a magnetic stir bar, 2,3,4,5-tetrakis[4-(*tert*-butyl)phenyl] cyclopenta-2,4-dien-1-one **4** (0.23 mmol, 140 mg, 3.1 equiv.) and **3b** (0.07 mmol, 30 mg, 1 equiv.) were added. The reaction was carried out in a microwave reactor at 300 °C and 600 rpm for 30 minutes, repeating these conditions for a total of three times and removing carbon monoxide under vacuum between each repetition. The resulting crude was dissolved in the minimum amount of $CHCl_3$ and methanol was added to obtain a precipitate that was filtered, affording **5b** as a nude-colored solid (64.7 mg, 56%). ^{1}H NMR (500 MHz, Chloroform-d) δ= 7.00 (d, *J* = 8.4 Hz, 2H), 6.81 (d, *J* = 8.5 Hz, 2H), 6.77 – 6.73 (m, 4H), 6.66 – 6.48 (m, 12H), 3.73 (s, 3H), 1.23 (s, 9H), 1.10 (s, 9H), 1.07 (s, 9H), 1.06 (s, 9H). ^{19}F NMR (471 MHz, Chloroform-d) δ= -138.32 (s). ^{13}C{^{1}H} NMR (126 MHz, Chloroform-d) δ=157.7, 148.5, 147.7, 147.7, 147.6, 143.3 (dm, $^1J_{C-F}$ = 246.7 Hz), 142.8, 141.9, 141.4, 141.2, 141.1, 137.7, 137.6, 137.5, 137.0, 132.4, 131.8, 131.1, 131.0, 130.9, 130.5, 125.7, 123.5, 123.4, 123.2, 123.1, 119.3 (m), 112.4, 55.3, 34.5, 34.2, 34.2, 31.5, 31.3, 31.3. FT-IR (cm^{-1}) 3087, 3032, 2959, 2901, 2865, 1899, 1779, 1742, 1661, 1611, 1513, 1467, 1245, 977, 832. HRMS: Calculated for $C_{112}H_{118}F_4O_2$=1570.9068, found=1570.9106.

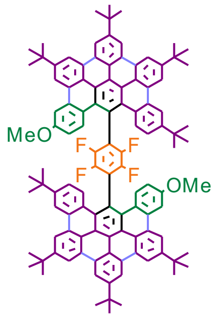

1b. To a 100 mL dry flask, under argon atmosphere and provided with a magnetic stir bar, **5b** (0.01 mmol, 20 mg, 1 equiv.), 38 mL of DCM and DDQ (0.56 mmol, 127 mg, 44 equiv.) were added. The reaction mixture was cooled to 0 °C and stirred for 5 minutes. Then, under Ar bubbling, TfOH was added dropwise (5.24 mmol, 790 mg, 412 equiv.) and the reaction was carried out at 0 °C maintaining the Ar bubbling for 25 minutes. After that time, a $HNaCO_3$ saturated solution was added to the reaction flask and the crude was extracted with DCM and washed twice with a $HNaCO_3$ saturated solution (40 mL) and twice with brine (40 mL). The organic phase was dried with $MgSO_4$ and the solvent was removed under reduced pressure. The crude was filtered through silica gel using DCM as eluent and solvent was removed under reduced pressure affording **1b** as a green-yellow solid (10.9 mg, 70%, 1:1 *anti*/*syn*). ^{1}H NMR (500 MHz,

Chloroform-d) δ= 9.20 – 9.09 (m, 8H), 9.02 (s, 1H), 8.99 (s, 1H), 8.93 (s, 1H), 8.91 (s, 1H), 8.82 (d, J = 2.0 Hz, 1H), 8.80 (d, J = 2.0 Hz, 1H), 8.30 – 8.23 (m, 4H), 8.21 (d, J = 9.2 Hz, 1H), 8.15 (d, J = 8.8 Hz, 1H), 7.62 (dd, J = 8.8, 2.0 Hz, 1H), 7.54 (dd, J = 8.8, 2.0 Hz, 1H), 7.15 (dd, J = 9.2, 2.6 Hz, 1H), 7.11 (dd, J = 9.2, 2.6 Hz, 1H), 3.95 (s, 3H), 3.93 (s, 3H), 1.81 (s, 18H), 1.77 – 1.74 (m, 36H), 1.39 – 1.38 (m, 18H). ^{19}F NMR (471 MHz, Chloroform-d) δ= -137.16 (s), -137.17 (s). ^{13}C{^{1}H}{^{19}F} NMR (126 MHz, Chloroform-d) δ= 159.0, 159.0, 150.5, 150.5, 149.9, 149.8, 147.0, 134.4, 134.4, 132.3, 132.3, 130.7, 130.6, 130.6, 130.5, 130.2, 130.1, 130.0, 129.9, 129.8, 129.7, 129.2, 128.3, 128.2, 127.4, 127.4, 126.5, 126.5, 126.4, 124.6, 124.4, 124.2, 124.1, 123.4, 123.4, 123.2, 123.2, 122.9, 122.9, 122.7, 122.7, 122.6, 122.1, 122.0, 121.9, 120.1, 120.1, 119.8, 119.7, 119.4, 119.3, 119.2, 118.8, 118.7, 118.6, 118.5, 118.3, 119.9, 111.6, 108.2, 107.7, 55.6, 55.5, 35.9, 35.8, 35.1, 32.1, 32.1, 31.4, 31.3. FT-IR (cm^{-1}) 3075, 2960, 2909, 2865, 1785, 1722, 1610, 1589, 1458, 1372, 1248, 1044, 979, 870. HRMS: Calculated for $C_{112}H_{102}F_4O_2$=1554.7816, found=1554.7840.

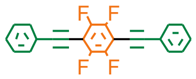

1,4-bis(phenylethynyl)tetrafluorobenzene 3c. To a dry 10 mL microwave reactor vial under argon atmosphere and provided with a magnetic stir bar, CuI (0.13 mmol, 20 mg, 0.4 equiv.), Pd(dppf)Cl_2 (0.06 mmol, 50 mg, 0.2 equiv.), 1,4-dibromotetrafluorobencene (0.32 mmol, 100 mg, 1 equiv.), phenylacetylene **2c** (0.81 mmol, 80 mg, 2.5 equiv.), 3.3 mL of anhydrous previously deoxygenated THF and 0.9 mL of distilled DIPA were added. The reaction was carried out for 3 hours at 120 °C and 600 rpm in a microwave reactor. The crude was extracted with DCM and washed twice with NH_4Cl (40 mL), twice with water (40 mL) and twice with brine (40 mL). The organic phase was dried with $MgSO_4$, filtered through a silica gel plug column using dichloromethane as eluent and solvent was removed under reduced pressure. The product was dissolved in the minimum amount of $CHCl_3$ and then methanol was added to obtain a precipitate that was filtered to reach to **3c** as beige solid (80.0 mg, 70%). ^{1}H NMR (300 MHz, Chloroform-d) δ= 7.64 – 7.57 (m, 4H), 7.46 – 7.36 (m, 6H). ^{19}F NMR (471 MHz, Chloroform-d) δ= -137.70 (s). ^{13}C{^{1}H} (126 MHz, Chloroform-d) δ= 146.7 (dm, $^{1}J_{C-F}$ =254.2 Hz), 132.1, 129.9, 128.7, 121.8, 104.9, 103.2, 74.8. FT-IR (cm^{-1}) 3060, 2232, 2210, 1484, 1443, 975. HRMS: Calculated for $C_{22}H_{10}F_4$=350.0719, found=350.0703.

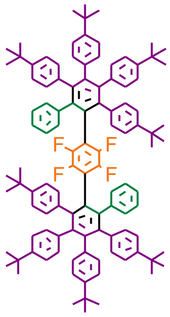

5c. To a 10 mL dry microwave reactor vial, provided with a magnetic stir bar, 2,3,4,5-tetrakis[4-(*tert*-butyl)phenyl]cyclopenta-2,4-dien-1-one **4** (0.35 mmol, 210 mg, 3.1 equiv.) and **3c** (0.11 mmol, 48 mg, 1 equiv.) were added. The reaction was carried out in a microwave reactor at 280 °C and 600 rpm for 30 minutes, repeating these conditions for a total of three times and removing carbon monoxide under vacuum between each repetition. The resulting crude dissolved in the minimum amount of $CHCl_3$ and methanol was added to obtain a precipitate that was filtered, affording **5c** as a nude-colored solid (141.0 mg, 82%). ^{1}H NMR (500 MHz, Chloroform-d) δ= 6.96 (d, J = 8.3 Hz, 4H), 6.88 (t, J = 7.2 Hz, 2H),

6.82 – 6.63 (m, 24H), 6.59 – 6.57 (m, 8H), 6.49 (d, *J* = 8.3 Hz, 4H), 1.24 (s, 18H), 1.10 – 1.02 (m, 54H). ^{19}F NMR (282 MHz, Chloroform-d) δ= -138.89 (s). ^{13}C{^{1}H}{^{19}F} NMR (126 MHz, Chloroform-d) δ= 148.25, 147.8, 147.7, 147.4, 143.3, 142.7, 141.9, 141.8, 141.4, 140.7, 139.7, 137.6, 137.5, 137.3, 137.0, 131.0, 130.9, 130.8, 130.2, 130.1, 126.7, 125.7, 125.1, 123.8, 123.3, 123.1, 123.0, 119.5, 34.5, 34.2, 34.1, 31.5, 31.3. FT-IR (cm^{-1}) 3090, 3051, 3028, 2960, 2905, 2865, 1896, 1779, 1661, 1603, 1513, 1462, 1391, 1361, 1269, 1022, 976, 832. HRMS: Calculated for $C_{110}H_{114}F_4$ =1510.8857, found=1510.8841.

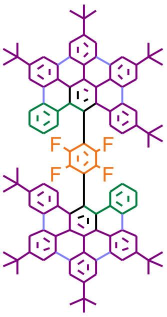

1c. To a 100 mL dry flask, under argon atmosphere and provided with a magnetic stir bar, **5c** (0.01 mmol, 20 mg, 1 equiv.), 40 mL of DCM and DDQ (0.58 mmol, 132 mg, 44 equiv.) were added, the reaction mixture was cooled to 0 °C and stirred for 5 minutes. Then, under Ar bubbling, TfOH was added dropwise (5.45 mmol, 820 mg, 412 equiv.) and the reaction was carried out at 0 °C maintaining the Ar bubbling for 25 minutes. After that time, a $HNaCO_3$ saturated solution was added to the reaction flask and the crude was extracted with DCM and washed twice with a $HNaCO_3$ saturated solution (40 mL) and twice with brine (40 mL). The organic phase was dried with $MgSO_4$ and the solvent was removed under reduced pressure. The crude was purified by silica gel chromatography column performed using a gradient from hexane:DCM (12:1) to hexane:DCM (6:1), solvent was removed under reduced pressure affording **5c** as a yellow solid (11.5 mg, 77%, 1:1 *anti*/*syn*). ^{1}H NMR (500 MHz, Chloroform-d) δ= 9.17 – 9.15 (m, 4H), 9.13 (s, 2H), 9.11 (s, 2H), 9.02 – 8.99 (m, 4H), 8.87-8.84 (m, 2H), 8.81 (d, *J* = 1.8 Hz, 1H), 8.80 (d, *J* = 1.8 Hz, 1H), 8.32 (d, *J* = 8.4 Hz, 1H), 8.29 (d, *J* = 8.4 Hz, 1H), 8.24 (d, *J* = 8.8 Hz, 1H), 8.15 (d, *J* = 8.8 Hz, 1H), 7.68 – 7.64(m, 2H), 7.61 (dd, *J* = 8.8, 1.8 Hz, 1H), 7.54 – 7.50 (m, 3H), 1.81 (s, 18H), 1.76 - 1.74 (m, 36H), 1.38 (s, 9H), 1.37 (s, 9H). ^{19}F NMR (471 MHz, Chloroform-d) δ= -137.35 (s), -137.37 (s). ^{13}C{^{1}H}{^{19}F} NMR (126 MHz, Chloroform-d) δ= 150.6, 150.5, 150.0, 150.0, 150.0, 149.9, 149.9, 146.9, 132.7, 132.6, 132.3, 132.2, 130.8, 130.7, 130.6, 130.6, 130.6, 130.3, 130.2, 130.1, 130.1, 130.0, 130.0, 129.9, 128.3, 128.2, 127.8, 127.7, 127.6, 127.5, 126.4, 126.3, 124.4, 124.3, 124.2, 124.1, 124.0, 123.5, 123.4, 123.3, 123.3, 123.2, 123.1, 123.1, 122.6, 122.1, 122.0, 121.9, 121.9, 120.2, 120.1, 119.5, 119.5, 119.4, 119.3, 119.3, 119.2, 119.0, 118.9, 118.7, 118.6, 118.5, 35.9, 35.8, 35.1, 32.2, 32.1, 31.4, 31.3. FT-IR (cm^{-1}) 3081, 2959, 2905, 2866, 1607, 1588, 1476, 1458, 1372, 1243, 979, 870. HRMS: Calculated for $C_{110}H_{98}F_4$=1494.7605, found=1494.7620.

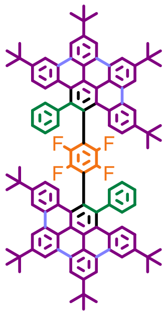

6. To a 100 mL dry flask, under argon atmosphere and provided with a magnetic stir bar, **5c** (0.01 mmol, 10 mg, 1 equiv.), 20 mL of DCM and DDQ (0.29 mmol, 66 mg, 44 equiv.) were added. The reaction mixture was cooled to -78 °C and stirred for 5 minutes. Then, under Ar bubbling, TfOH was added dropwise (2.72 mmol, 410 mg, 412 equiv.) and the reaction was carried out at -78 °C maintaining the Ar bubbling for 3 hours. After that time, a $HNaCO_3$ saturated solution was added to the reaction flask and the crude was extracted with DCM and washed twice with a $HNaCO_3$ saturated solution (20 mL) and twice with brine (20 mL). The organic phase was dried with $MgSO_4$ and filtered through silica gel using DCM as eluent. Solvent was removed under reduced pressure affording **6** as a yellow solid (7.41 mg, 75%). ^{1}H NMR (700 MHz, 1,1,2,2-Tetrachloroethane-d_2) δ= 9.16 (s, 1H), 9.15 (s, 1H), 9.01 (s, 1H), 8.99 (s, 1H), 8.77 (d, *J* = 2.1 Hz, 1H), 8.69 (d, *J* = 2.1 Hz, 1H), 7.86 (d, *J* = 8.6 Hz, 1H), 7.64 (d, *J* = 8.8 Hz, 1H), 7.36 (d, *J* = 7.4 Hz, 2H), 7.33 (dd, *J* = 8.6, 2.1 Hz, 1H), 7.23 (t, *J* = 7.4 Hz, 1H), 7.18 (t, *J* = 7.4 Hz, 2H), 7.15 (dd, *J* = 8.8, 2.1 Hz, 1H), 1.77 (s, 9H), 1.76 (s, 9H), 1.60 (s, 9H), 1.46 (s, 9H). ^{19}F NMR (282 MHz, 1,1,2,2-Tetrachloroethane-d_2) δ= -135.52. ^{13}C{^{1}H} (176 MHz, 1,1,2,2-Tetrachloroethane-d_2) δ= 149.6, 149.4, 149.4, 149.2, 144.1 (dm, $^1J_{C\text{-}F}$ = 236.1 Hz), 142.8, 142.3, 140.1, 131.0, 130.9, 130.6, 130.4, 130.3, 129.7, 129.3, 128.3, 128.1, 128.0, 127.8, 127.6, 127.4, 126.9, 125.4, 125.0, 123.7, 123.5, 122.7 (m), 122.0, 121.9, 121.3, 120.6, 120.2, 119.0, 118.9, 118.7, 118.4, 35.4, 34.9, 34.7, 31.8, 31.7, 31.4, 31.2. Calculated for $C_{110}H_{102}F_4$ = 1498.7918, found=1498.7570.

3. NMR and FT-IR spectra

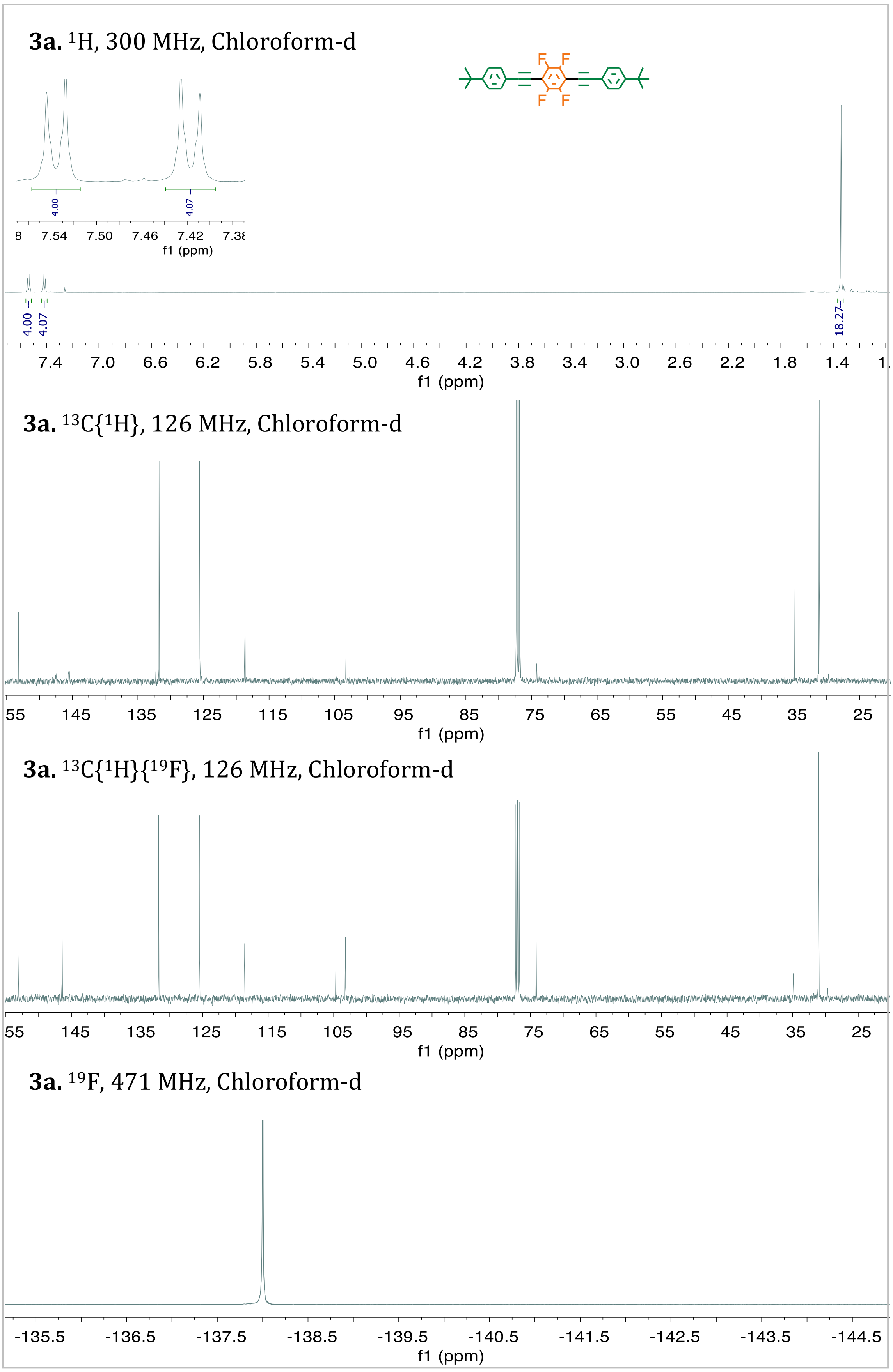

Figure S1. Compound **3a** ^{1}H, ^{13}C{^{1}H}, ^{13}C{^{1}H}{^{19}F} and ^{19}F NMR spectra.

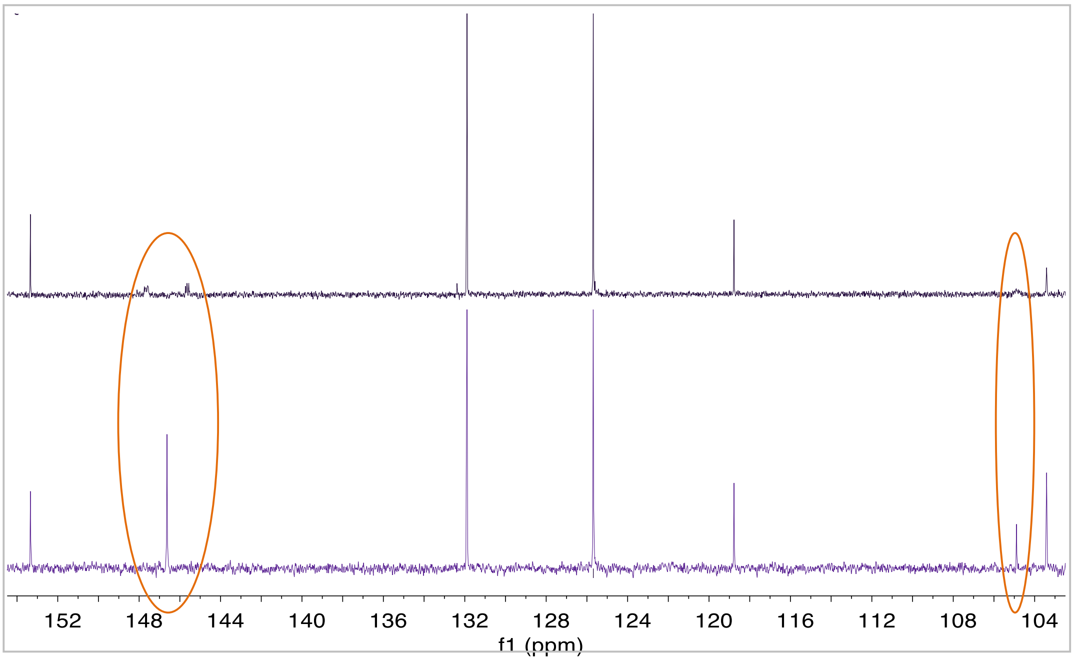

Figure S2. Determination of ^{13}C-^{19}F coupling constant (*J*) for compound **3a** by $^{13}C\{^1H\}$ y $^{13}C\{^1H\}\{^{19}F\}$ spectra comparison.

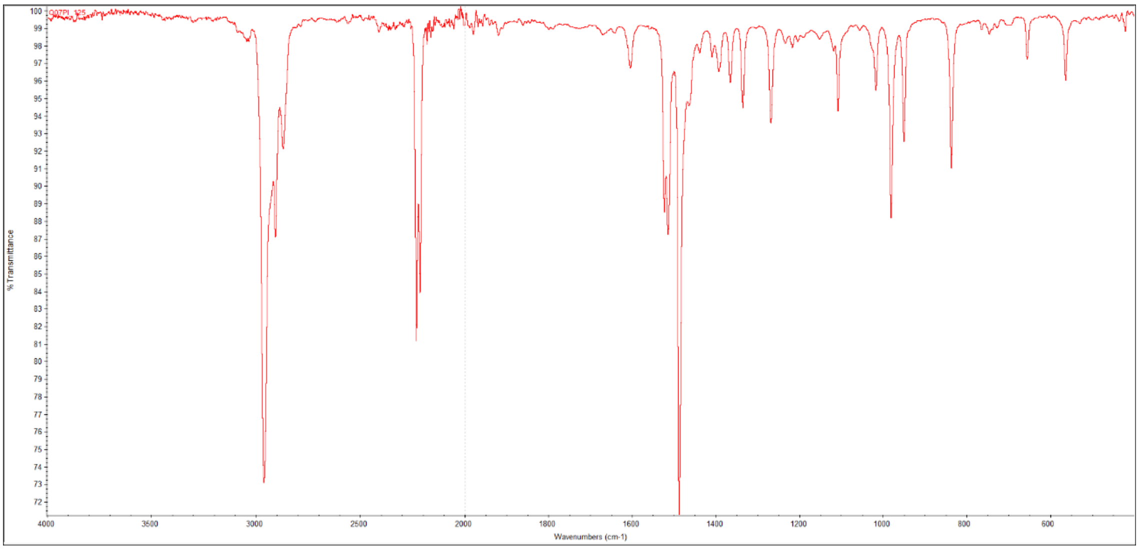

Figure S3. FT-IR spectrum of compound **3a**.

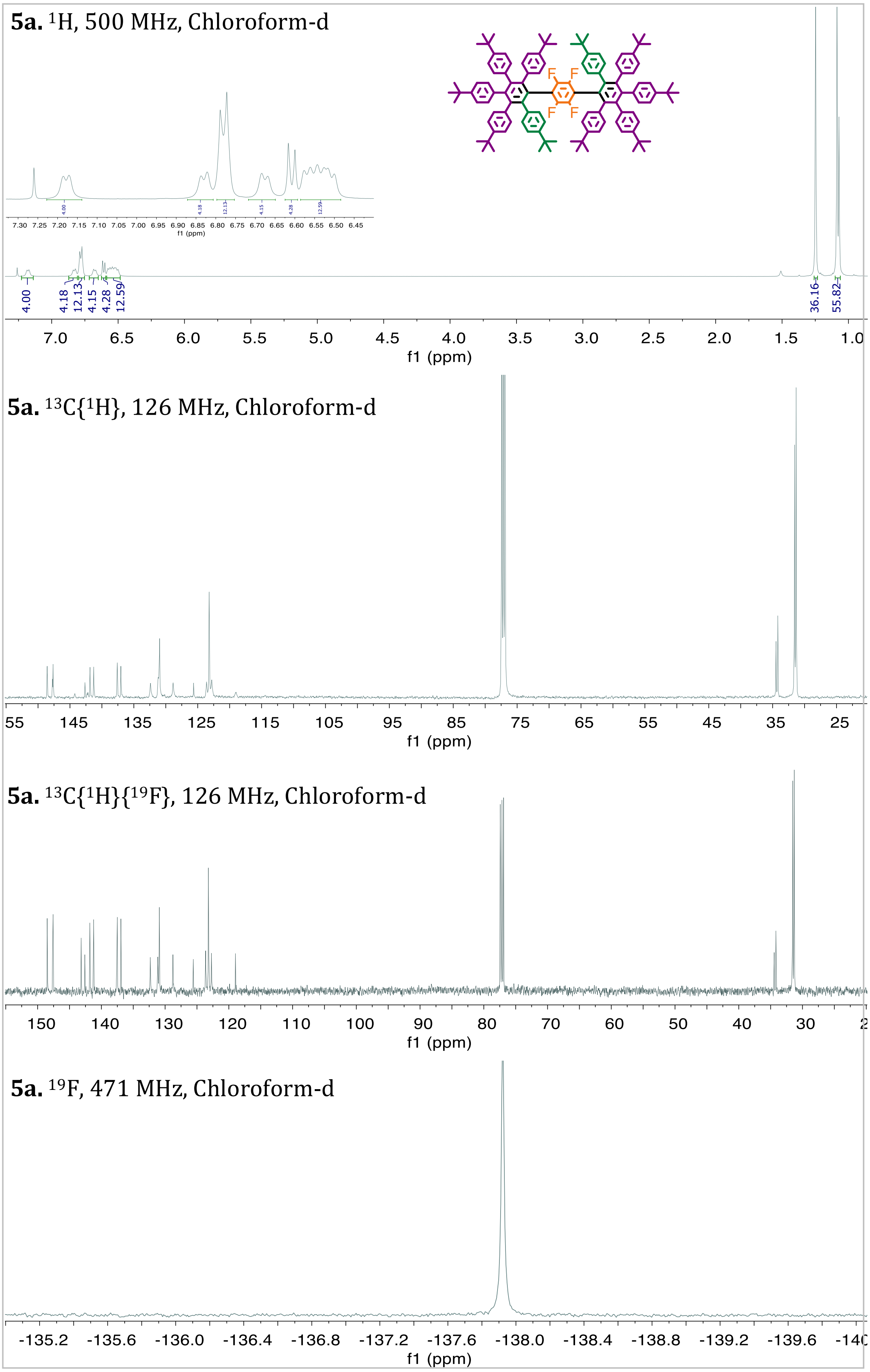

Figure S4. Compound **5a** ^{1}H, $^{13}C\{^{1}H\}$, $^{13}C\{^{1}H\}\{^{19}F\}$ and ^{19}F NMR spectra.

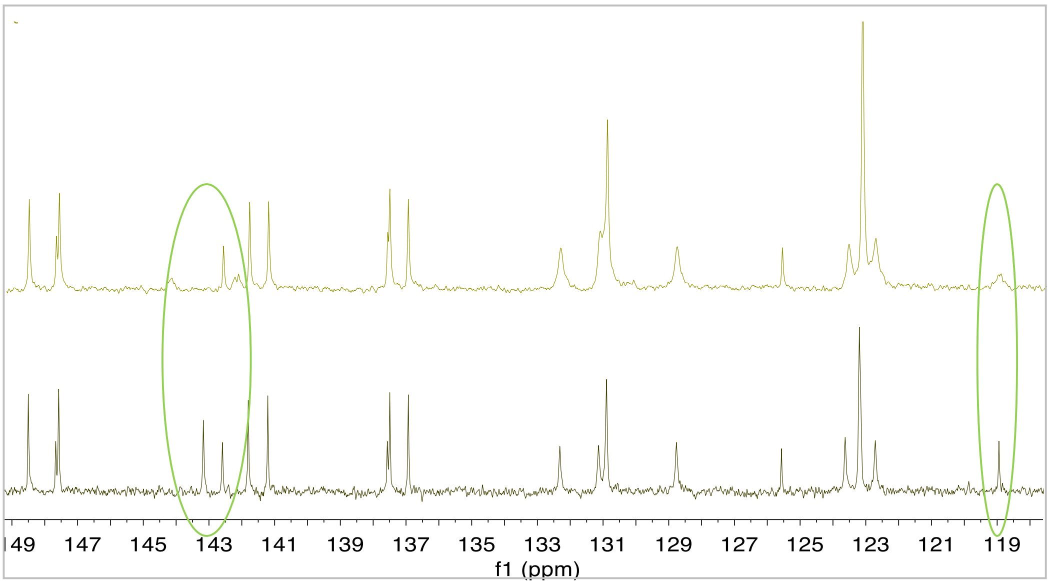

Figure S5. Determination of ^{13}C-^{19}F coupling constant (*J*) for compound **5a** by $^{13}C\{^1H\}$ y $^{13}C\{^1H\}\{^{19}F\}$ spectra comparison.

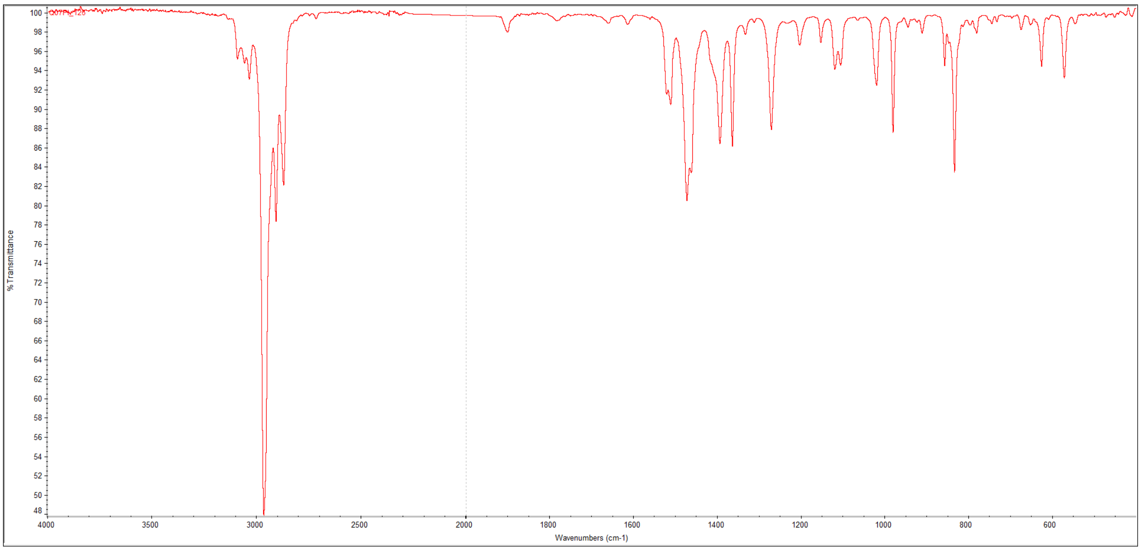

Figure S6. FT-IR spectrum of compound **5a**.

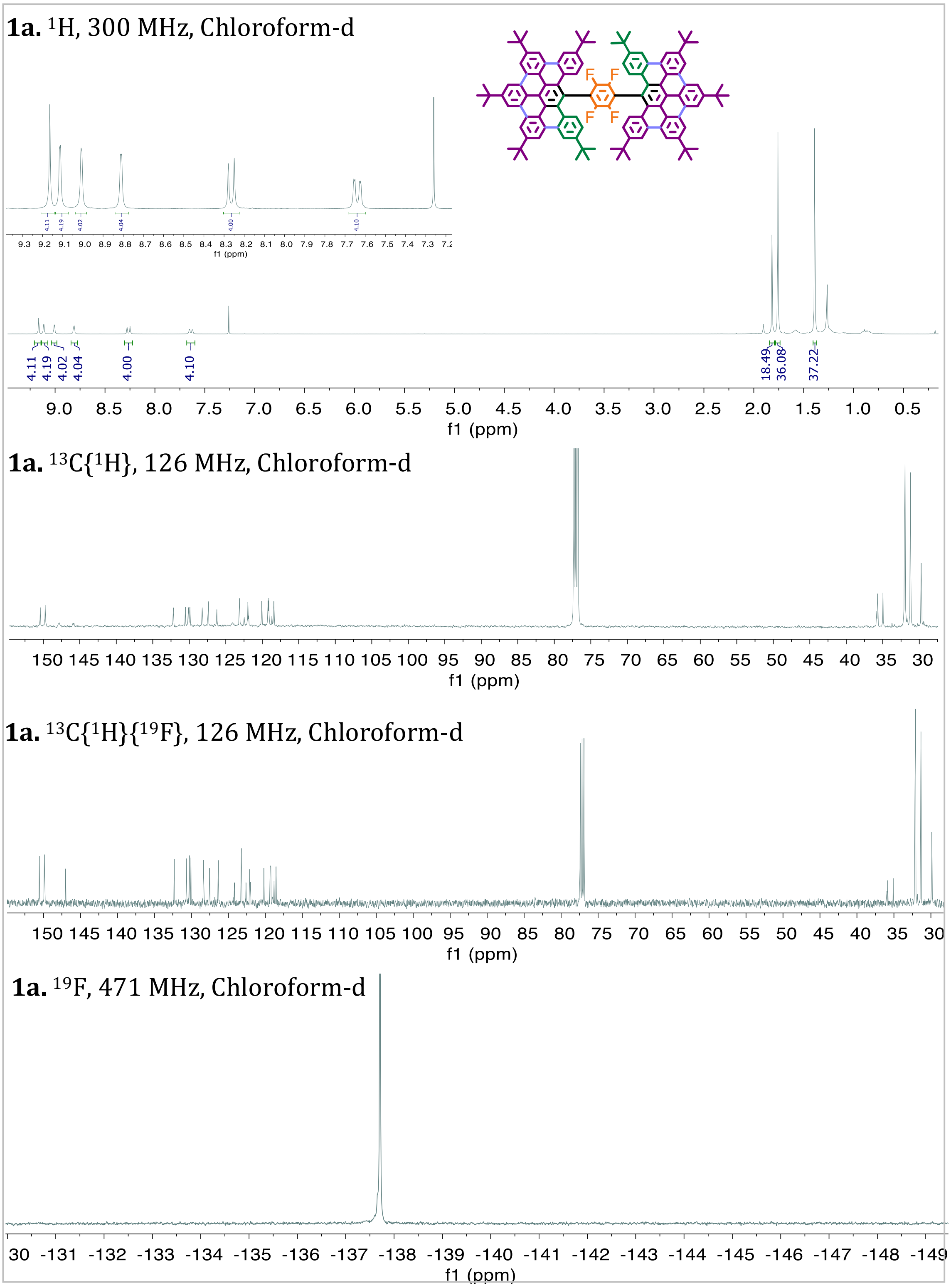

Figure S7. Compound **1a** ^{1}H, ^{13}C{^{1}H}, ^{13}C{^{1}H}{^{19}F} and ^{19}F NMR spectra.

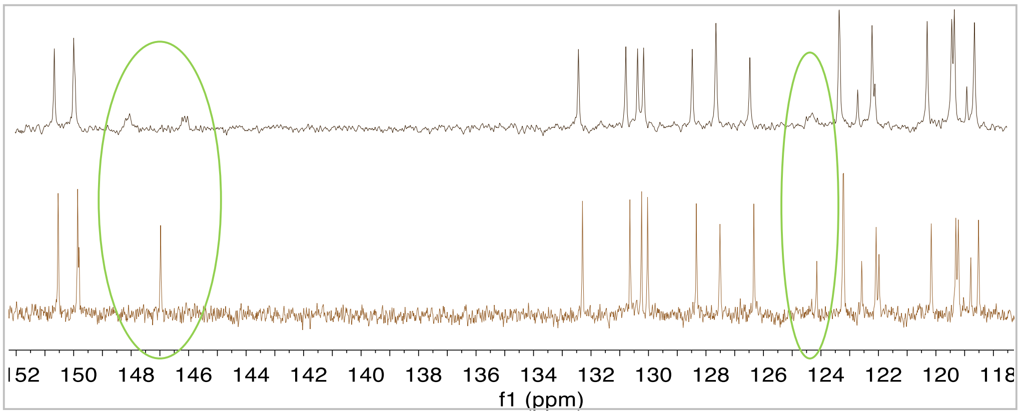

Figure S8. Determination of ^{13}C-^{19}F coupling constant (*J*) for compound **1a** by $^{13}C\{^1H\}$ y $^{13}C\{^1H\}\{^{19}F\}$ spectra comparison.

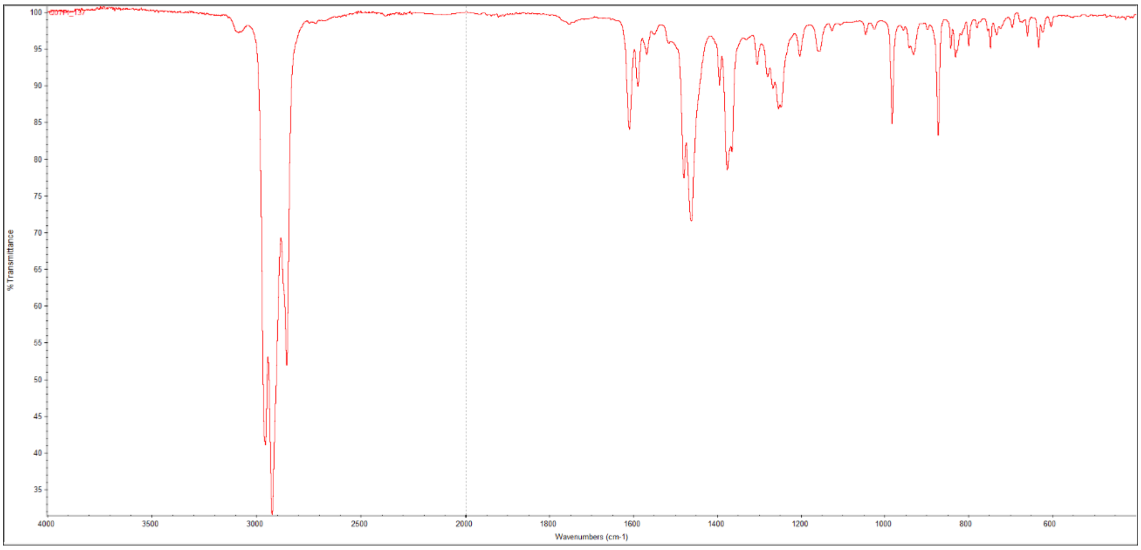

Figure S9. FT-IR spectrum of compound **1a**.

3b. ^{1}H, 300 MHz, Chloroform-d

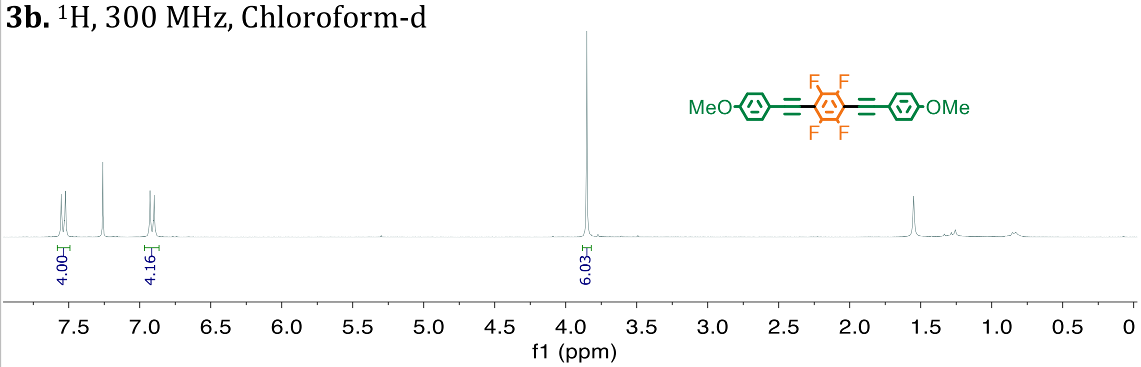

3b. $^{13}C\{^{1}H\}$, 126 MHz, Chloroform-d

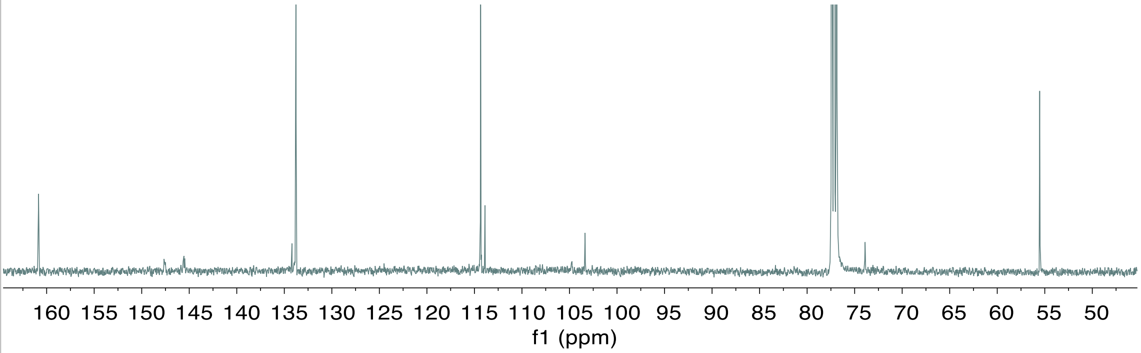

3b. $^{13}C\{^{1}H\}\{^{19}F\}$, 126 MHz, Chloroform-d

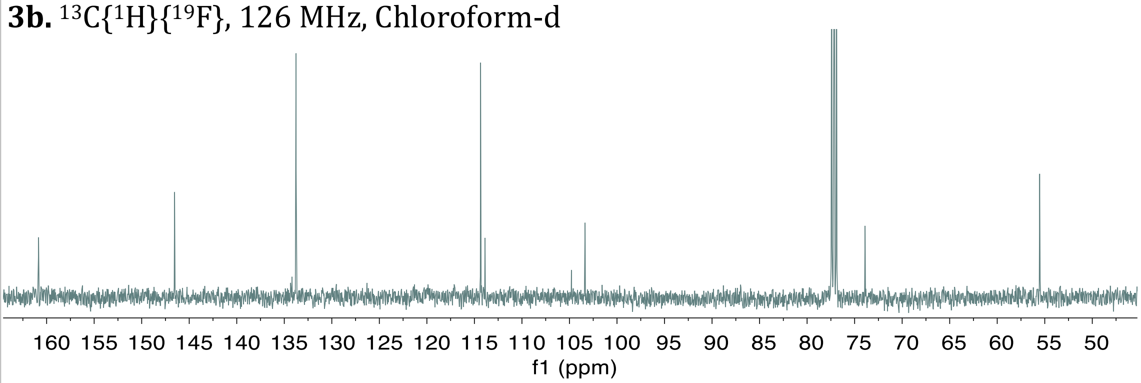

3b. ^{19}F, 471 MHz, Chloroform-d

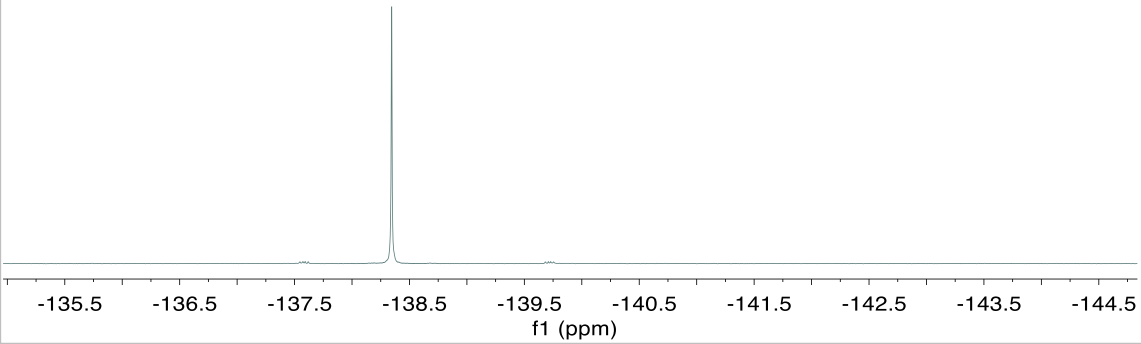

Figure S10. Compound **3b** ^{1}H, $^{13}C\{^{1}H\}$, $^{13}C\{^{1}H\}\{^{19}F\}$ and ^{19}F NMR spectra**.**

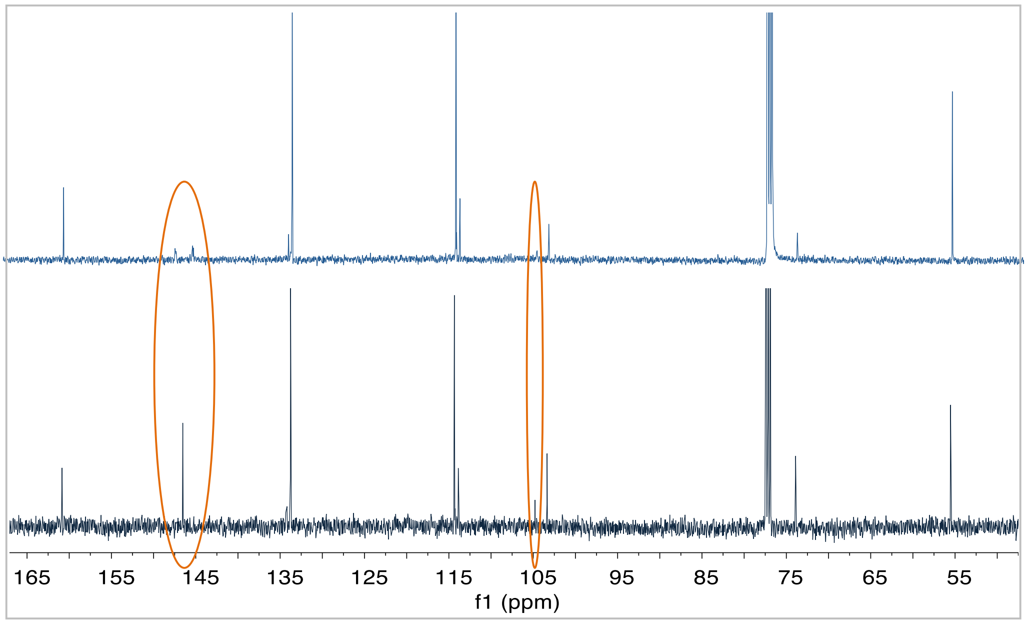

Figure S11. Determination of ^{13}C-^{19}F coupling constant (*J*) for compound **3b** by $^{13}C\{^{1}H\}$ y $^{13}C\{^{1}H\}\{^{19}F\}$ spectra comparison.

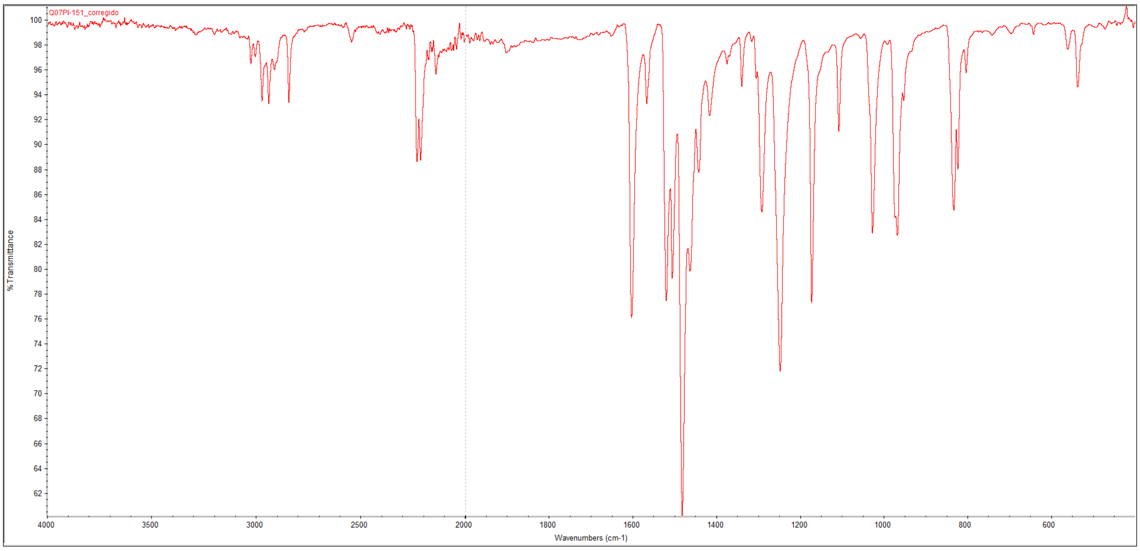

Figure S12. FT-IR spectrum of compound **3b**.

5b. ^{1}H, 500 MHz, Chloroform-d

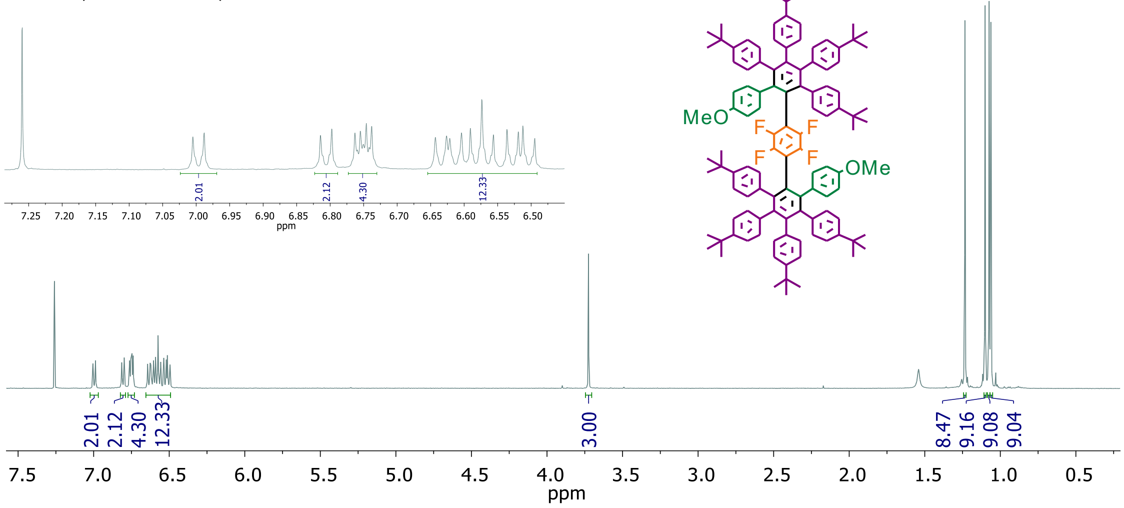

5b. ^{13}C{^{1}H}, 126 MHz, Chloroform-d

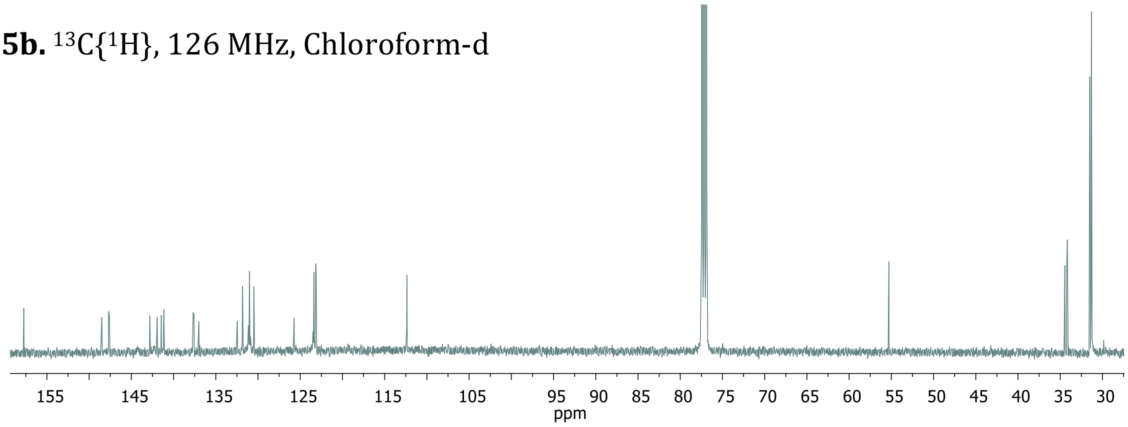

5b. ^{13}C{^{1}H}{^{19}F}, 126 MHz, Chloroform-d

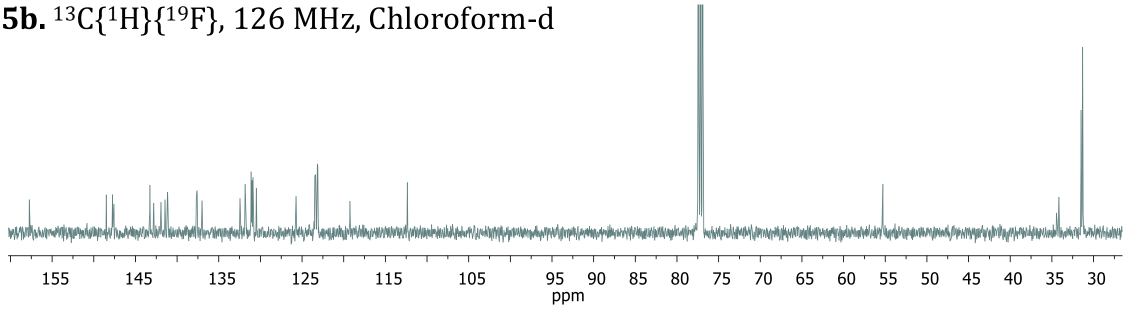

5b. ^{19}F, 471 MHz, Chloroform-d

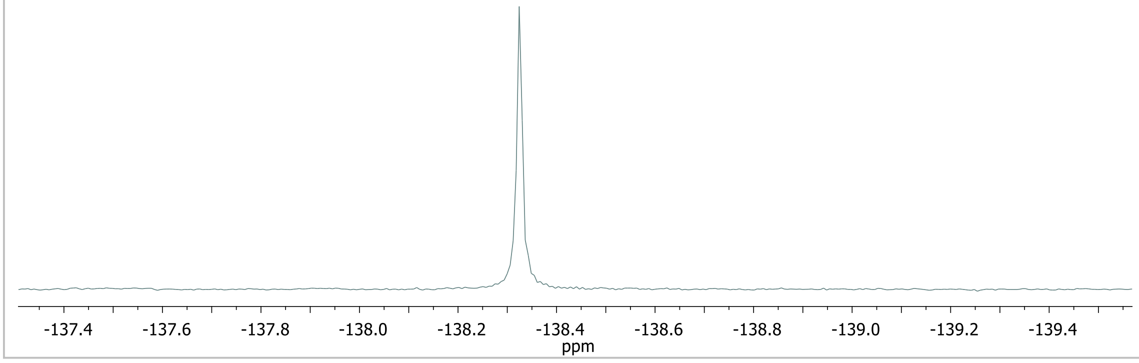

Figure S13. Compound **5b** ^{1}H, ^{13}C{^{1}H}, ^{13}C{^{1}H}{^{19}F} and ^{19}F NMR spectra**.**

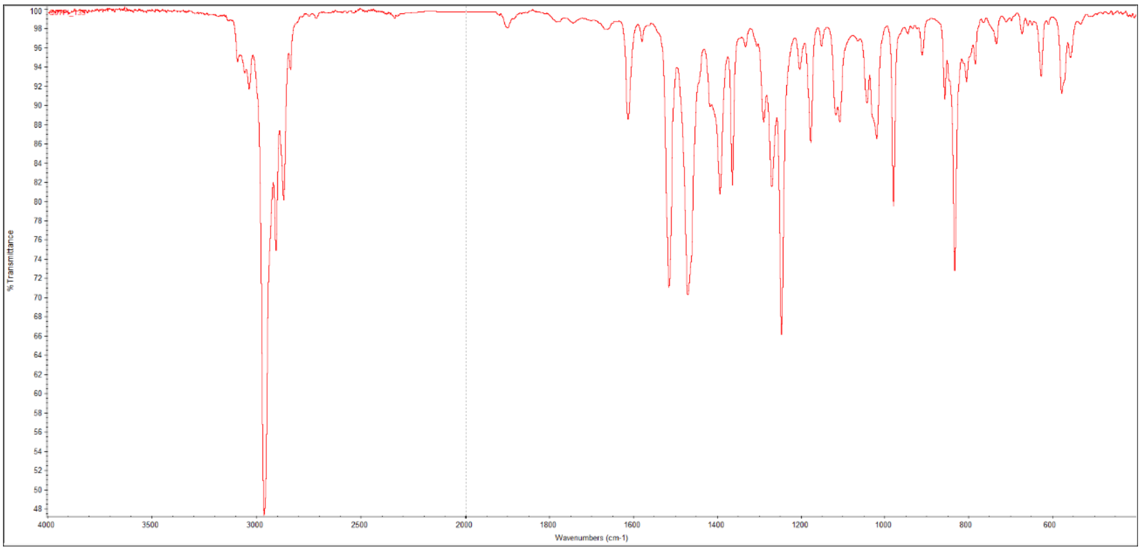

Figure S14. FT-IR spectrum of compound **5b**.

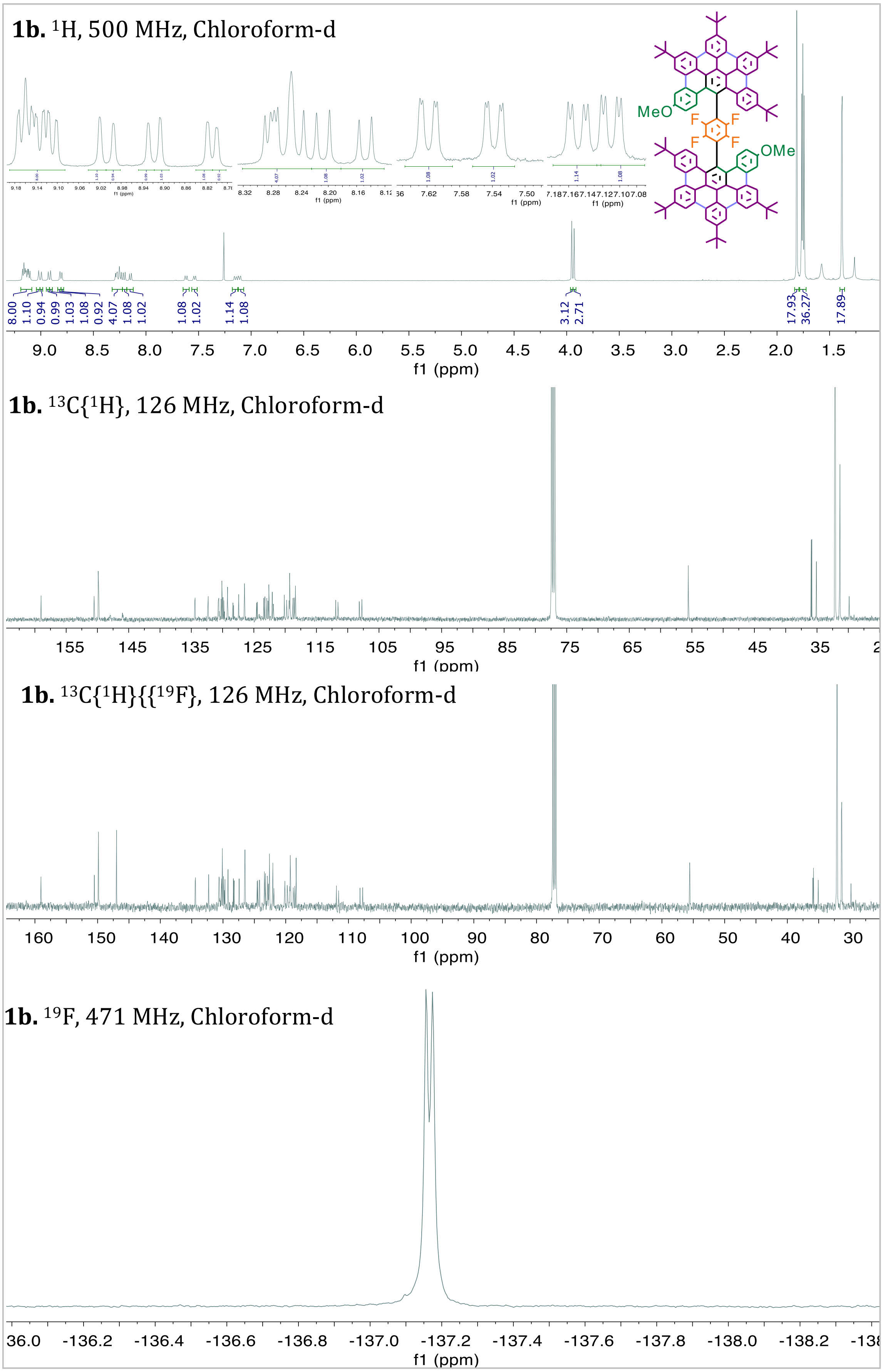

Figure S15. Isomers mixture **1b-*syn*** and **1b-*anti*** ^{1}H, ^{13}C{^{1}H}, ^{13}C{^{1}H}{^{19}F} and ^{19}F NMR spectra**.**

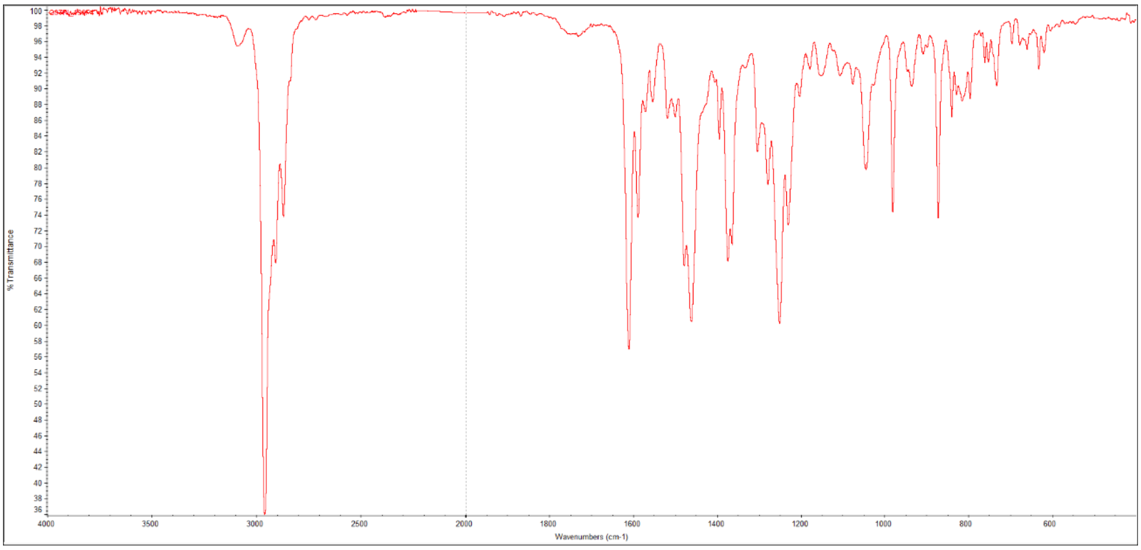

Figure S16. FT-IR spectrum of compound **1b-*syn*** and **1b-*anti***.

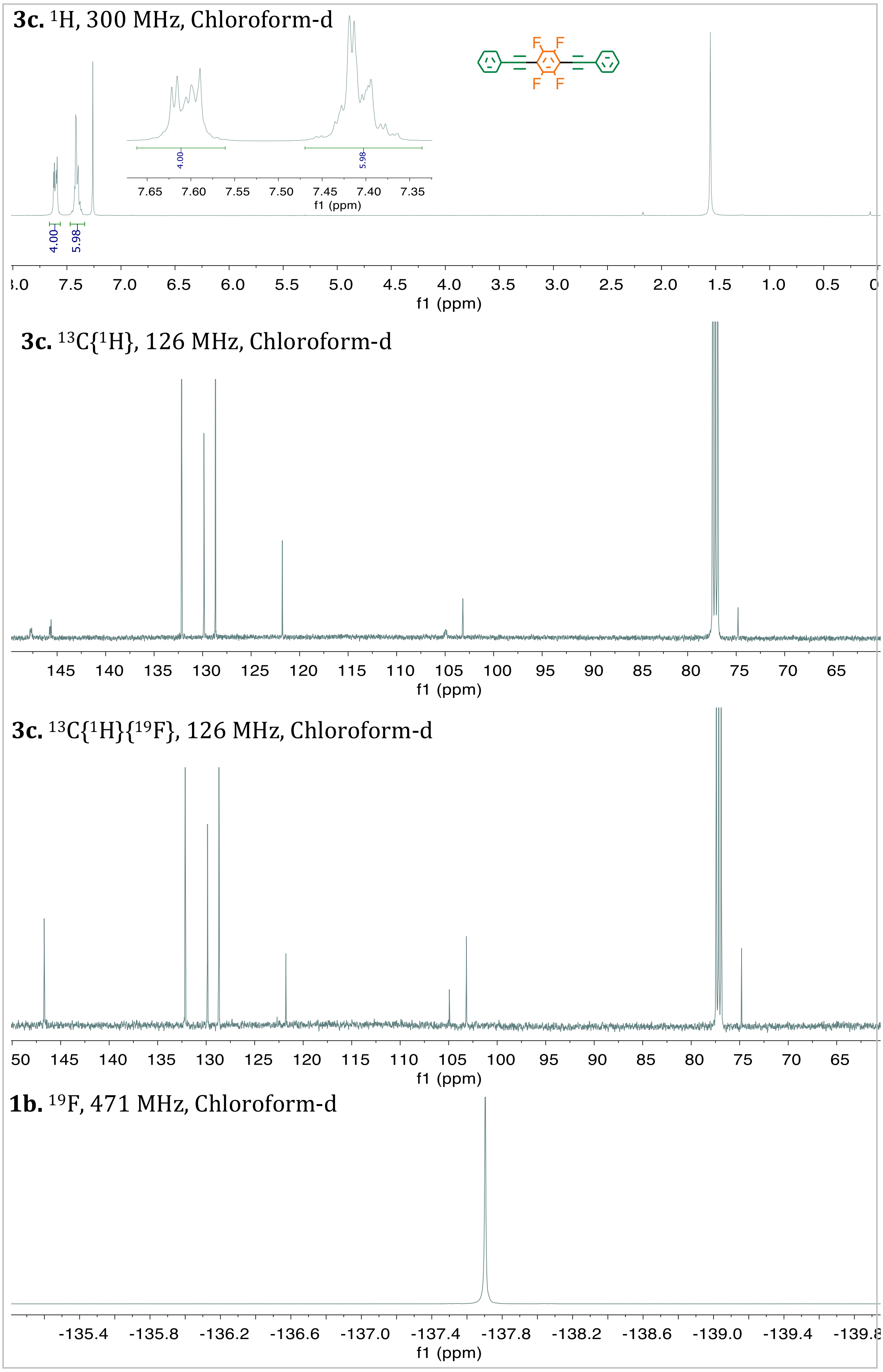

Figure S17. Compound **3c** ^{1}H, $^{13}C\{^{1}H\}$, $^{13}C\{^{1}H\}\{^{19}F\}$ and ^{19}F NMR spectra.

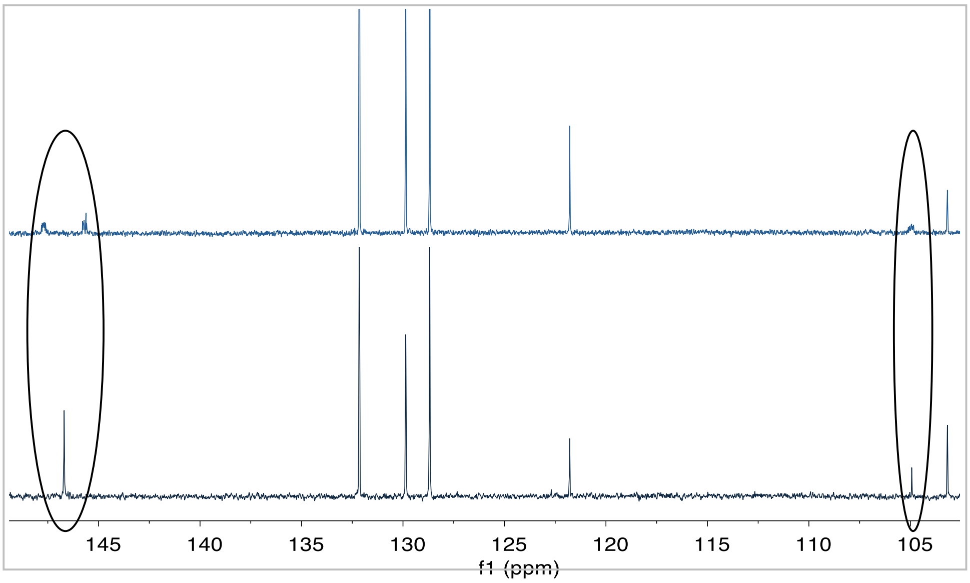

Figure S18. Determination of ^{13}C-^{19}F coupling constant (*J*) for compound **3b** by $^{13}C\{^1H\}$ y $^{13}C\{^1H\}\{^{19}F\}$ spectra comparison.

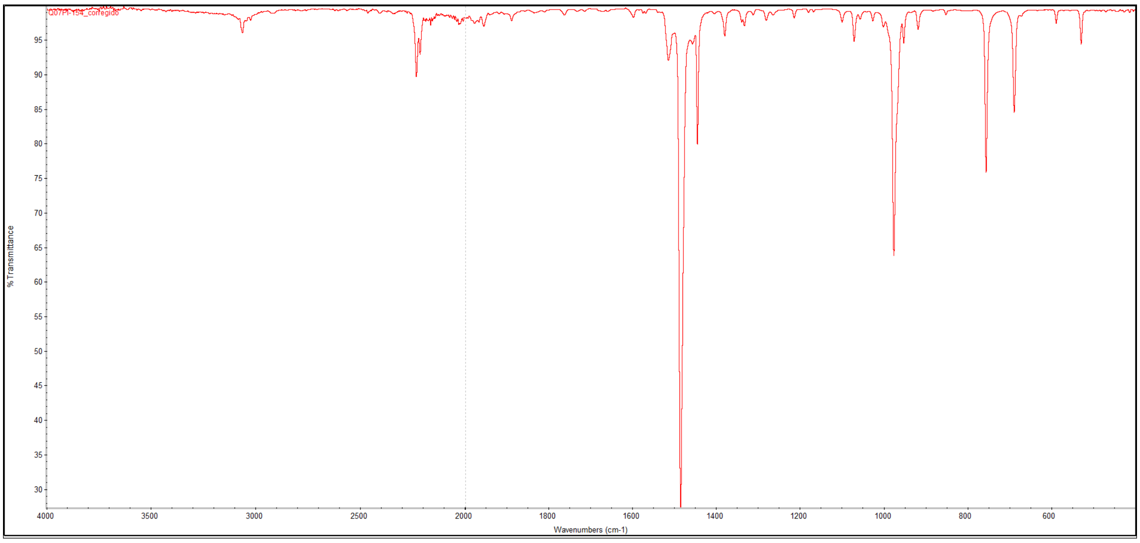

Figure S19. FT-IR spectrum of compound **3c**.

5c. ^{1}H, 500 MHz, Chloroform-d

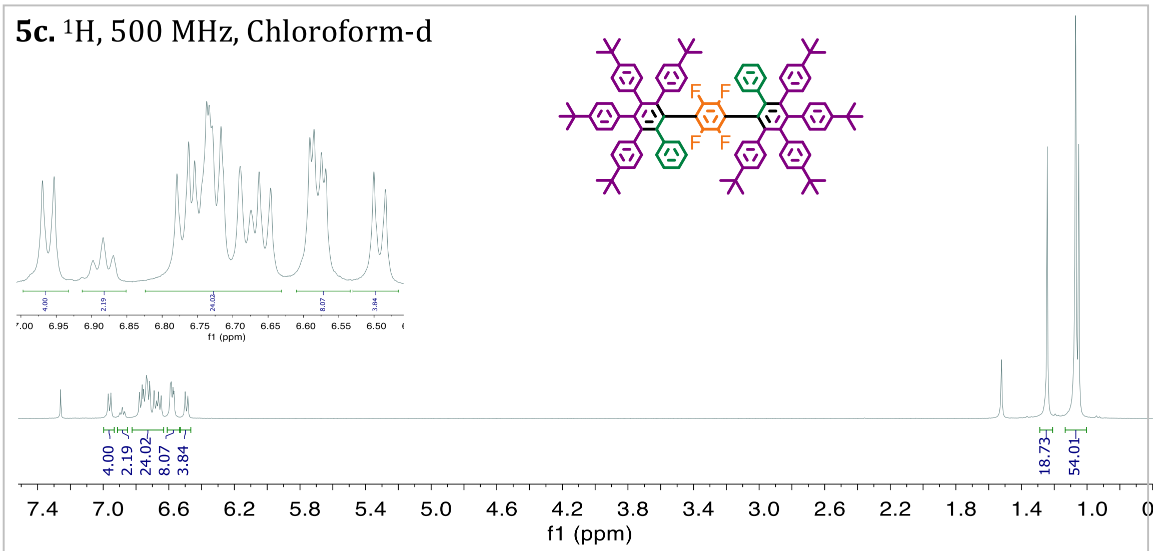

5c. $^{13}C\{^{1}H\}$, 126 MHz, Chloroform-d

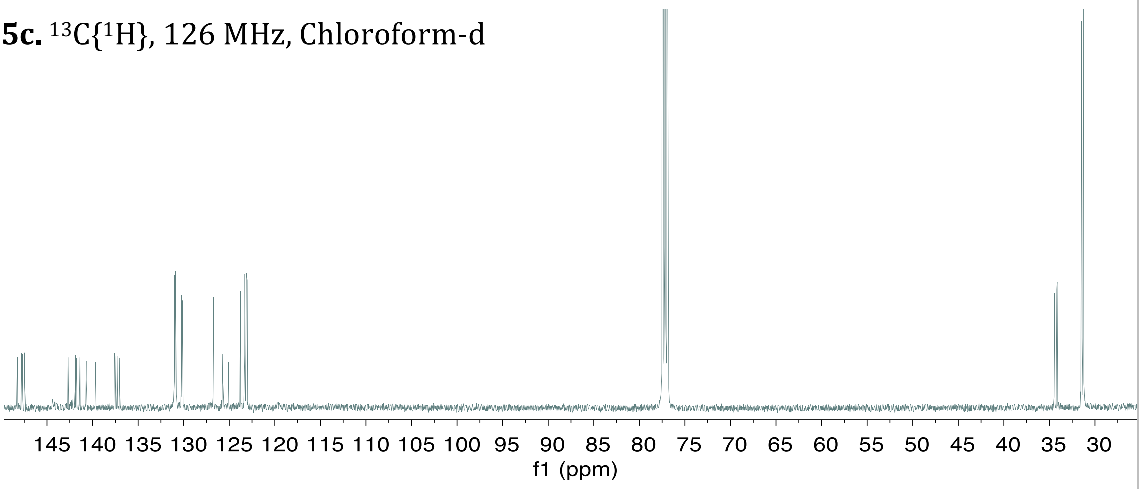

5c. $^{13}C\{^{1}H\}\{^{19}F\}$, 126 MHz, Chloroform-d

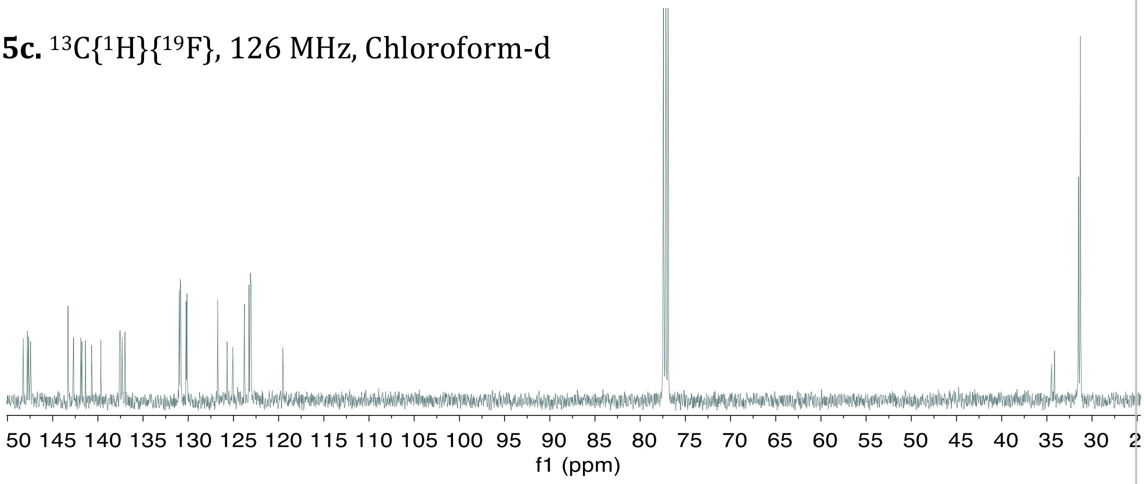

5c. ^{19}F, 282 MHz, Chloroform-d

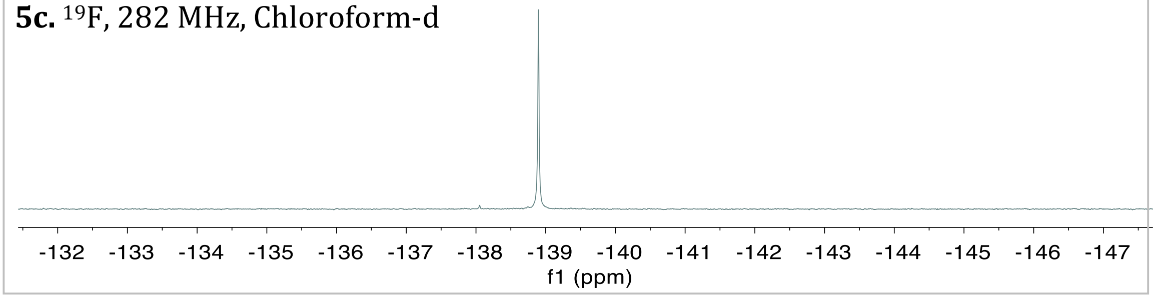

Figure S20. Isomer mixture **5c** ^{1}H, $^{13}C\{^{1}H\}$, $^{13}C\{^{1}H\}\{^{19}F\}$ and ^{19}F NMR spectra**.**

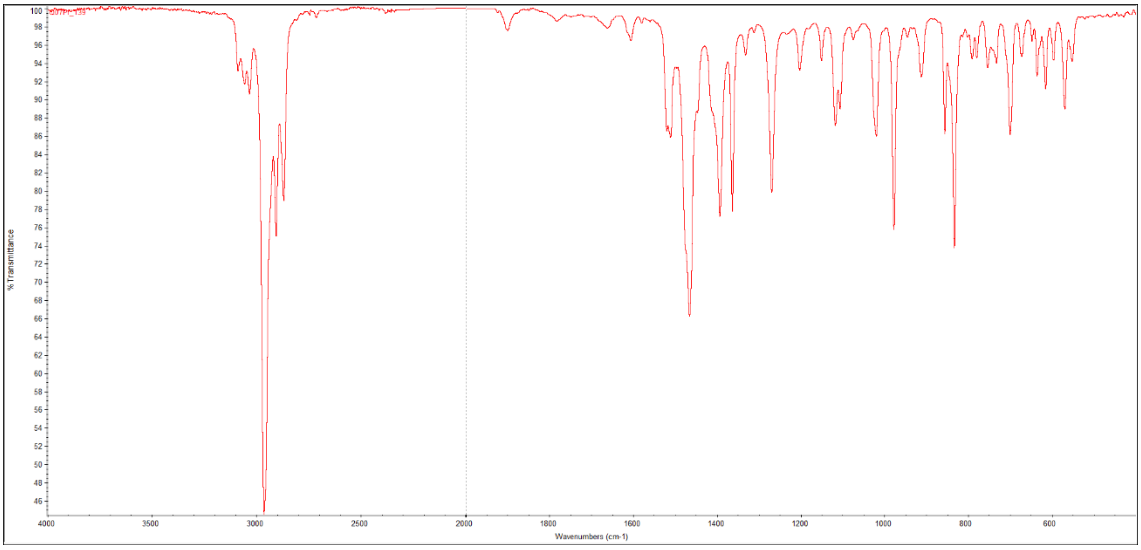

Figure S21. FT-IR spectrum of **5c**.

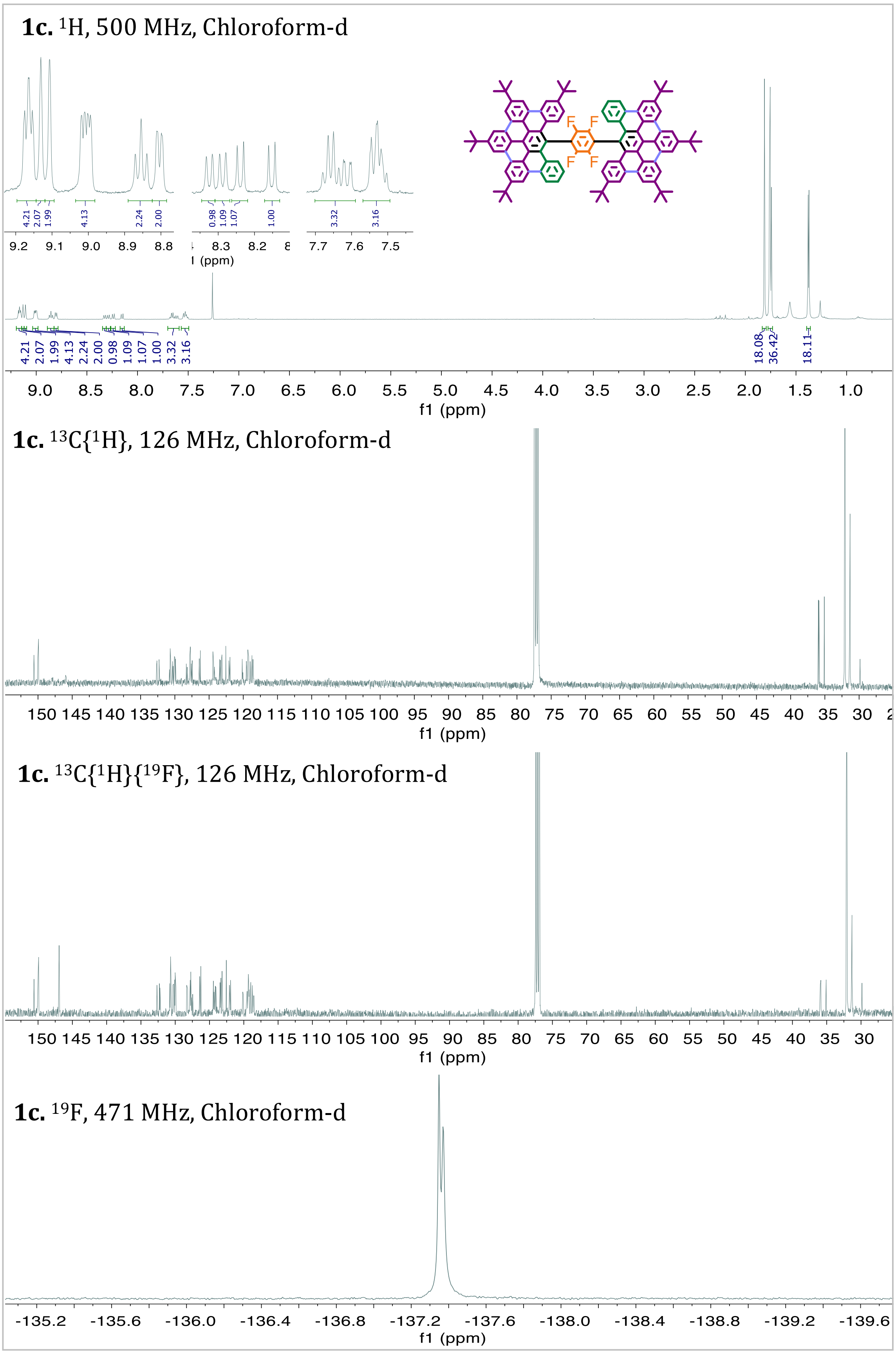

Figure S22. Isomers mixture **1c-*syn*** and **1c-*anti*** ^{1}H, $^{13}C\{^{1}H\}$, $^{13}C\{^{1}H\}\{^{19}F\}$ and ^{19}F NMR spectra**.**

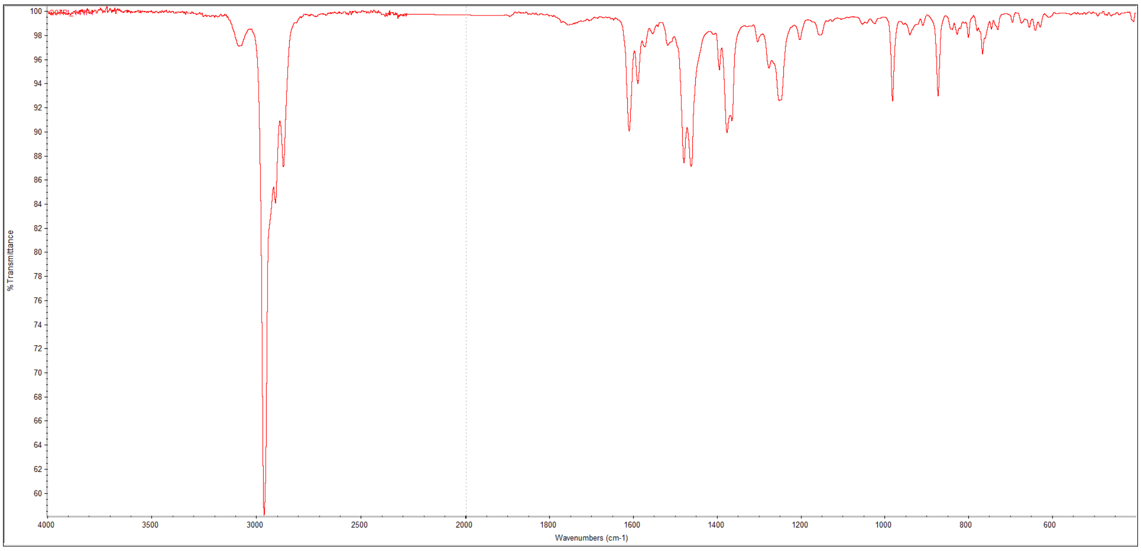

Figure S23. FT-IR spectrum of compound **1c-*syn*** and **1c-*anti***.

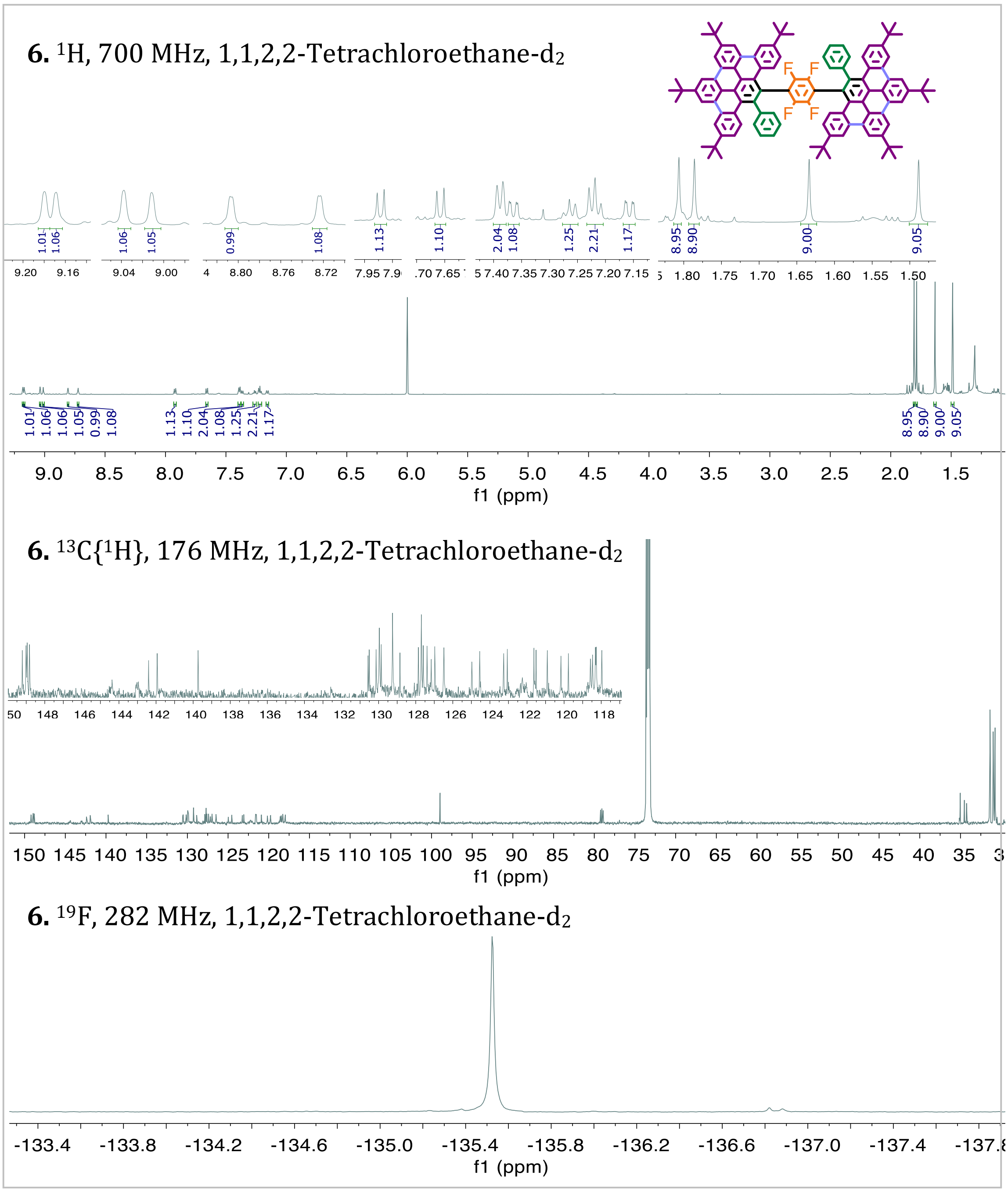

Figure S24. Compound **6** ^{1}H, $^{13}C\{^{1}H\}$ and ^{19}F NMR spectra**.**

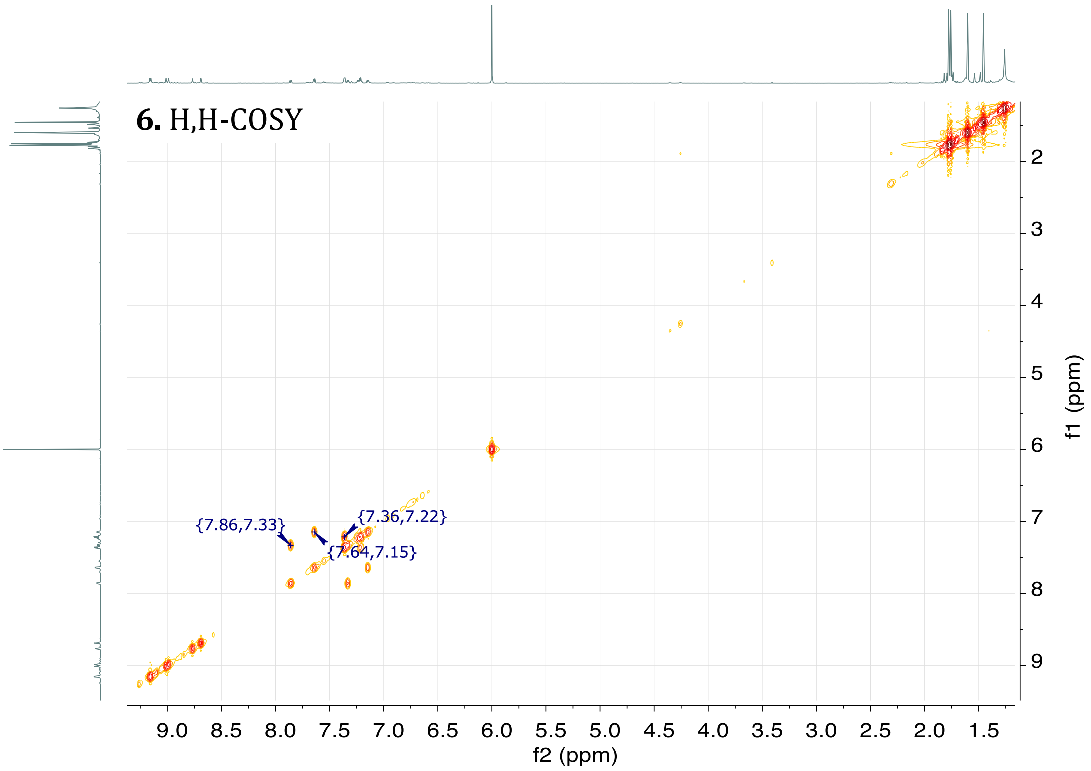
6. H,H-COSY
{7.86,7.33}
{7.36,7.22}
{7.64,7.15}
f2 (ppm)
f1 (ppm)

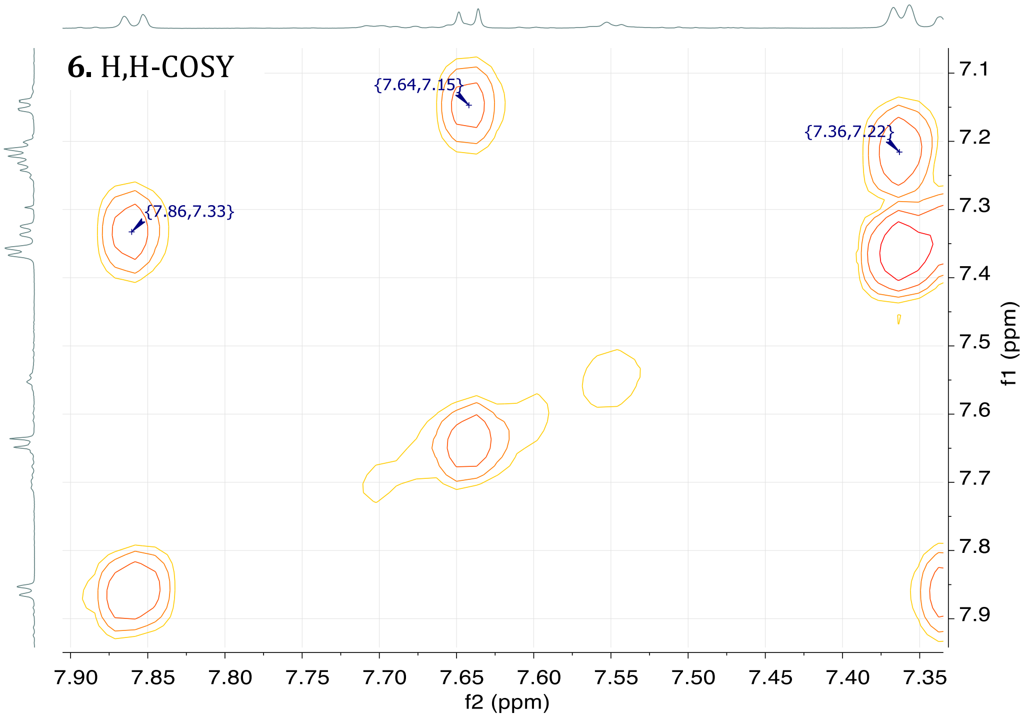
6. H,H-COSY
{7.64,7.15}
{7.36,7.22}
{7.86,7.33}
f2 (ppm)
f1 (ppm)

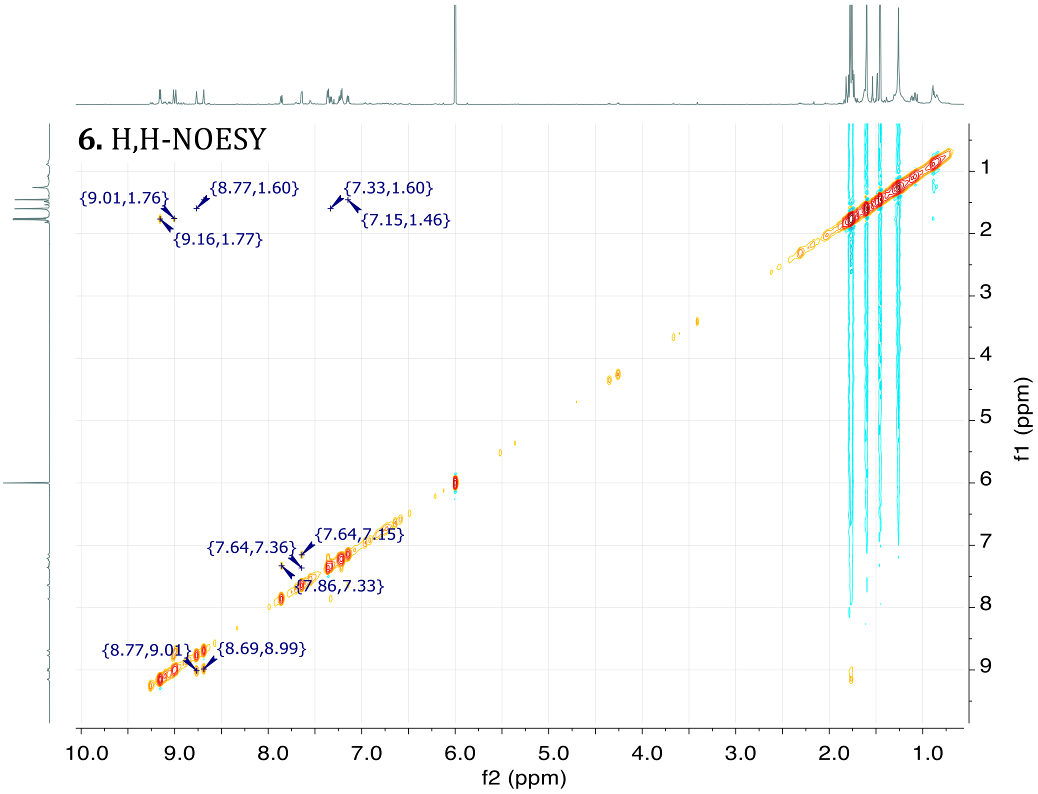
6. H,H-NOESY
{9.01,1.76}
{8.77,1.60}
{7.33,1.60}
{7.15,1.46}
{9.16,1.77}
{7.64,7.36}
{7.64,7.15}
{7.86,7.33}
{8.77,9.01}
{8.69,8.99}
f1 (ppm)
f2 (ppm)

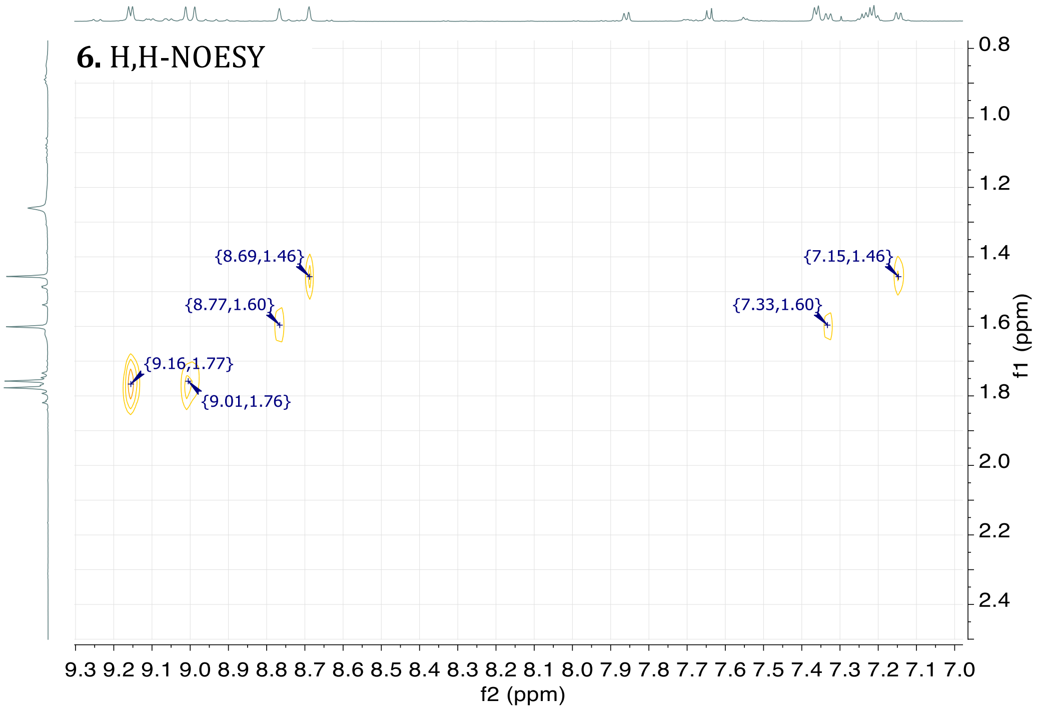
6. H,H-NOESY
{8.69,1.46}
{8.77,1.60}
{9.16,1.77}
{9.01,1.76}
{7.15,1.46}
{7.33,1.60}
f1 (ppm)
f2 (ppm)

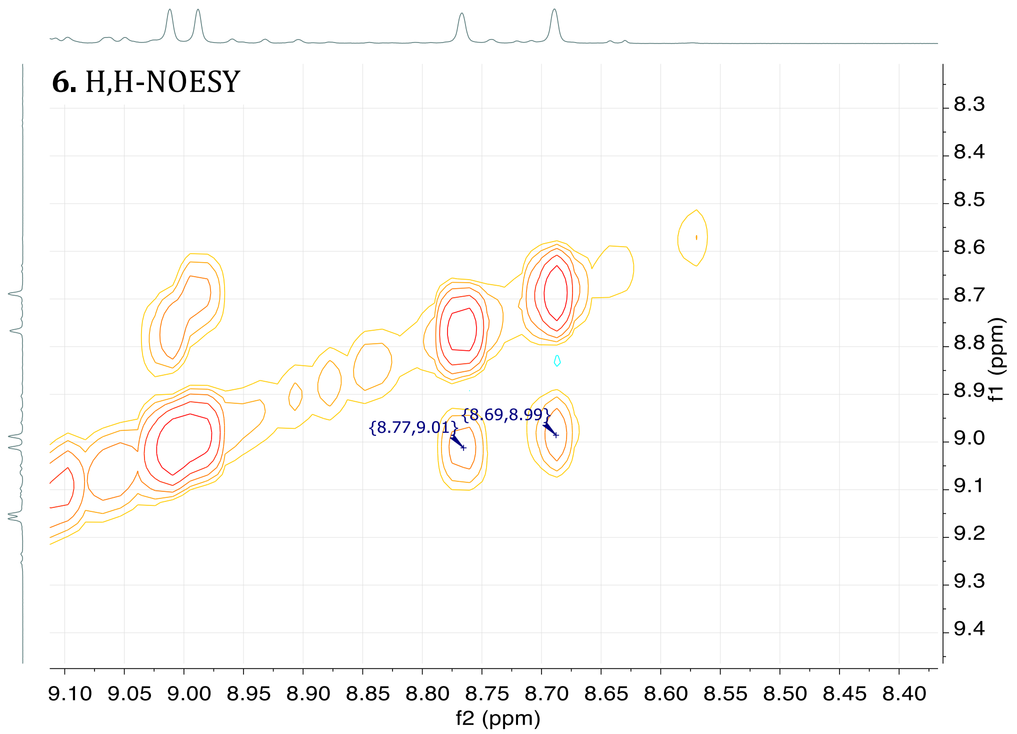

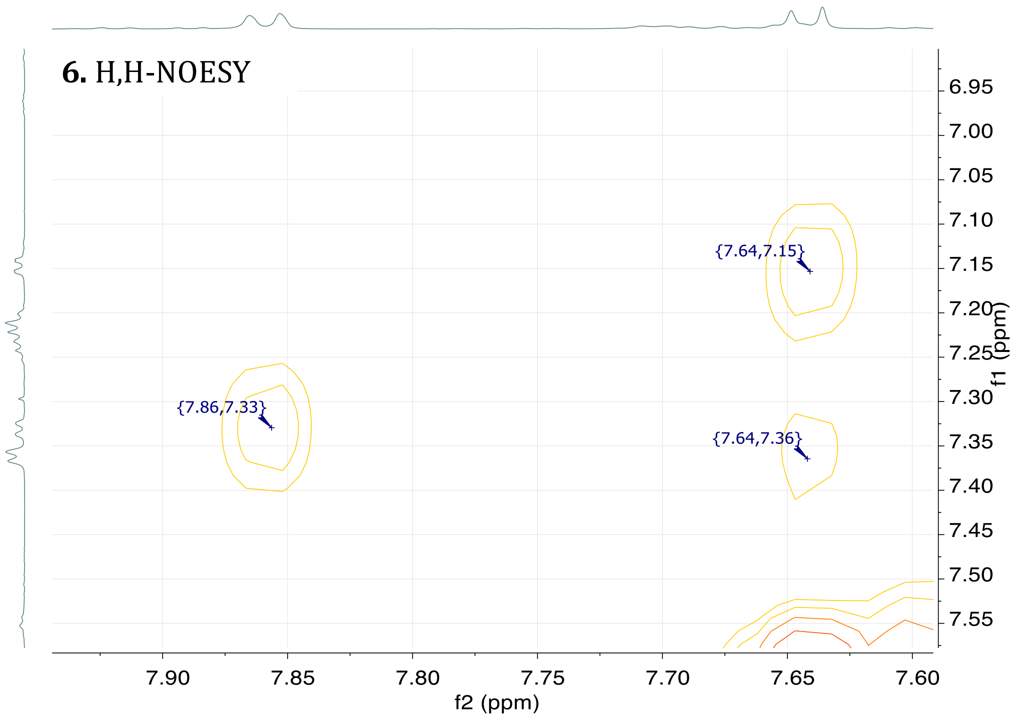

Figure S25. Compound **6** H,H-COSY and H,H-NOESY NMR spectra**.**

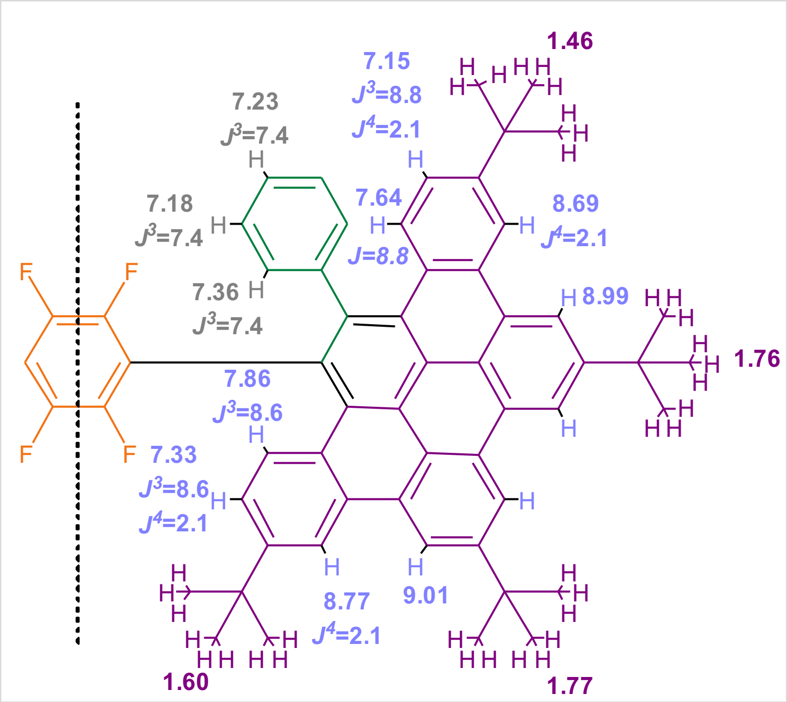

Figure S26. Compound **6** ^{1}H NMR assignment according to previous data**.**

4. Single Crystal X-ray structure determination

Single crystals suitable for X-ray diffraction analysis were obtained for compound **1a**. The molecule presents chirality, although the crystals obtained from the racemic mixture are racemic. These crystals are not stable out of the mother liquor and quickly lose crystallinity (see figure S27).

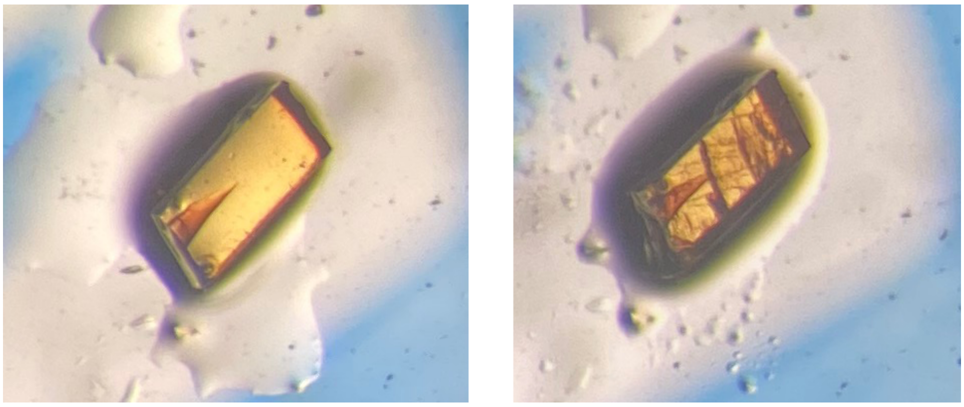

Figure S27. Left: Fragment of a crystal of compound **1a**. Right: Loss of crystallinity after 1 minute in ambient conditions.

The structure refinement presents a high wR_2 value due to the high degree of disorder that many of the atoms in the asymmetric unit display. The solvent molecules (dichloroethane and water) in the interstices are extremely disordered, as well as the peripheral *t*-Bu substituents. The coordinates of the atoms in the *t*-Bu fragments had to be refined with the aid of geometrical restraints, and for some of the terminal methyl groups (C60-C62, C104-C106, C108-C110, C112-C114, C116-C118) these positions only account for 50% of the corresponding electron density.

Several single crystal X-ray diffraction experiments were performed to ensure that the molecular model could not be improved prior. In particular, a second good quality single crystal was selected, and a new full data collection was performed, together with the structure solution and model refinement. However, the R-factors in the resulting model could not be improved, as they are a consequence of the large portions of disordered atoms in the crystal. Interestingly, the new structure solution allowed us to confirm that the high degree of disorder in the crystals of **1a** is due to both the flexibility of the molecules themselves and the random positions of the solvents in the large interstitial voids (see figure S28).

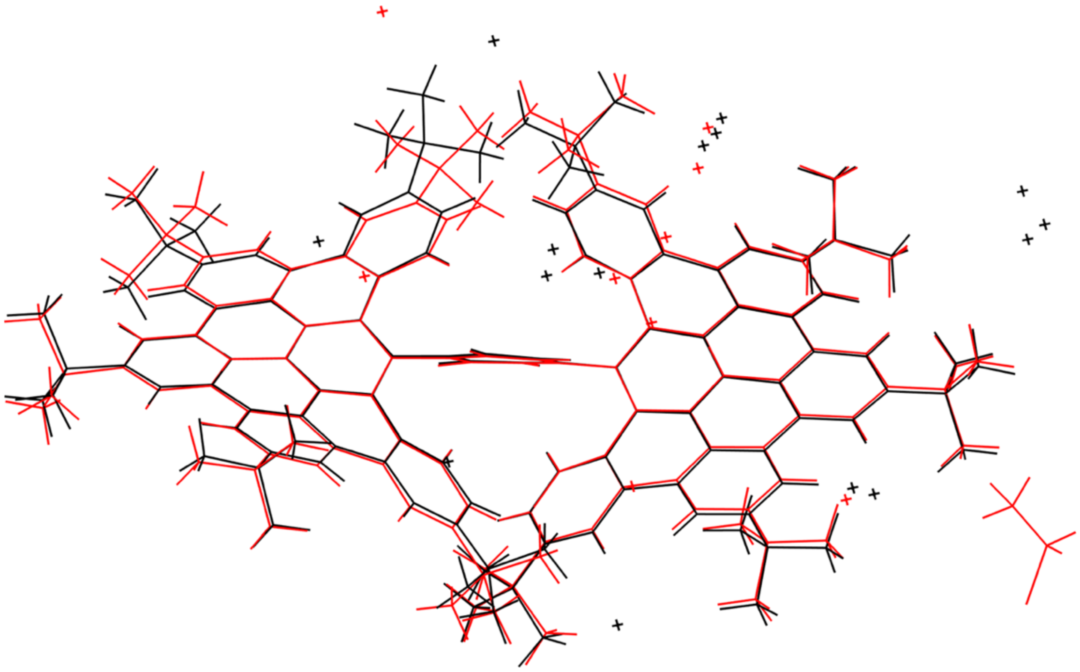

Figure S28. Graphical overlay of the asymmetric units from the electron density maps of two different single crystals of compound **1a**. In red, the structure with the best quality indicators (submitted with the manuscript).

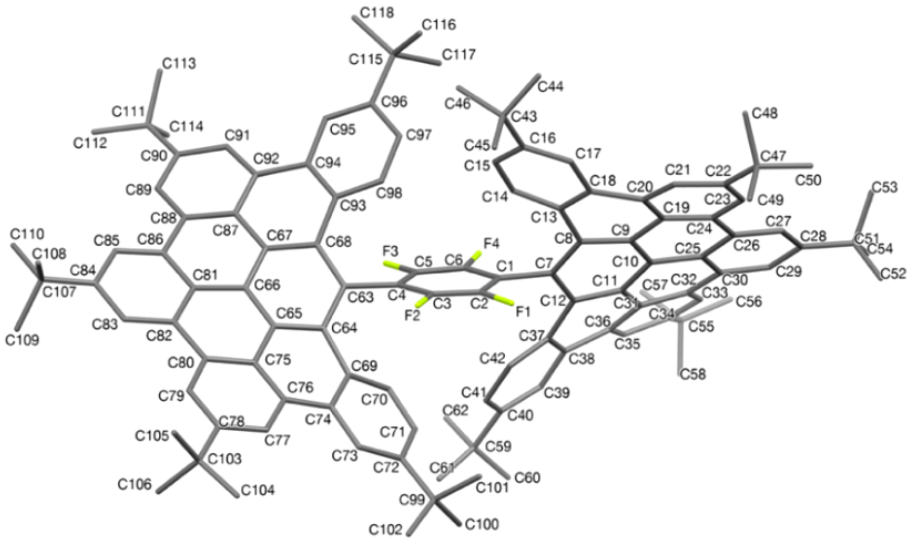

Figure S29. Labelled asymmetric unit of compound **1a** (solvent molecules have been omitted).

Structural details of 1a (CCDC 2070237)

Table S1. Sample and crystal data for **1a**.

**Chemical formula**	$(C_{118}H_{114}F_4)\cdot(C_2H_2Cl_2)_{0.5}(H_2O)_{4.5}$
**Formula weight**	1737.72
**Temperature**	200(2) K
**Wavelength**	0.71073 Å

**Crystal size**	0.105 x 0.221 x 0.261 mm	
**Crystal habit**	clear orange needle	
**Crystal system**	monoclinic	
**Space group**	*C*2/*c*	
**Unit cell dimensions**	*a* = 35.348(2) Å	α = 90°
	b = 31.054(1) Å	β = 114.553(2)°
	c = 22.447(1) Å	γ = 90°
**Volume**	22412(2) $Å^3$	
**Z**	8	

Table S2. Data collection and structure refinement for **1a**.

**Theta range for data collection**	1.14 to 23.96°	
**Index ranges**	-33<=h<=40, -34<=k<=28, -25<=l<=16	
**Reflections collected**	43262	
**Independent reflections**	16808 [R(int) = 0.0678]	
**Coverage of independent reflections**	96%	
**Absorption correction**	multi-scan	
**Max. and min. transmission**	0.9910 and 0.9780	
**Structure solution technique**	direct methods	
**Structure solution program**	SHELXS-97 (Sheldrick 2008)	
**Refinement method**	Full-matrix least-squares on F^2	
**Refinement program**	SHELXL-2014/7 (Sheldrick, 2014)	
**Function minimized**	Σ w(F_o^2 - F_c^2)^2	
**Data / restraints / parameters**	16808/33/1057	
**Goodness-of-fit on F^2**	1.123	
**Final R indices**	8046 data; I>2σ(I)	R_1 = 0.1564, wR_2 = 0.4257
	all data	R_1 = 0.2562 wR_2 = 0.5056
**Weighting scheme**	w=1/[σ^2(F_o^2)+(0.3200P)^2+19.700P]<br>where P=(F_o^2+2F_c^2)/3	

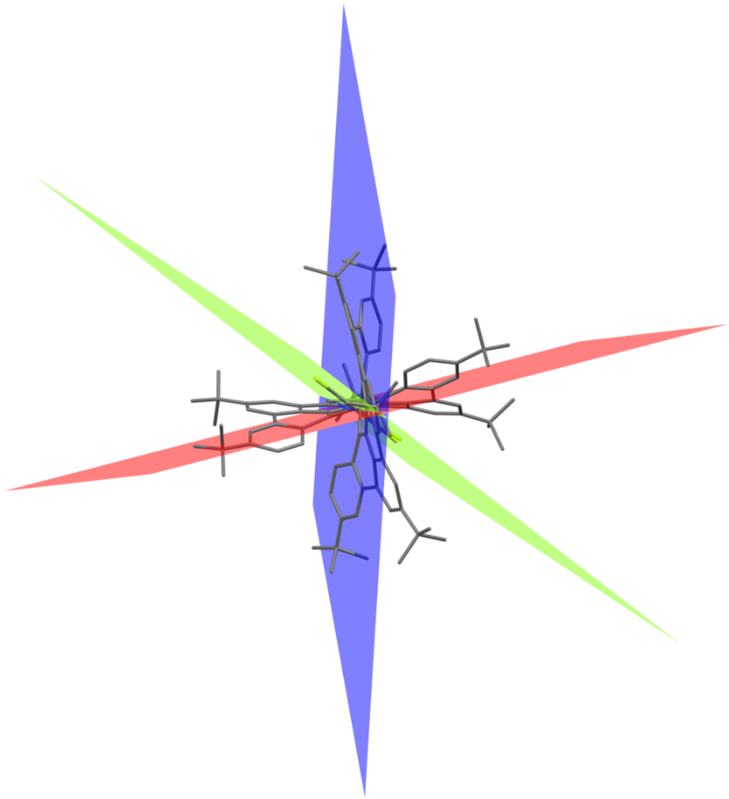

Figure S30. Planes calculated for the three central rings of the molecule: in green, C1-C6; in blue C7-C12 and in red, C63-C68.

Table S3. C-H···π interactions in the crystal structure of **1a.** The most relevant ones (according to the values mentioned by M. Nishio in *Phys. Chem. Chem. Phys*, 2011, 13, 13873) are marked in bold itallics.

**C-H···· ℭentroid**	**Distance (Å)**
C49-H49B· ···ℭ (C35-C36)	2.772
C109-H09C···· ℭ (C13-C14)	***2.652***
C109-H09C···· ℭ (C13-C18)	***2.624***
C109-H09C···· ℭ (C18-C20)	2.994
C110-H10B···· ℭ (C18-C20)	2.930

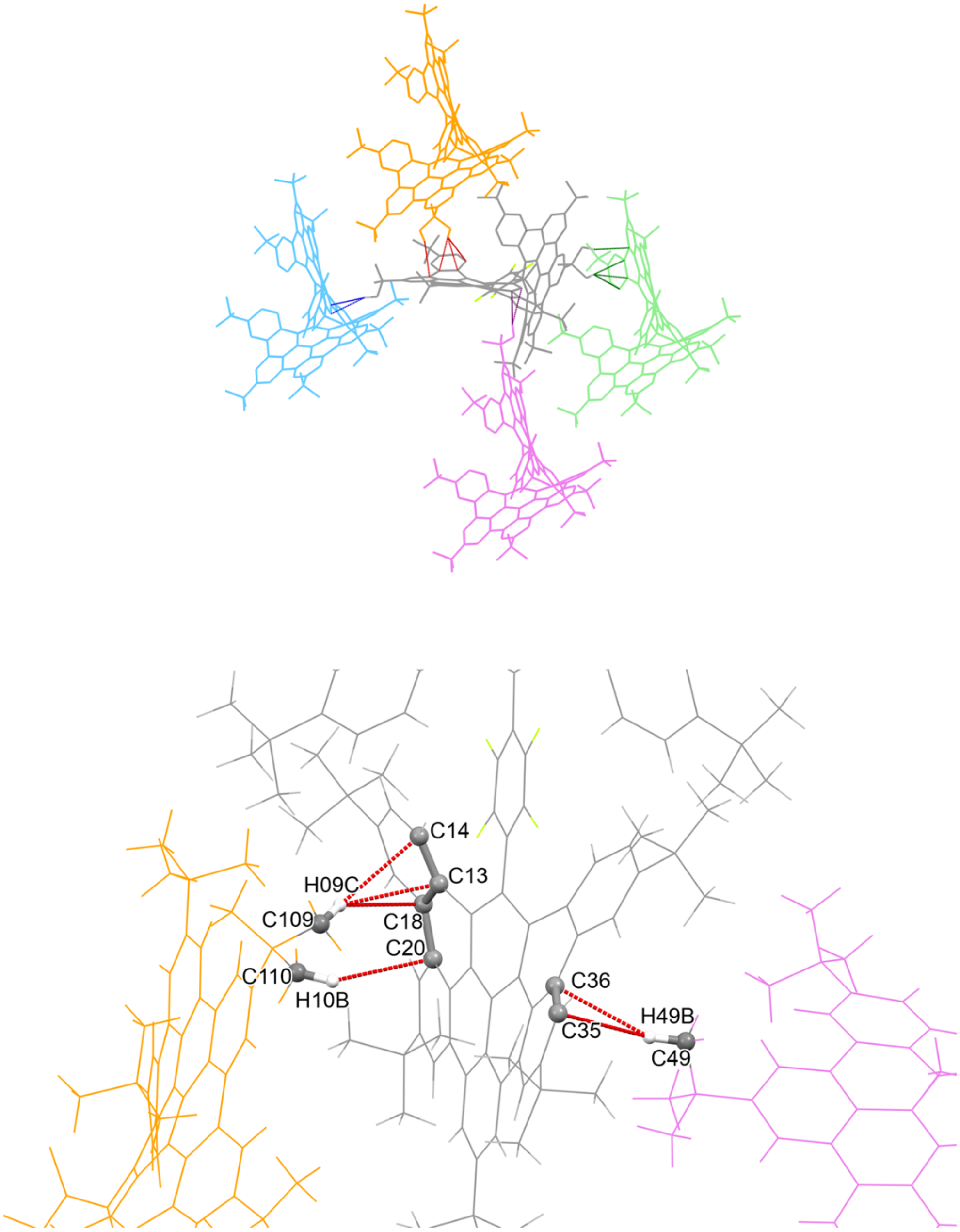

Figure S31. Top: supramolecular C-H···π bonds from a nanographene **1a** molecule (in grey) with its four neighbours (in Orange, magenta blue and green). Bottom: detail of the two different zones of interaction with the atoms involved labelled.

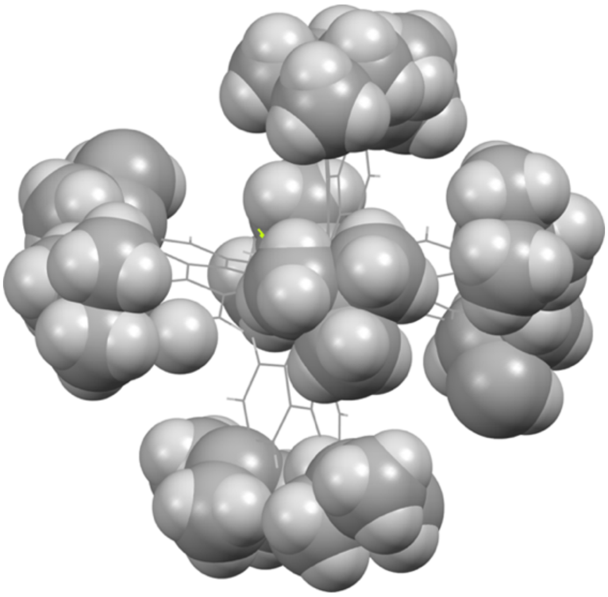

Figure S32. View of a molecule of nanographene **1a** where the t-Bu substituents have been depicted in *spacefill* mode to better visualize their distribution and steric hindrance.

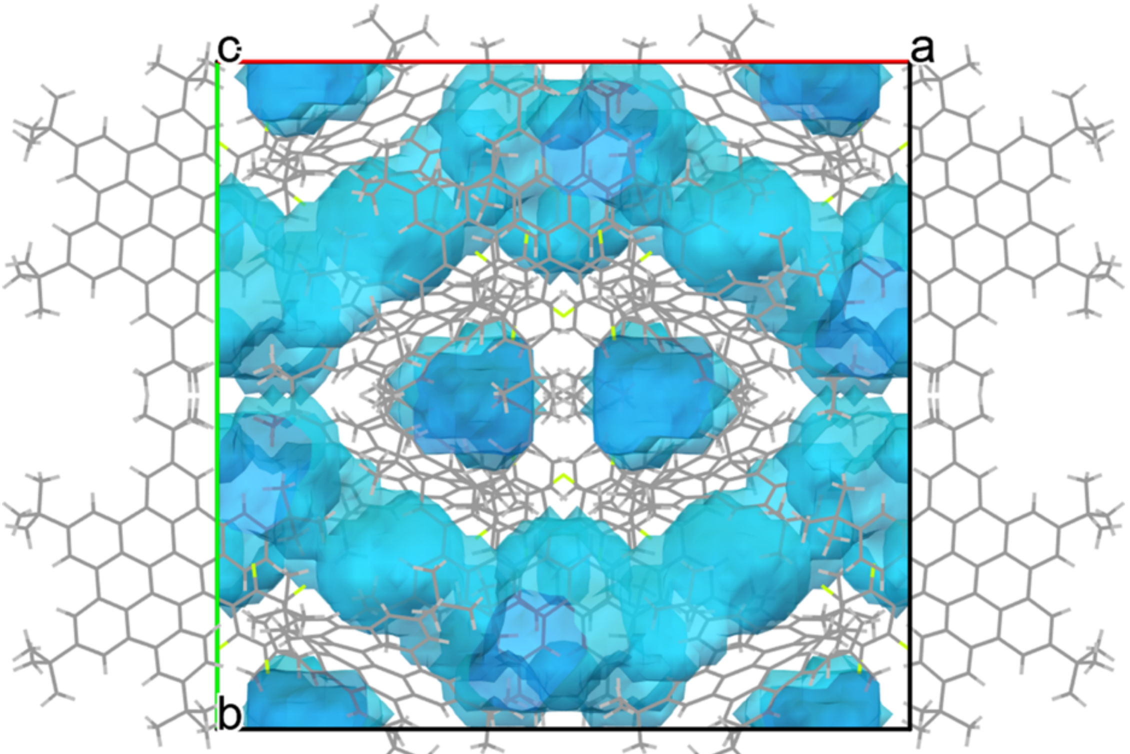

Figure S33. View of the large pores (in blue) in the unit cell of the structure of **1a** that occupy 22.4% of the cell volume, calculated with a probe radius of 1.2 Å and depicted with a grid spacing of 0.7 Å.

5. Isomerization barrier determination

To a 100 mL dry flask, under argon atmosphere and provided with a magnetic stir bar, **5c** (0.01 mmol, 20 mg, 1 equiv.), 40 mL of DCM and DDQ (0.58 mmol, 132 mg, 44 equiv.) were added. The reaction mixture was chilled at -65 °C and was stirred for 5 minutes. Then, under Ar bubbling, TfOH was added dropwise (5.45 mmol, 820 mg, 412 equiv.) and the reaction was carried out at -65 °C maintaining the Ar bubbling for 3 hours. After that time, a $HNaCO_3$ saturated solution was added to the reaction flask and the crude was extracted with DCM and washed with a $HNaCO_3$ saturated solution (20 mL) twice and brine (20 mL) twice. The organic phase was dried with $MgSO_4$ and filtered through silica gel using DCM as eluent. The solvent was removed under reduced pressure affording a 70:30 **1c-*anti*/1c-*syn*** isomeric mixture with non complete conversion as a yellow solid.

The isomerization barrier was studied by ^{1}H NMR in a 700 MHz Bruker AVIII at 320 K, recording spectra every 10 minutes approximately. The monitored signals were the doublets at 8.31 (*anti*), 8.27 (*syn*), 8.22 (*syn*) and 8.14 (*anti*) ppm (assigned by 2D-NMR) in 1,1,2,2-tetrachloroethane-d_2 with a 70 (*anti*):30(*syn*) starting relation between the two isomers, **Figure S34**. As it is shown in **Figure S37**, doublets at 8.31 and 8.14 ppm decrease as doublets at 8.27 and 8.22 increased with time until the equilibrium is reached and the two isomers were in 1:1 relation.

The assignation of *anti* and *syn* isomers was deduced from 2D of 1:1 *anti:syn* mixture **1c** in a 700MHz Bruker AVIII (**Figure S35**).

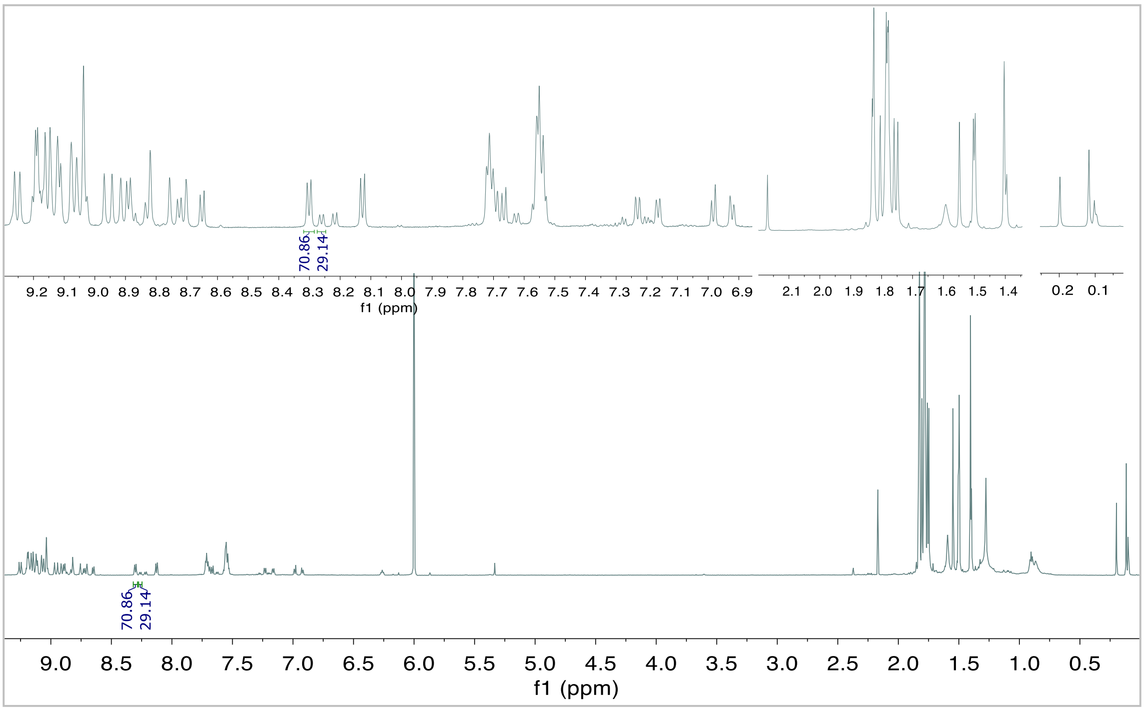

Figure S34. Starting ^{1}H NMR spectra showing the relation between isomers.

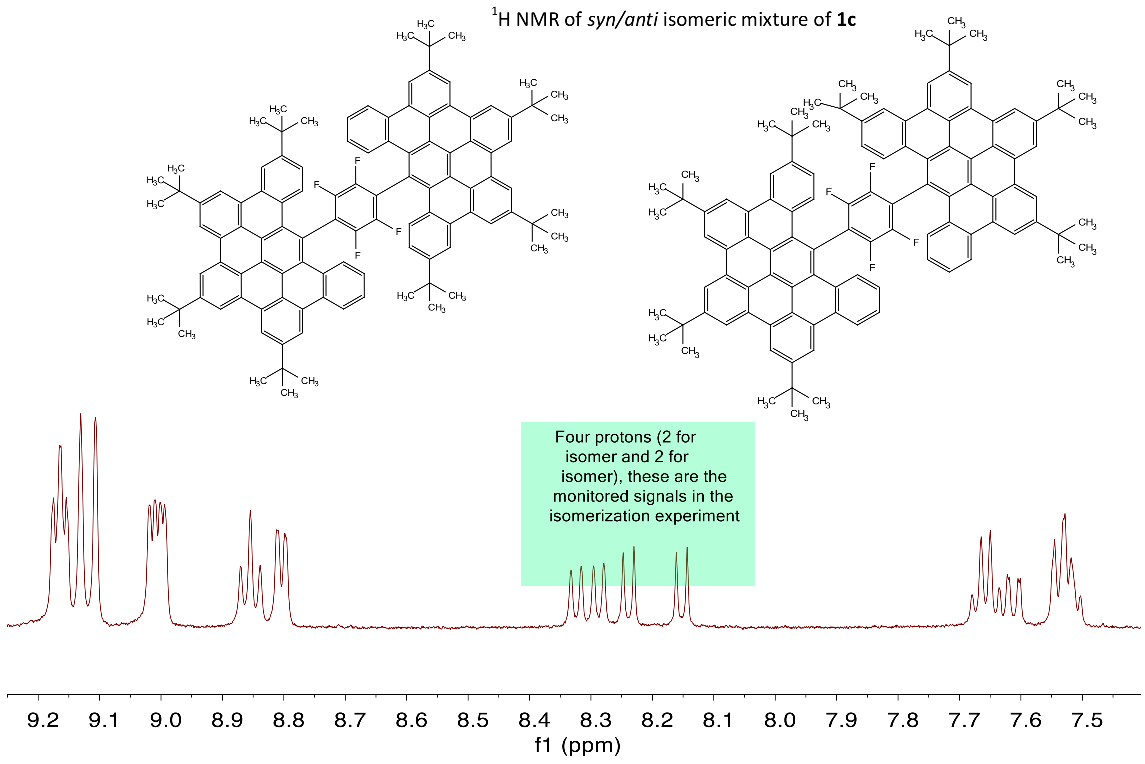

1H NMR of syn/anti isomeric mixture of 1c
Four protons (2 for isomer and 2 for isomer), these are the monitored signals in the isomerization experiment
f1 (ppm)

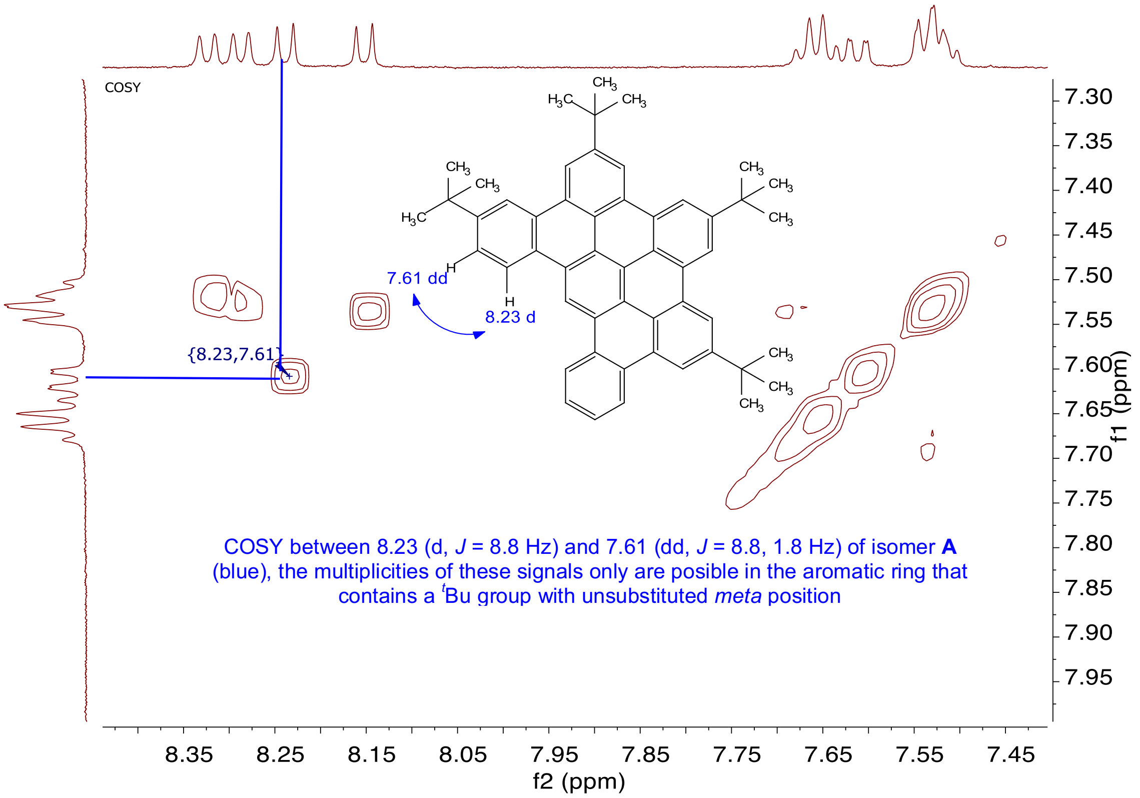

COSY
{8.23,7.61}
7.61 dd
8.23 d
COSY between 8.23 (d, J = 8.8 Hz) and 7.61 (dd, J = 8.8, 1.8 Hz) of isomer A (blue), the multiplicities of these signals only are posible in the aromatic ring that contains a tBu group with unsubstituted meta position
f2 (ppm)
f1 (ppm)

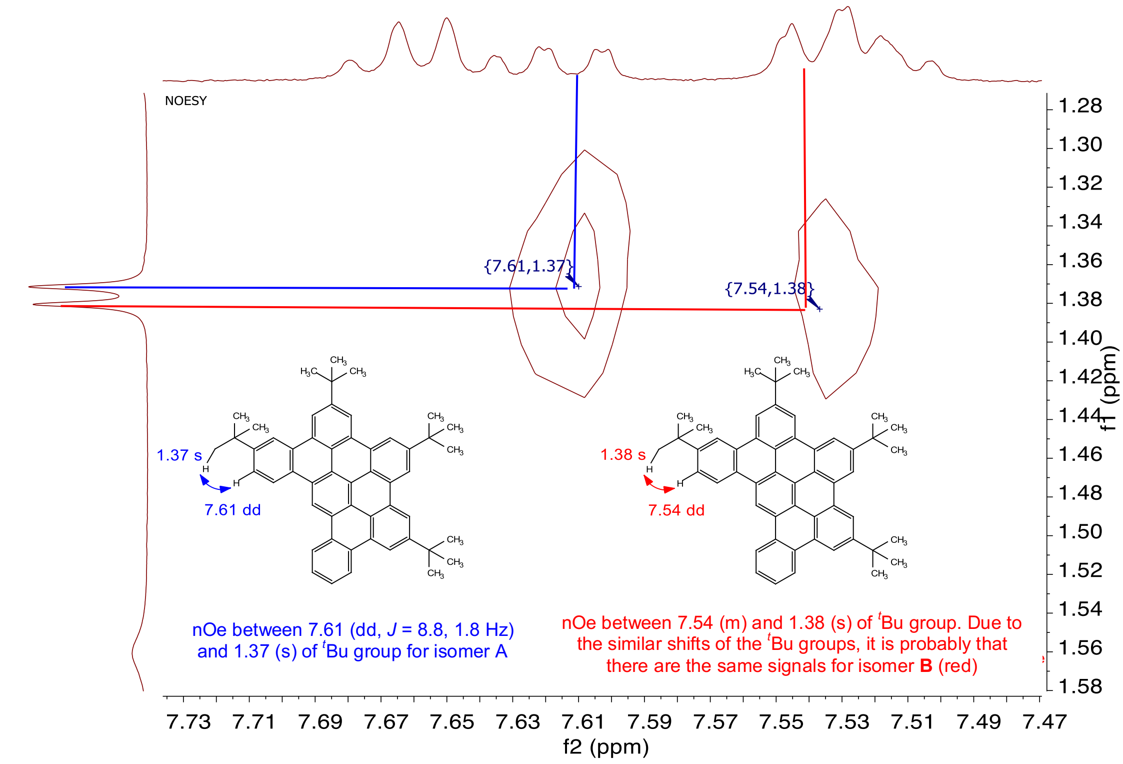
NOESY
{7.61,1.37}
{7.54,1.38}
1.37 s
7.61 dd
1.38 s
7.54 dd
nOe between 7.61 (dd, J = 8.8, 1.8 Hz)
and 1.37 (s) of tBu group for isomer A
nOe between 7.54 (m) and 1.38 (s) of tBu group. Due to
the similar shifts of the tBu groups, it is probably that
there are the same signals for isomer B (red)
f2 (ppm)
f1 (ppm)

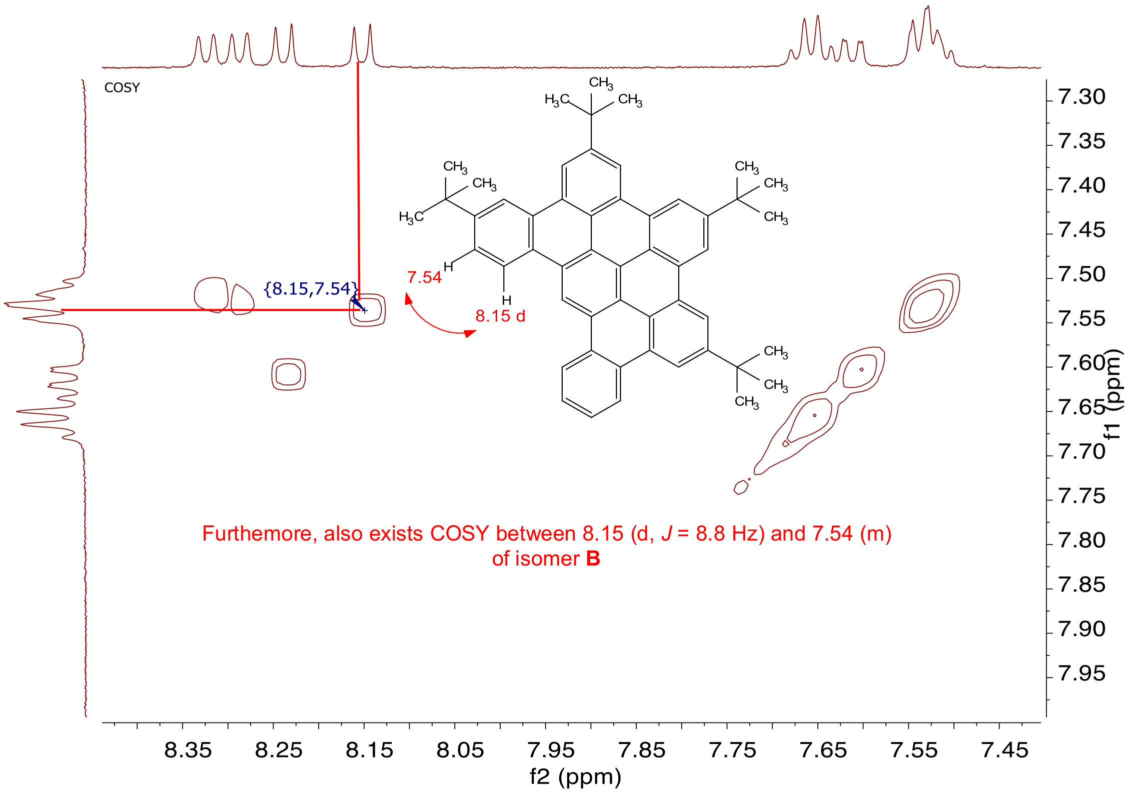
COSY
{8.15,7.54}
7.54
8.15 d
Furthemore, also exists COSY between 8.15 (d, J = 8.8 Hz) and 7.54 (m)
of isomer B
f2 (ppm)
f1 (ppm)

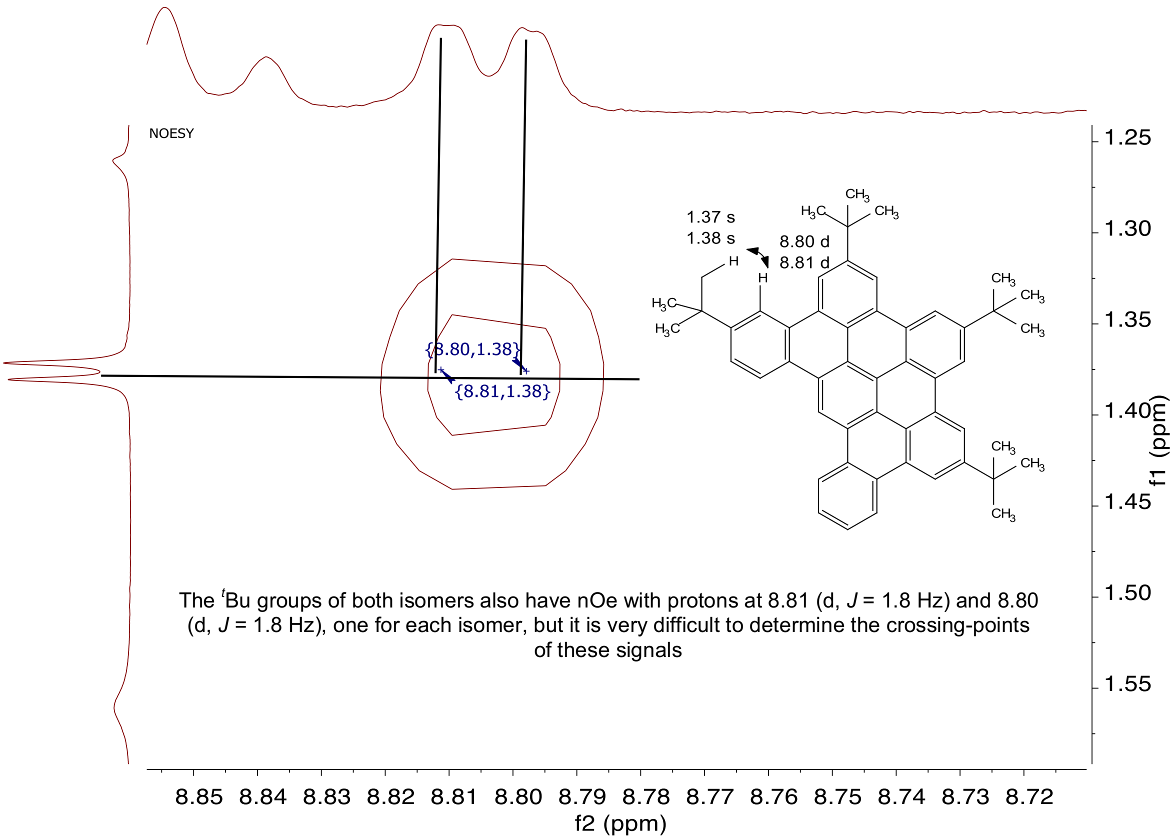
NOESY
1.37 s
1.38 s
8.80 d
8.81 d
{8.80,1.38}
{8.81,1.38}
The *t*Bu groups of both isomers also have nOe with protons at 8.81 (d, *J* = 1.8 Hz) and 8.80 (d, *J* = 1.8 Hz), one for each isomer, but it is very difficult to determine the crossing-points of these signals
f2 (ppm)
f1 (ppm)

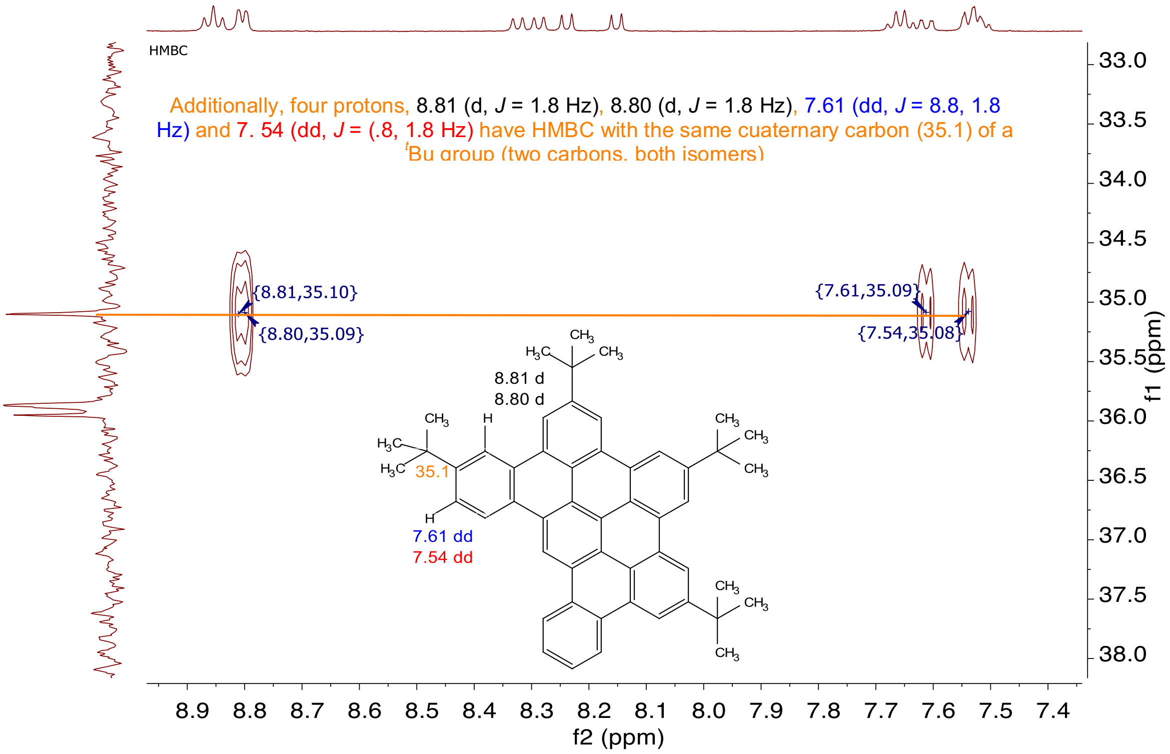
HMBC
Additionally, four protons, 8.81 (d, *J* = 1.8 Hz), 8.80 (d, *J* = 1.8 Hz), 7.61 (dd, *J* = 8.8, 1.8 Hz) and 7. 54 (dd, *J* = (.8, 1.8 Hz) have HMBC with the same cuaternary carbon (35.1) of a *t*Bu group (two carbons, both isomers)
{8.81,35.10}
{8.80,35.09}
{7.61,35.09}
{7.54,35.08}
8.81 d
8.80 d
35.1
7.61 dd
7.54 dd
f2 (ppm)
f1 (ppm)

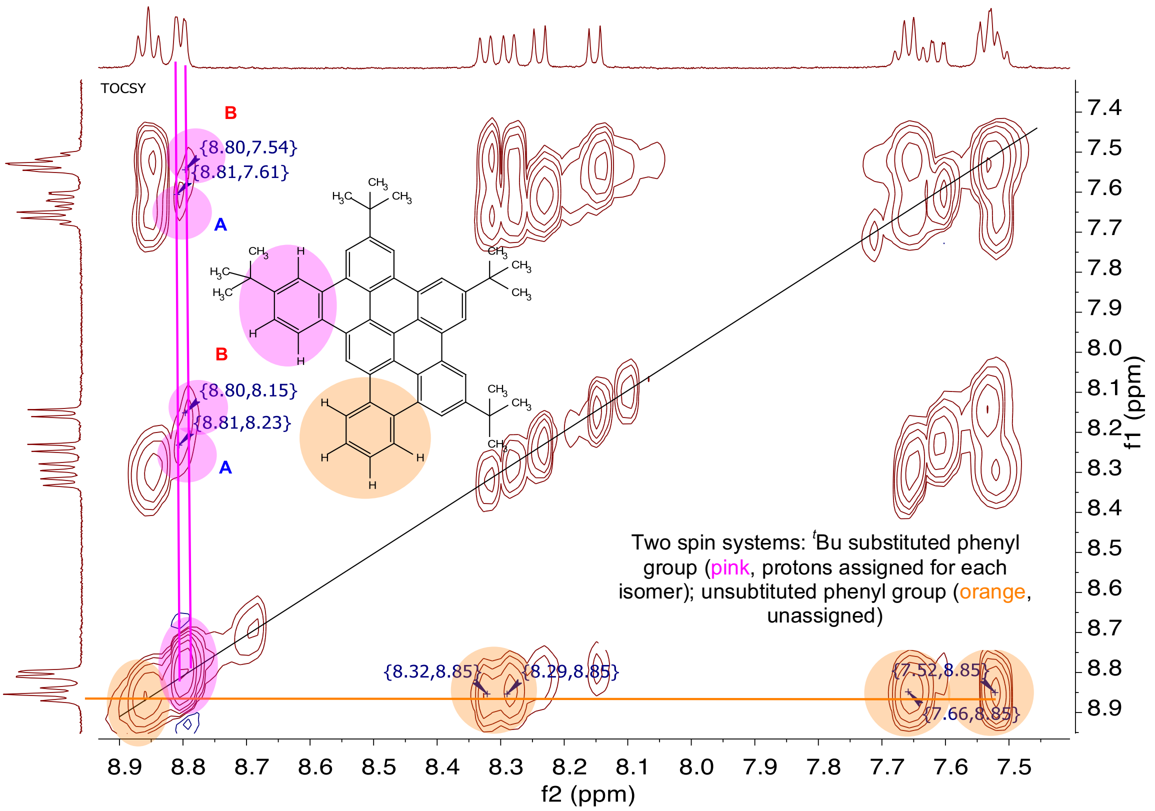
TOCSY
B
{8.80,7.54}
{8.81,7.61}
A
B
{8.80,8.15}
{8.81,8.23}
A
Two spin systems: ^{t}Bu substituted phenyl group (pink, protons assigned for each isomer); unsubtituted phenyl group (orange, unassigned)
{8.32,8.85}
{8.29,8.85}
{7.52,8.85}
{7.66,8.85}
f2 (ppm)
f1 (ppm)

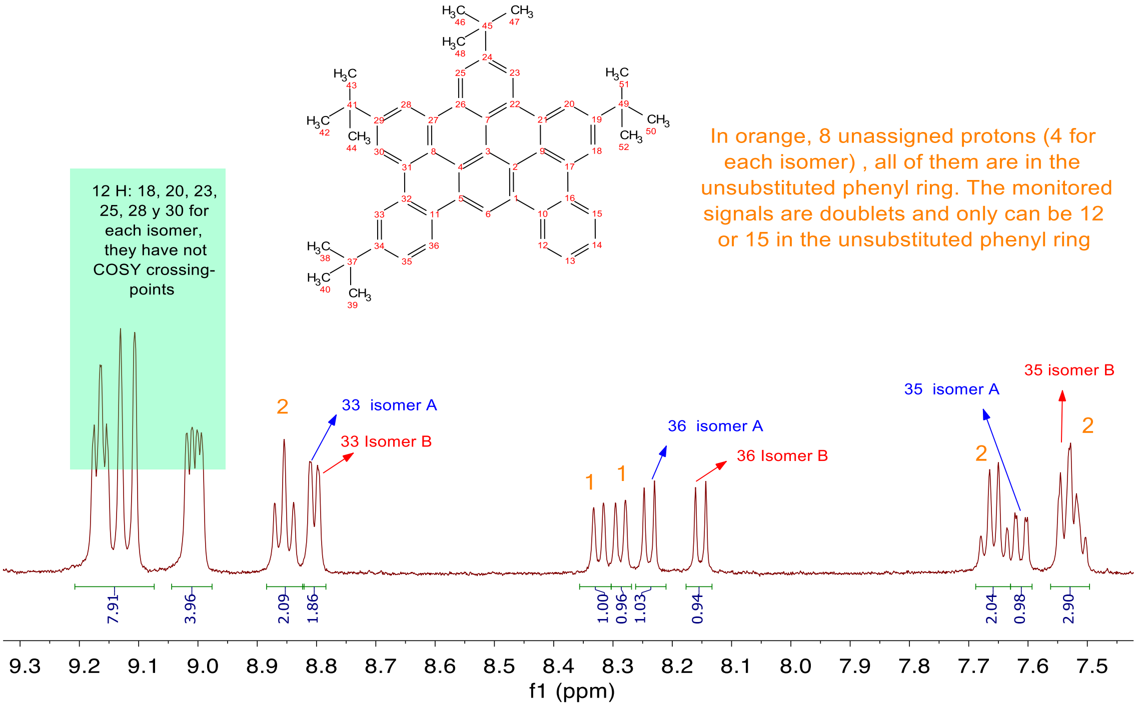
12 H: 18, 20, 23, 25, 28 y 30 for each isomer, they have not COSY crossing-points
In orange, 8 unassigned protons (4 for each isomer) , all of them are in the unsubstituted phenyl ring. The monitored signals are doublets and only can be 12 or 15 in the unsubstituted phenyl ring
33 isomer A
33 Isomer B
36 isomer A
36 Isomer B
35 isomer A
35 isomer B
7.91
3.96
2.09
1.86
1.00
0.96
1.03
0.94
2.04
0.98
2.90
f1 (ppm)

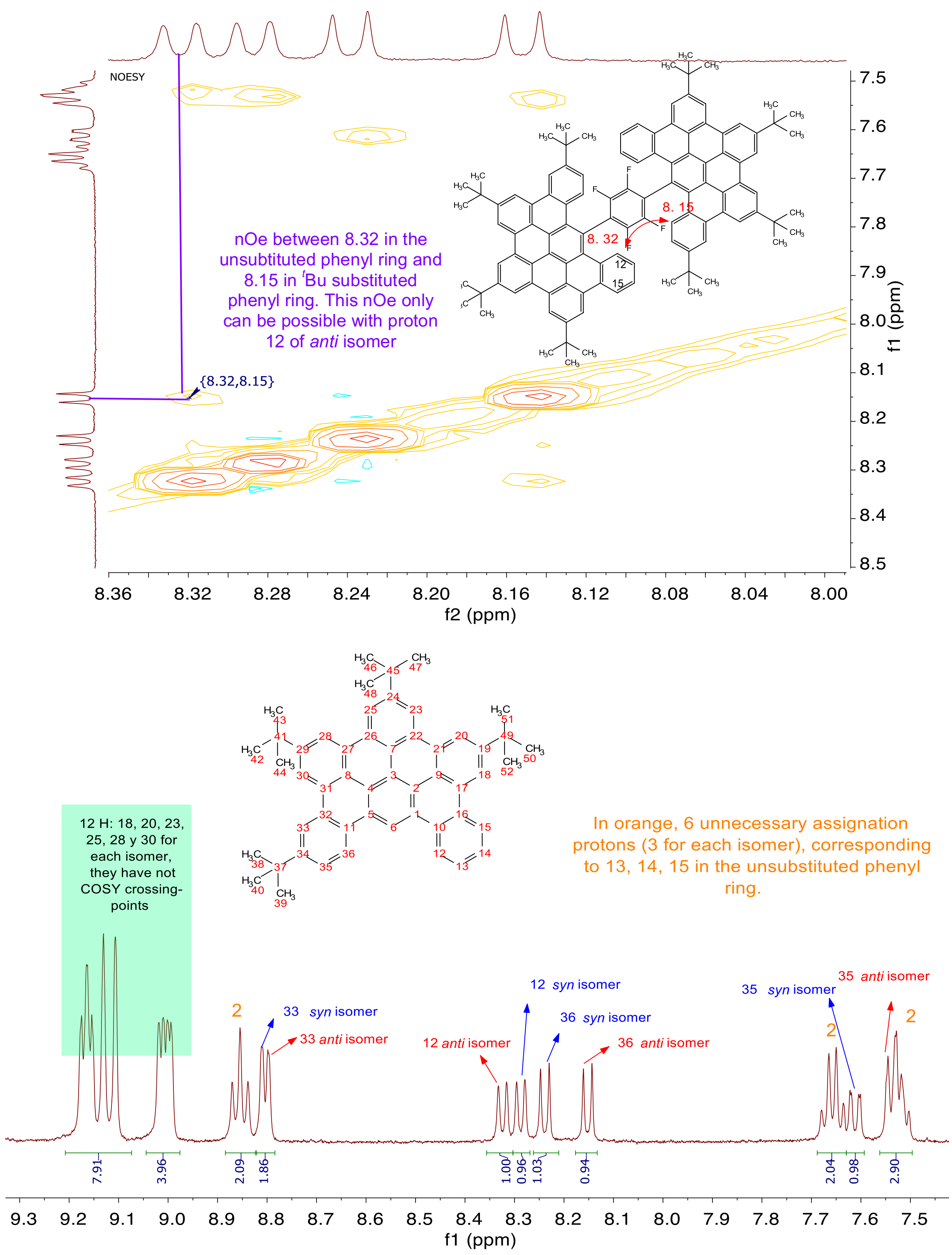

Figure S35. Assignation of *anti*/*syn* isomers **1c** by H,H-COSY, NOESY, HMBC and TOCSY.

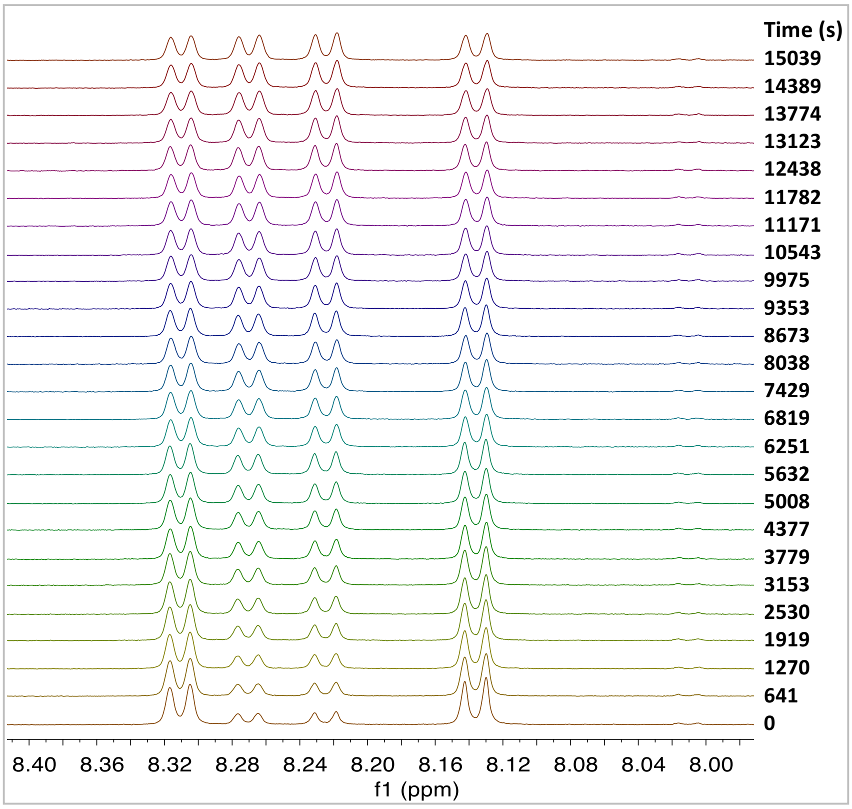

Figure S36. Isomerization at 320 K monitored with ^{1}H NMR.

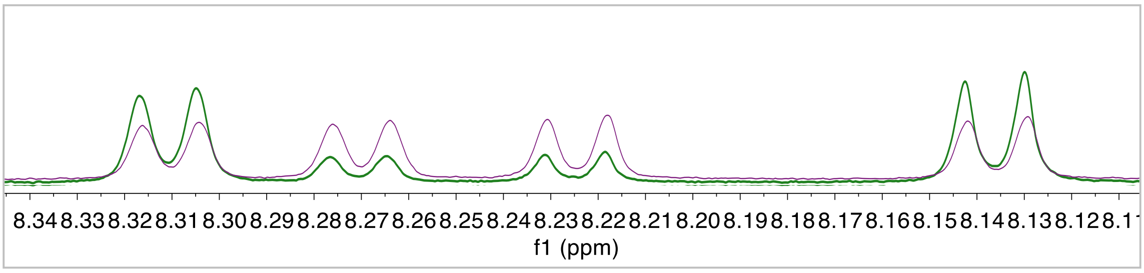

Figure S37. Initial (70:30 *anti*/*syn*, green) vs final (50:50 *anti*/*syn*, purple) situation.

Once the ^{1}H NMR data were acquired the isomerization constant at 320 K was determined.

$$Anti \underset{k_{-1}}{\overset{k_1}{\rightleftharpoons}} Syn \qquad \textbf{Equation 1}$$

$$-\frac{d[Anti]}{dt} = k_1[Anti] - k_{-1}[Syn] \qquad \textbf{Equation 2}$$

$$[Anti]_0 + [Syn]_0 = [Anti]_t + [Syn]_t = [Anti]_e + [Syn]_e \qquad \textbf{Equation 3}$$

$$\frac{[Syn]_e}{[Anti]_e} = k = \frac{k_1}{k_{-1}} = 1 \qquad \textbf{Equation 4}$$

$$-\frac{d[Anti]}{dt} = ([Anti]_t + [Anti]_e)(k_1 + k_{-1}) \qquad \textbf{Equation 5}$$

$$\frac{d[Anti]}{([Anti]_t + [Anti]_e)} = -(k_1 + k_{-1})dt \qquad \textbf{Equation 6}$$

$$\ln\frac{([Anti]_t - [Anti]_e)}{([Anti]_0 - [Anti]_e)} = -(k_1 + k_{-1})t \qquad \textbf{Equation 7}$$

Table S4. Data for isomerization constant determination.

Time (s)	$H_{8.31\ ppm}$ (%)	$X_{H,8.31\ ppm}$	$H_{8.27\ ppm}$ (%)	$X_{H,8.27\ ppm}$	$\ln((X_{H,8.31\ ppm}-X_e)/(X_0-X_e))$
0	66.80	0.6680	33.25	0.3325	0
641	64.52	0.6452	35.48	0.3548	-0.14717
1270	63.36	0.6336	36.64	0.3664	-0.23128
1919	61.68	0.6168	38.32	0.3832	-0.36719
2530	61.16	0.6116	38.84	0.3884	-0.41330
3153	59.60	0.5960	40.40	0.4040	-0.56594
3779	59.24	0.5924	40.76	0.4076	-0.60474
4377	57.79	0.5779	42.21	0.4221	-0.77830
5008	57.36	0.5736	42.64	0.4264	-0.83616
5632	56.09	0.5609	43.91	0.4391	-1.02962
6251	55.59	0.5559	44.41	0.4441	-1.11740
6819	54.92	0.5492	45.08	0.4508	-1.24857
7429	54.27	0.5427	45.73	0.4573	-1.39473
8038	53.88	0.5388	46.12	0.4612	-1.49393
8673	53.25	0.5325	46.75	0.4675	-1.67839
9353	52.89	0.5289	47.11	0.4711	-1.80141
9975	52.43	0.5243	47.57	0.4757	-1.98446
10543	52.07	0.5207	47.93	0.4793	-2.15549
11171	51.71	0.5171	48.29	0.4829	-2.36194
11782	51.41	0.5141	48.59	0.4859	-2.57399
12438	51.20	0.5120	48.80	0.4880	-2.75474
13123	50.88	0.5088	49.12	0.4912	-3.11412
13774	50.60	0.5060	49.40	0.4940	-3.58954
14389	50.24	0.5024	49.76	0.4976	-5.11560
15039	50.14	0.5014	49.86	0.4986	----

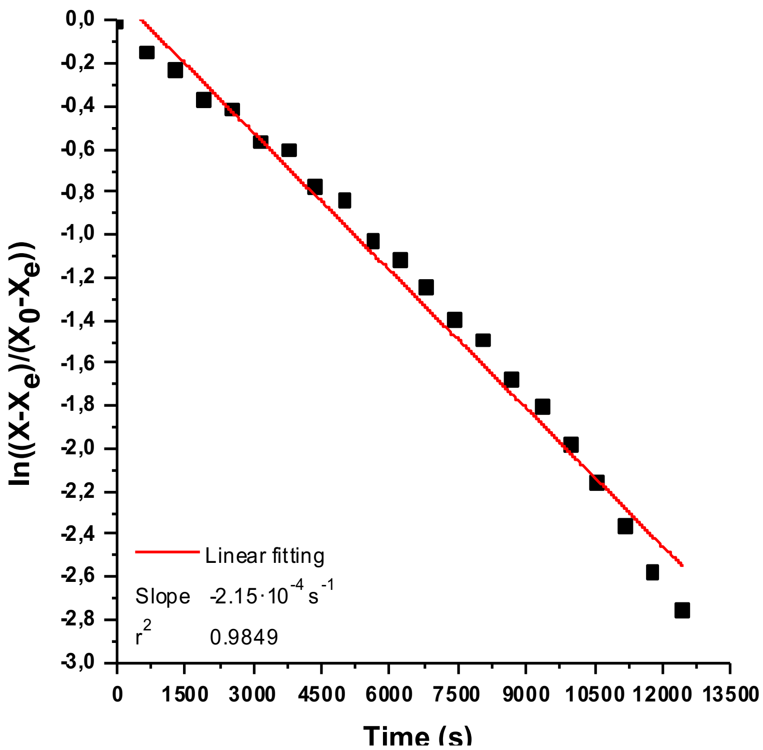

Figure S38. Representation of data from **Table S4**.

The data representation (**Figure S38**) gives the value of $-(k_1 + k_{-1})$ as the slope in units s^{-1}, **Equation 7.**

$$\ln\frac{([Anti]_t - [Anti]_e)}{([Anti]_0 - [Anti]_e)} = -(k_1 + k_{-1})t$$

Therefore $-(k_1 + k_{-1}) = -(2.15 \cdot 10^{-4} s^{-1})$, with $k_1 = k_{-1}$, if k_1 or k_{-1} is introduced in the Eyring's equation (**Equation 8**) $\Delta G^{\neq}$ and $t_{1/2}$ at 320 K can be determined as shown bellow.

$$k = \frac{\kappa k_B T}{h} e^{-\frac{\Delta G^{\neq}}{RT}}$$ **Equation 8**

$$k = 1.08 \cdot 10^{-4}\ (s^{-1})\ ;\ \kappa = 1\ (\text{for unimolecular reactions})$$
$$k_B = 1.38 \cdot 10^{-23}\ (J \cdot K^{-1})\ ;\ T = 320\ (K)$$
$$h = 6.62 \cdot 10^{-34}\ (J \cdot s)\ ;\ R = 8.31\ \ (J \cdot K^{-1} \cdot mol^{-1})$$

$$1.08 \cdot 10^{-4}(s^{-1}) = \frac{1.38 \cdot 10^{-23}(J \cdot K^{-1}) \cdot 320\ (K)}{6.62 \cdot 10^{-34}\ (J \cdot s)} \cdot e^{-\frac{\Delta G^{\neq}}{8.31\ (J \cdot K^{-1} \cdot mol^{-1}) \cdot 320\ (K)}}$$

$$\Delta \mathbf{G}^{\neq} = 100978.19(J \cdot mol^{-1}) = \mathbf{24.6(kcal \cdot mol^{-1})}\ \text{at 320 K}$$

$$\mathbf{t_{1/2}} = \frac{\ln 2}{k_1} = 6418.03\ s = \mathbf{106.97\ minutes}$$

6. HPLC separation

Analytical HPLC analysis was collected on an Agilent Technologies 1260 Infinity HPLC system using Regis Pirkle-type covalently modified silica column (4.6 × 250 mm) Whelk-O2 (R,R). Injection volume was 0.7 μL, flow rate was 1 mL/min and eluent was 98:1:1 hexane:isopropanol:tetrahydrofuran. Peaks were confirmed by comparison of their UV-Vis spectra to originate from the enantiomers.

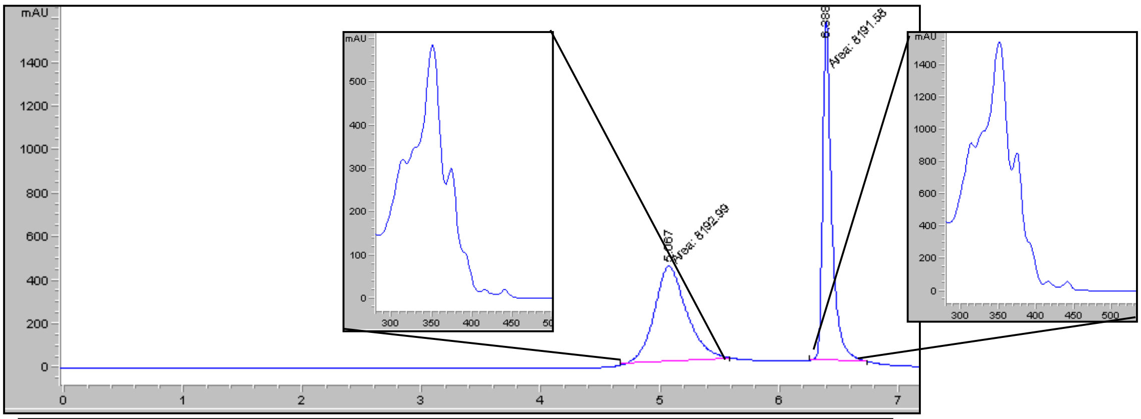

#	Time	Area	Height	Width	Area%	Symmetry
1	5.067	8193	435.2	0.3137	50.004	0.785
2	6.388	8191.6	1553.1	0.0879	49.996	0.601

Figure S39. HPLC chromatogram of racemic **1a** recorded at 360 nm.

7. Photophysical study

UV-Vis absorption spectra were recorded using a Cary 50 Conc spectrophotometer and quartz cells of 1.0 cm optical path length. Emission spectra were acquired in a Fluorolog TC-SPC HORIBA-JOVIN YVON spectrofluorometer in quartz cells of 1.0 cm optical path length. Samples were dissolved in chloroform (Carlo Erba, for analysis, ≥99%) and the solutions were in the 1–5 μM range.

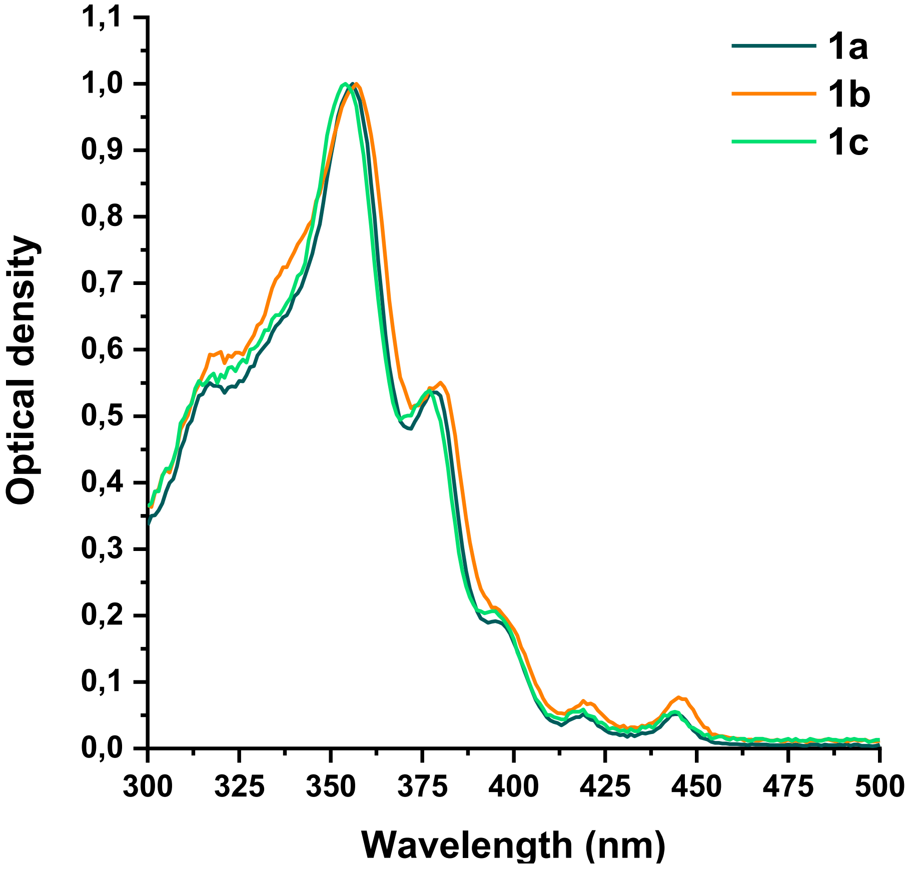

Figure S40. Normalized absorption spectra for compounds **1a**, **1b** and **1c**.

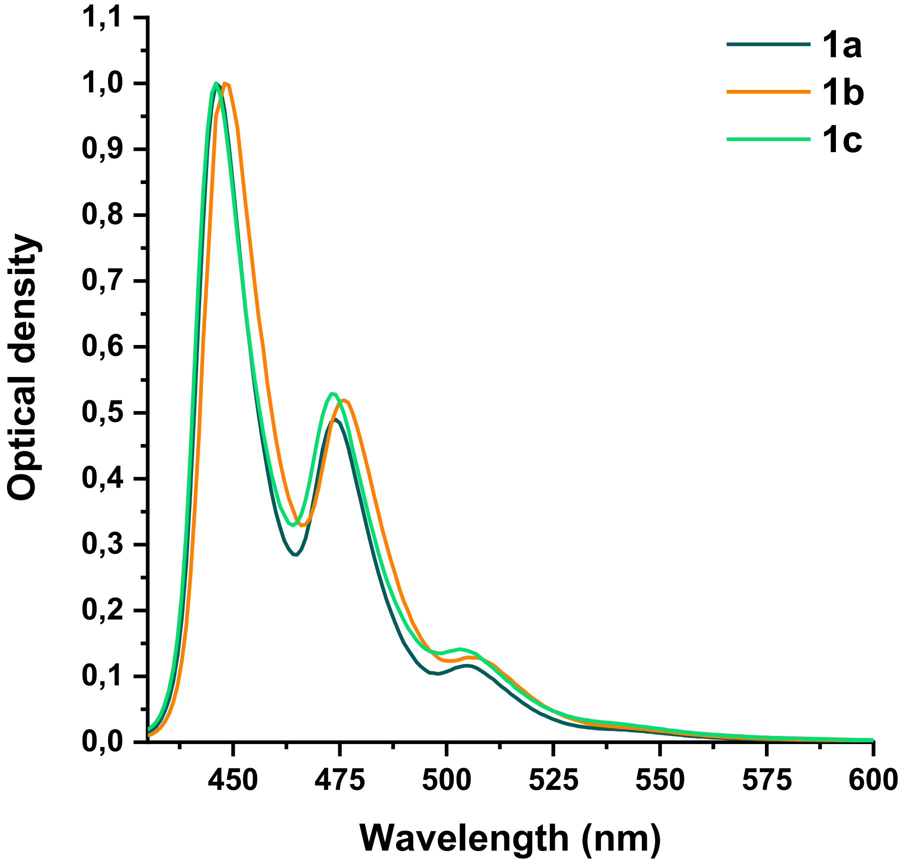

Figure S41. Normalized emission spectra for compounds **1a**, **1b** and **1c**.

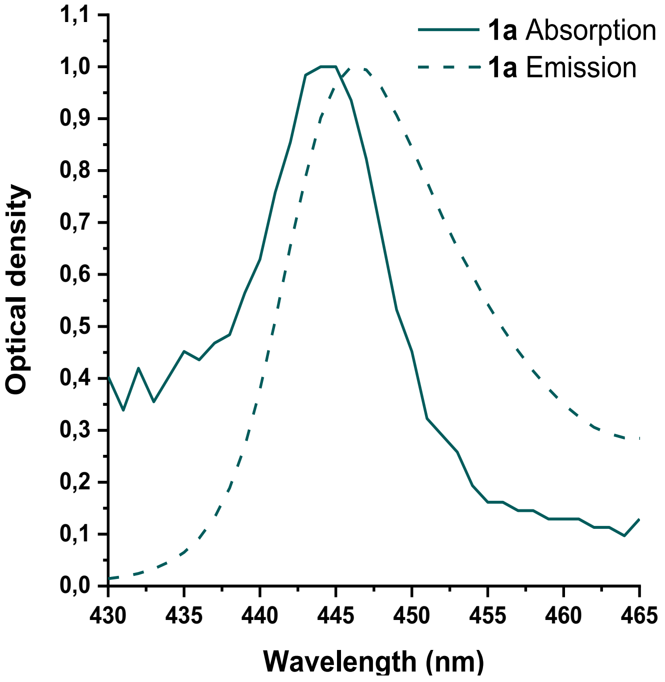

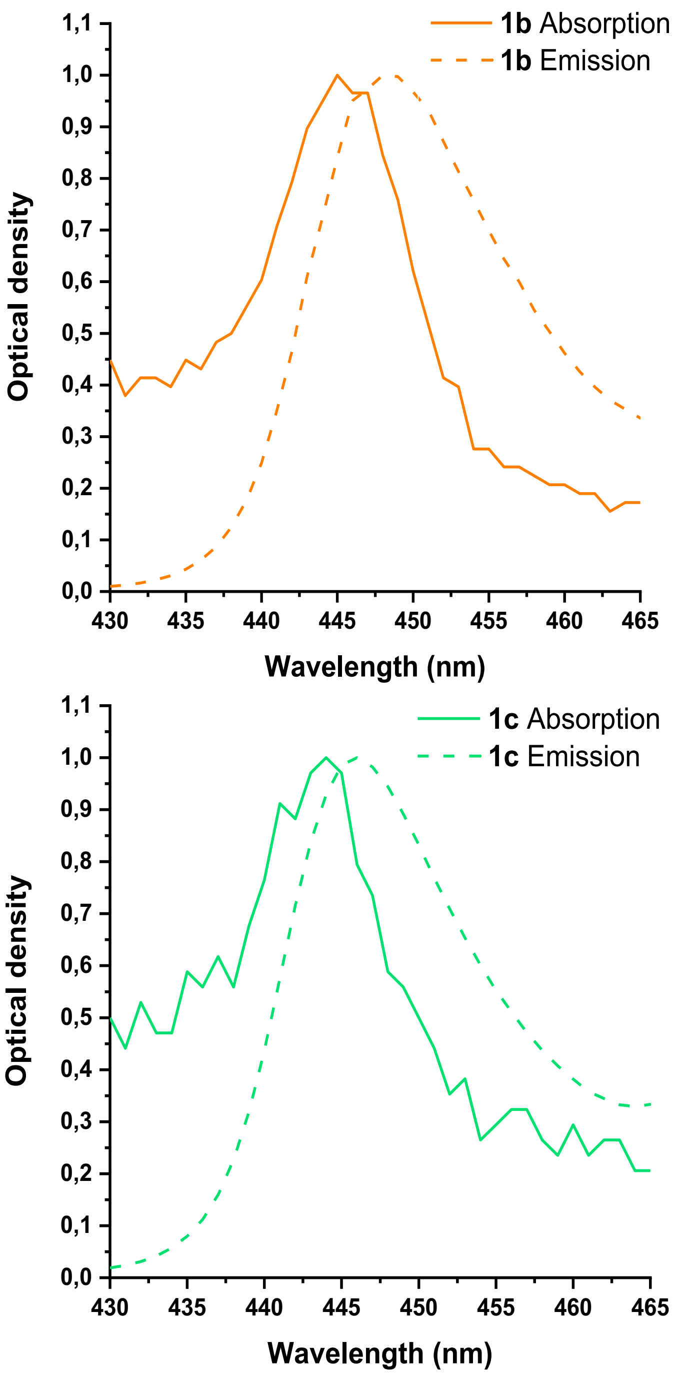

Figure S42. Normalized absorption and emission spectra for **1a**, **1b** and **1c**.

The energy of the 0-0 transition (E_{0-0}) of each compound was determined by converting to wavenumbers the wavelengths of the absorption and emission maxima, taking half the difference between the peaks, and adding these values to the wavenumber of the emission peak, which was then converted to eV units (Option a, **Table S5**). The calculated values agree well, within experimental error, with the wavelength of the intersection between the normalized absorption and emission spectra of each compound (Option b, **Table S6**).

Table S5. Option a. E_{0-0} determination, A is absorption maximum and E is emission maximum.

	A (nm)	A (cm^{-1})	E (nm)	E (cm^{-1})	(A-E)/2 (cm^{-1})	E+[(A-E)/2] (cm^{-1})	E+[(A-E)/2] **E_{0-0} (eV)**
**1a**	444	22522.52	446	22421.52	50.50	22472.02	**2.79**
**1b**	446	22421.52	448	22321.43	50.05	22371.48	**2.77**
**1c**	444	22522.52	446	22421.52	50.50	22472.02	**2.79**

Table S6. Option b. E_{0-0} determination considering the intersection between absorption and emmision bands.

	Intersection (nm)	**E_{0-0} (eV)**
**1a**	445	**2.79**
**1b**	446	**2.78**
**1c**	445	**2.79**

8. Electrochemical study

Electrochemical measurements were performed using a standard one-compartment, three-electrode electrochemical cell connected to an electrochemical analyzer (Metrohm Autolab). The working electrode was a glassy carbon electrode (3 mm diameter) that was freshly polished with a suspension of Al_2O_3 in distilled water and sonically rinsed with acetone before each measurement. Silver (Ag/0.1 M $AgNO_3$ in CH_3CN) and platinum wires were used as reference and counter electrodes, respectively. Electrochemical grade (Aldrich) tetrabutylammonium hexaflurophosphate 0.1 M in toluene:acetonitrile (4:1) was used as supporting electrolyte. All measurements were conducted under dry argon. Solutions were saturated with argon for deoxygenation and to maintain an argon blanket for at least 10 minutes prior to each measurement.

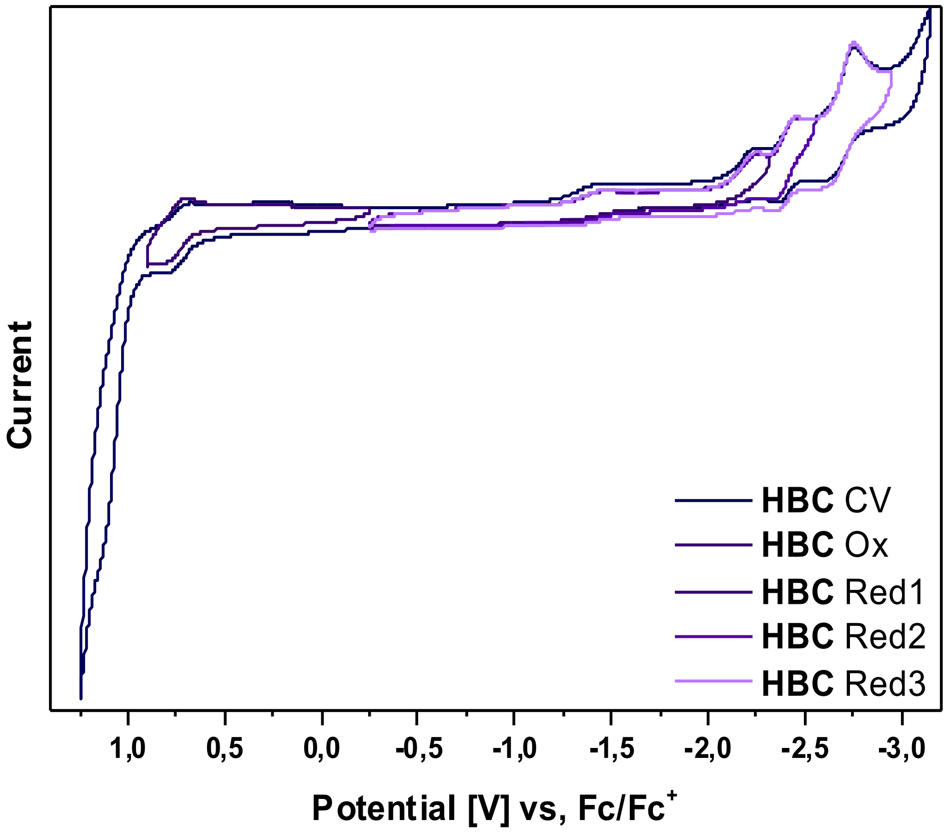

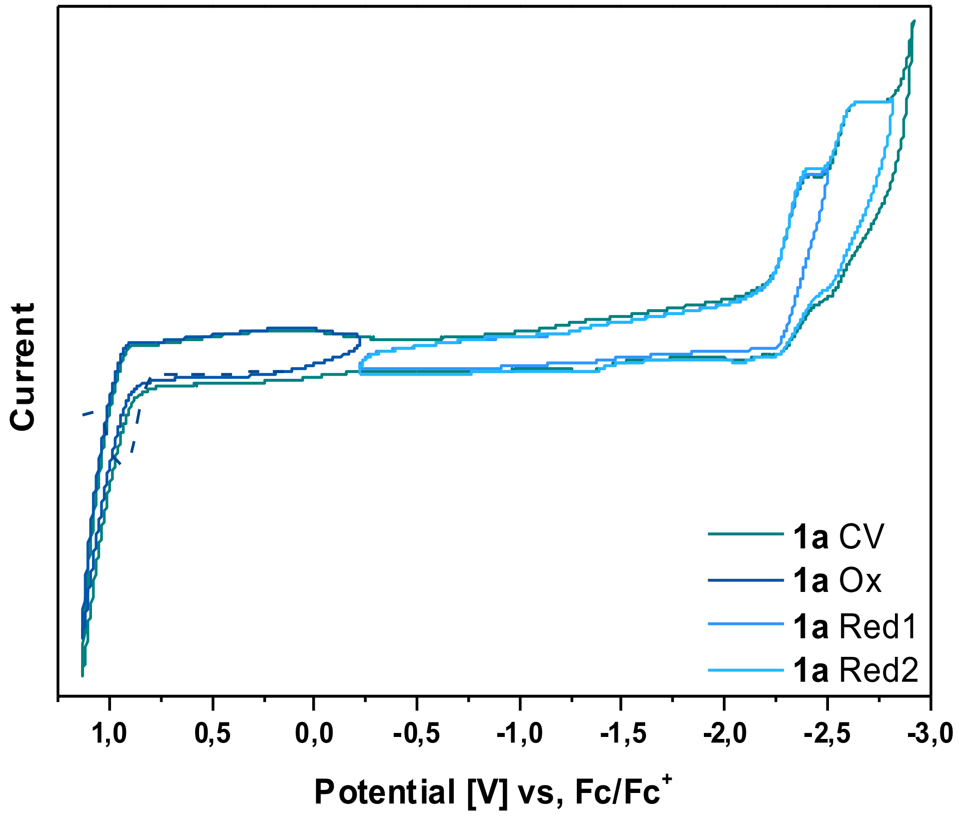
Current
1a CV
1a Ox
1a Red1
1a Red2
1,0
0,5
0,0
-0,5
-1,0
-1,5
-2,0
-2,5
-3,0
Potential [V] vs, Fc/Fc+

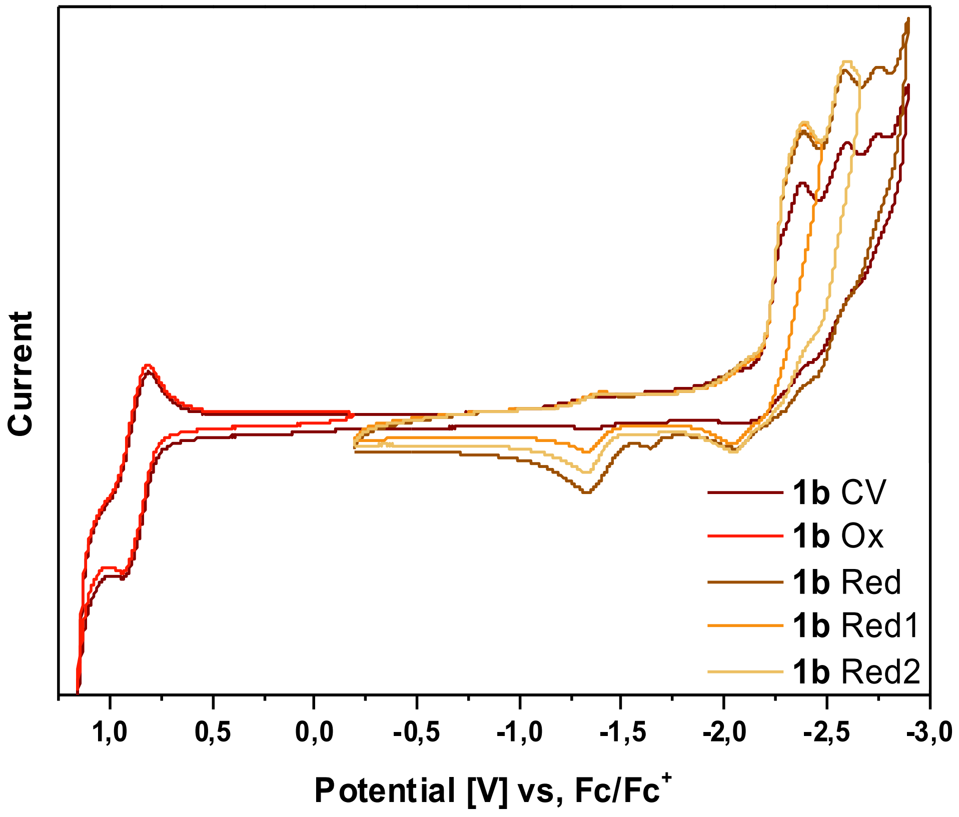
Current
1b CV
1b Ox
1b Red
1b Red1
1b Red2
1,0
0,5
0,0
-0,5
-1,0
-1,5
-2,0
-2,5
-3,0
Potential [V] vs, Fc/Fc+

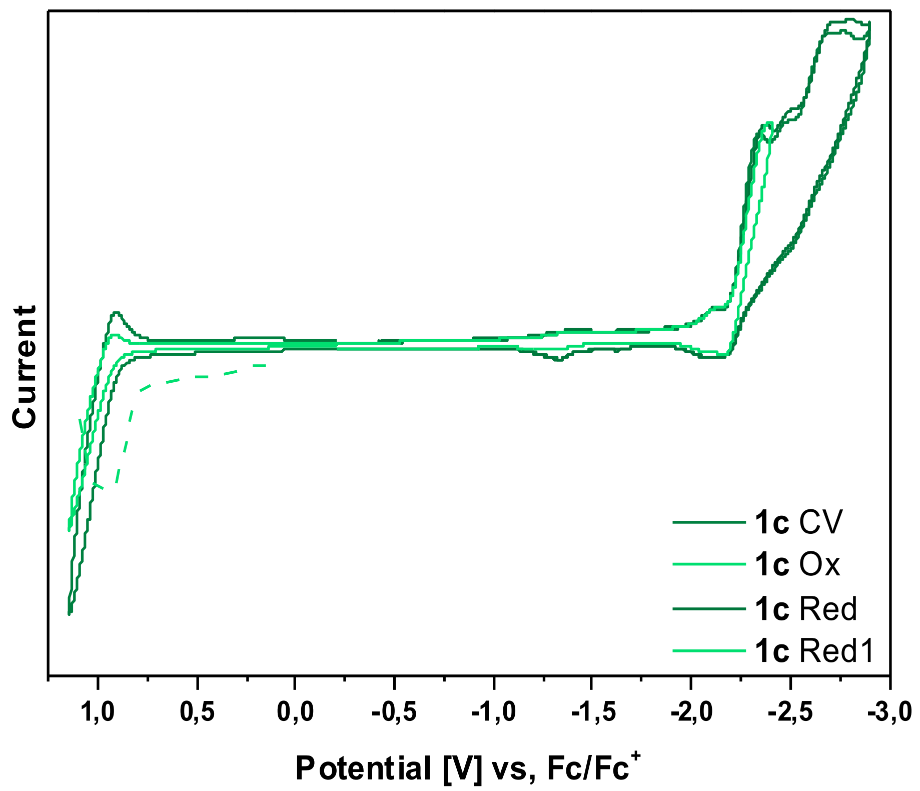

Figure S43. Ciclic voltamograms of **HCB**, **1a**, **1b** and **1c**. Square wave voltametry is represented like a dash line to show the oxidation of **1a** and **1c**.

Approximate energies of the HOMO and LUMO levels have been determined from the first onset oxidation and the first onset reduction potentials, respectively, using Ag/AgNO3 electrode as internal reference. $E_{HOMO/LUMO}$ has been calculated according the formula $E_{HOMO/LUMO}$ (eV) = – ($E_{ox/red}^{onset}$ + 4.14). The value of 4.14 represents the difference between the vacuum level potential of the normal hydrogen electrode NHE 4.44 eV and the potential of the $Ag/AgNO_3$ electrode.[3]

[3] Kucur, E.; Riegler, J.; Urban, G. A.; Nann, T. *The Journal of Chemical Physics* **2003**, 119 (4), 2333

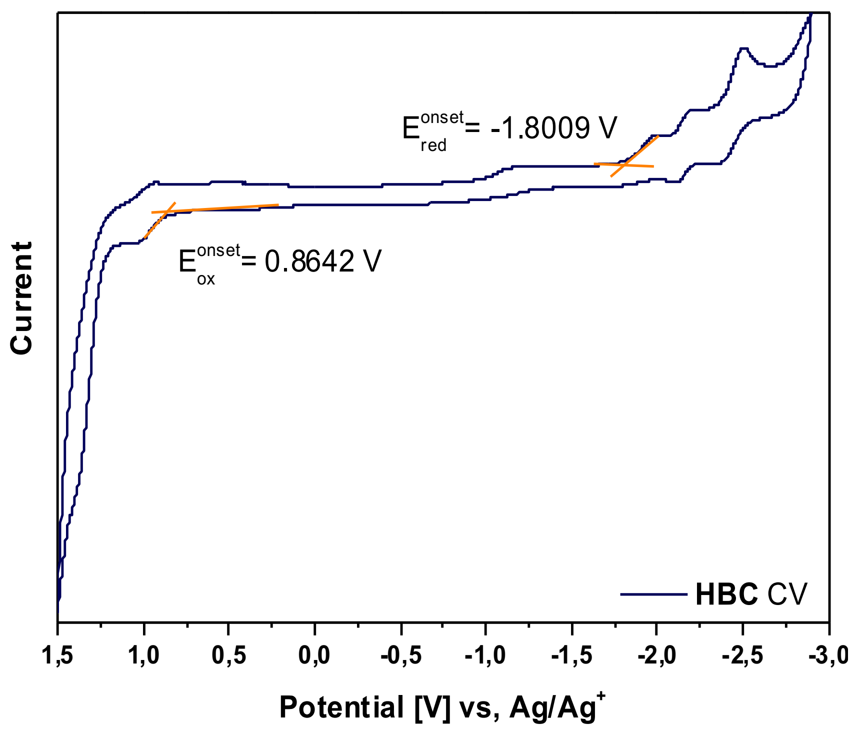

$$E_{HOMO} = -(E_{ox}^{onset} + 4.14) = -(0.8642 + 4.14) = -5.0042 \text{ eV}$$

$$E_{LUMO} = -(E_{red}^{onset} + 4.14) = -(-1.8009 + 4.14) = -2.3391 \text{ eV}$$

$$|\Delta \mathbf{E_{HOMO-LUMO}}| = |(-5.0042) - (-2.3391)| = \mathbf{2.6651\ eV}$$

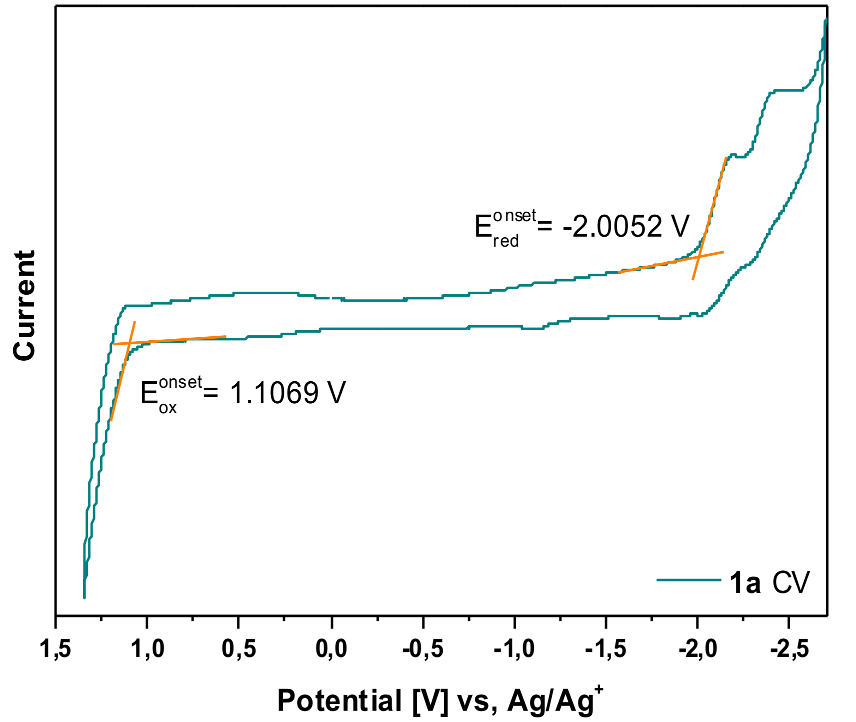

$$E_{HOMO} = -(E_{ox}^{onset} + 4.14) = -(1.1069 + 4.14) = -5.2469 \text{ eV}$$

$$E_{LUMO} = -(E_{red}^{onset} + 4.14) = -(-2.0052 + 4.14) = -2.1348 \text{ eV}$$

$$|\Delta \mathbf{E_{HOMO-LUMO}}| = |(-5.2469) - (-2.1348)| = \mathbf{3.1121\ eV}$$

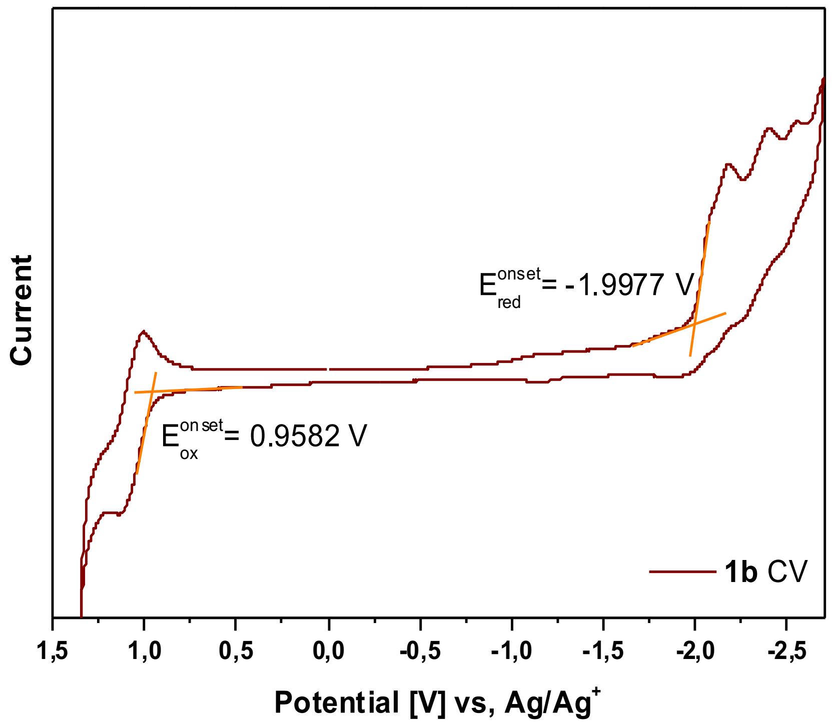

$$E_{HOMO} = -(E_{ox}^{onset} + 4.14) = -(0.9582 + 4.14) = -5.0982\text{ eV}$$

$$E_{LUMO} = -(E_{red}^{onset} + 4.14) = -(-1.9977 + 4.14) = -2.1423\text{ eV}$$

$$|\Delta \mathbf{E_{HOMO-LUMO}}| = |(-5.0982) - (-2.1423)| = \mathbf{2.9559\ eV}$$

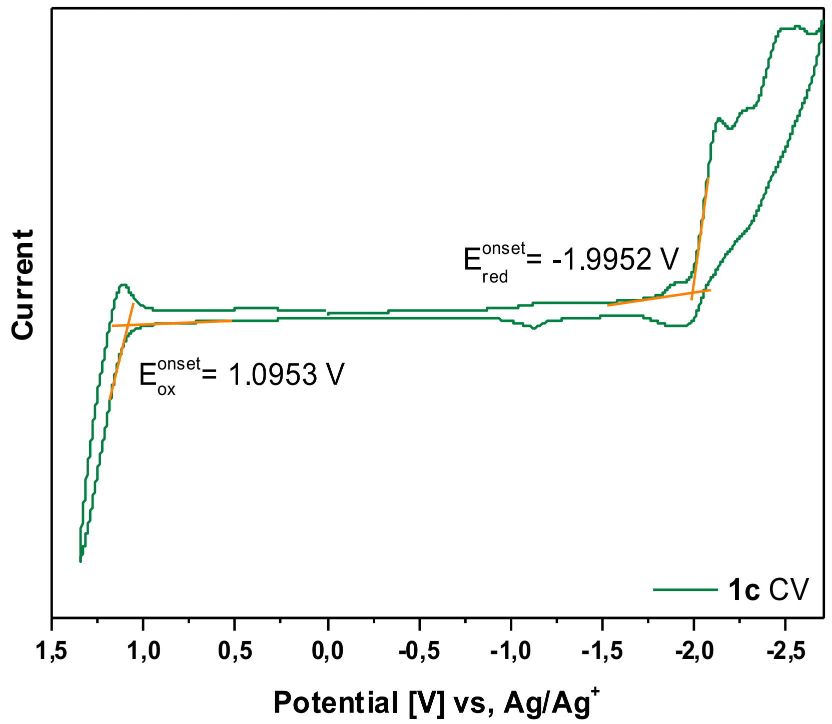

$$E_{HOMO} = -(E_{ox}^{onset} + 4.14) = -(1.0953 + 4.14) = -5.2353\text{ eV}$$

$$E_{LUMO} = -(E_{red}^{onset} + 4.14) = -(-1.9952 + 4.14) = -2.1448\text{ eV}$$

$$|\Delta \mathbf{E_{HOMO-LUMO}}| = |(-5.2353) - (-2.1448)| = \mathbf{3.0905\ eV}$$

As it is observed (**Figure S44**), the variation of the E_{0-0} (calculated from absorption-emission spectra) and the variation of the $E_{HOMO-LUMO}$ (calculated from CV voltamograms) for **1a**, **1b** and **1c** have the same tendency. Therefore, the obtained results are consistent.

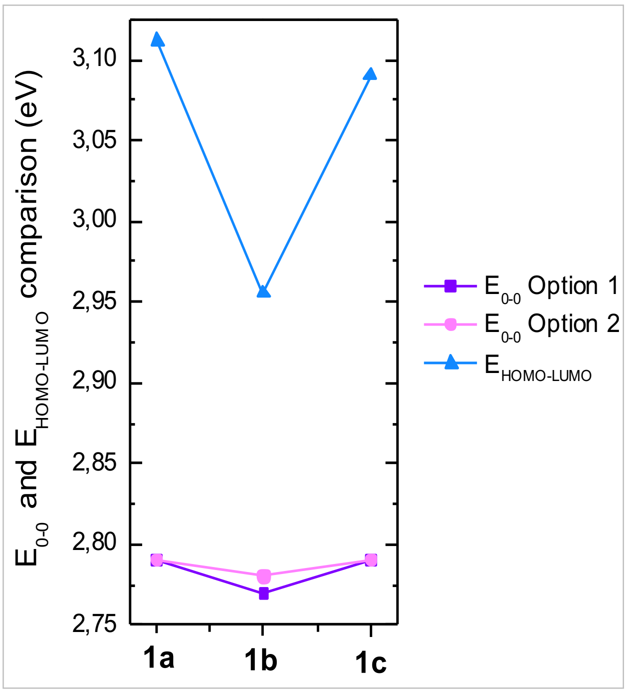

Figure S44. Variation of E_{0-0} and $E_{HOMO-LUMO}$ of compounds **1a**, **1b** and **1c**.

9. Computational Details

All the calculations reported in this paper were obtained with the GAUSSIAN 09 suite of programs.[4] Electron correlation was partially taken into account using the B3LYP[5] functional in conjuction with the D3 dispersion correction suggested by Grimme et al.[6] and the double-ζ quality plus polarization functions def2-SVP[7] basis set for all atoms. All species were characterized by frequency calculations,[8] and have positive definite Hessian matrices. This level is denoted B3LYP-D3/def2-SVP.

Calculations of the absorption spectrum were accomplished using time-dependent density functional theory (TD-DFT)[9] at the B3LYP-D3/def2-SVP level using the optimized geometries. The assignment of the excitation energies to the experimental bands was performed on the basis of the energy values and oscillator strengths. The B3LYP Hamiltonian was chosen because it was proven to provide reasonable UV-vis spectra for a variety of chromophores.[10]

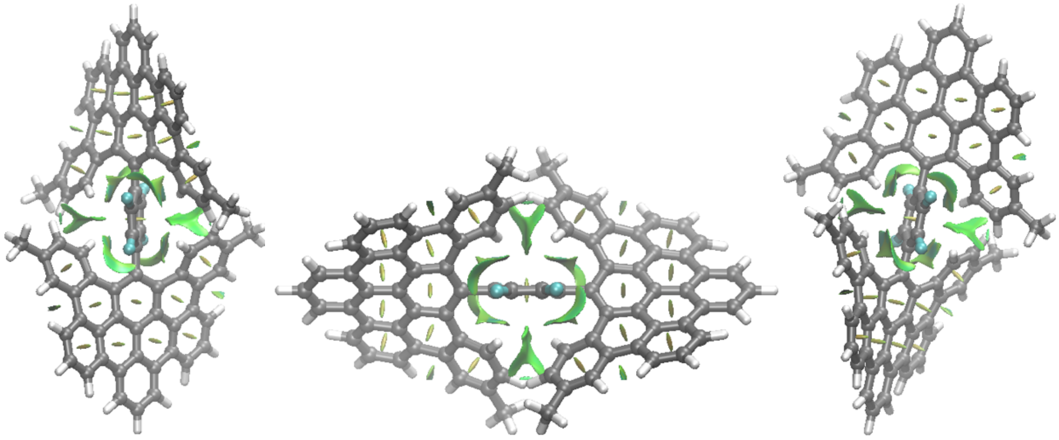

Figure S45. Different orientations of contour plots of the reduced density gradient isosurfaces (density cutoff of 0.04 a.u.) for compound **1a'**. The greenish surfaces indicate attractive non-covalent interactions.

---

[4] Gaussian 09, Revision D.01, M. J. Frisch, G. W. Trucks, H. B. Schlegel, G. E. Scuseria, M. A. Robb, J. R. Cheeseman, G. Scalmani, V. Barone, G. A. Petersson, H. Nakatsuji, X. Li, M. Caricato, A. Marenich, J. Bloino, B. G. Janesko, R. Gomperts, B. Mennucci, H. P. Hratchian, J. V. Ortiz, A. F. Izmaylov, J. L. Sonnenberg, D. Williams-Young, F. Ding, F. Lipparini, F. Egidi, J. Goings, B. Peng, A. Petrone, T. Henderson, D. Ranasinghe, V. G. Zakrzewski, J. Gao, N. Rega, G. Zheng, W. Liang, M. Hada, M. Ehara, K. Toyota, R. Fukuda, J. Hasegawa, M. Ishida, T. Nakajima, Y. Honda, O. Kitao, H. Nakai, T. Vreven, K. Throssell, J. A. Montgomery, Jr., J. E. Peralta, F. Ogliaro, M. Bearpark, J. J. Heyd, E. Brothers, K. N. Kudin, V. N. Staroverov, T. Keith, R. Kobayashi, J. Normand, K. Raghavachari, A. Rendell, J. C. Burant, S. S. Iyengar, J. Tomasi, M. Cossi, J. M. Millam, M. Klene, C. Adamo, R. Cammi, J. W. Ochterski, R. L. Martin, K. Morokuma, O. Farkas, J. B. Foresman, and D. J. Fox, Gaussian, Inc., Wallingford CT, 2016.

[5] a) A. D. Becke, *J. Chem. Phys*. **1993**, *98*, 5648; b) C. Lee, W. Yang, R. G. Parr, *Phys. Rev. B* **1998**, *37*, 785; c) S. H. Vosko, L. Wilk, M. Nusair, *Can. J. Phys*. **1980**, *58*, 1200.

[6] S. Grimme, J. Antony, S. Ehrlich, H. Krieg, H. *J. Chem. Phys*. **2010**, *132*, 154104.

[7] F. Weigend, R. Alhrichs, *Phys. Chem. Chem. Phys*. **2005**, *7*, 3297.

[8] J. W. McIver, A. K. Komornicki, *J. Am. Chem. Soc*. **1972**, *94*, 2625.

[9] a) M. E. Casida, *Recent Developments and Applications of Modern Density Functional Theory*; Elsevier: Amsterdam, 1996; Vol. 4; b) M. E. Casida, D. P. Chong, *Recent Advances in Density Functional Methods*; World Scientific: Singapore, 1995; Vol. 1, p 155.

[10] For a review, see: A. Dreuw, M. Head-Gordon, M. *Chem. Rev*. **2005**, *105*, 4009.

Cartesian coordinates (in Å) and total energies (in a.u.) of all the stationary points discussed in the text. All calculations have been performed at the B3LYP-D3/def2-SVP level.

1a': E= -3546.272109

C	0.695847000	-0.018276000	-1.192668000
C	-0.695774000	0.018459000	-1.192709000
C	-1.430536000	0.000052000	-0.000070000
C	-0.695843000	-0.018325000	1.192615000
C	0.695775000	0.018393000	1.192656000
C	1.430538000	0.000036000	0.000014000
F	1.334070000	0.046923000	2.364369000
F	-1.334210000	-0.046868000	2.364287000
F	-1.334061000	0.047062000	-2.364424000
F	1.334209000	-0.046770000	-2.364345000
C	5.036741000	-0.915169000	0.818456000
C	3.624467000	-1.032508000	0.678347000
C	2.920282000	0.000016000	0.000051000
C	3.624522000	1.032503000	-0.678237000
C	5.036797000	0.915110000	-0.818311000
C	5.747447000	-0.000041000	0.000083000
C	2.984883000	-2.211366000	1.287106000
C	5.744209000	-1.689443000	1.812507000
C	7.191041000	-0.000070000	0.000102000
C	5.744319000	1.689354000	-1.812346000
C	2.984997000	2.211379000	-1.287022000
C	3.631796000	-2.879040000	2.362354000
C	2.957489000	-3.932247000	3.021053000
C	1.708053000	-4.391218000	2.621431000
C	1.139110000	-3.809977000	1.471246000
C	1.766922000	-2.761549000	0.819486000
C	7.152111000	-1.530922000	1.970439000
C	7.779564000	-2.128345000	3.077477000
C	7.051385000	-2.885628000	3.990172000
C	5.690176000	-3.107190000	3.792023000
C	5.020369000	-2.545407000	2.693902000
C	7.901909000	0.756400000	-0.977577000
C	9.306519000	0.743377000	-0.952001000
C	9.998529000	-0.000129000	0.000134000
C	9.306464000	-0.743606000	0.952254000
C	7.901856000	-0.756568000	0.977798000
C	5.020533000	2.545343000	-2.693765000
C	5.690394000	3.107109000	-3.791858000
C	7.051604000	2.885503000	-3.989965000
C	7.779729000	2.128194000	-3.077251000
C	7.152221000	1.530786000	-1.970234000
C	1.767050000	2.761620000	-0.819425000
C	1.139300000	3.810074000	-1.471195000
C	1.708291000	4.391286000	-2.621376000
C	2.957708000	3.932257000	-3.020974000
C	3.631962000	2.879023000	-2.362253000
C	-3.631906000	2.878916000	2.362392000
C	-2.957636000	3.932103000	3.021171000
C	-1.708223000	4.391149000	2.621581000
C	-1.139246000	3.809997000	1.471363000
C	-1.767011000	2.761588000	0.819537000
C	-7.152176000	1.530711000	1.970365000
C	-7.779659000	2.128055000	3.077429000
C	-7.051513000	2.885313000	3.990171000

C	-5.690309000	3.106927000	3.792049000
C	-5.020472000	2.545222000	2.693907000
C	-7.901875000	-0.756473000	-0.977784000
C	-9.306485000	-0.743514000	-0.952207000
C	-9.998527000	-0.000093000	-0.000030000
C	-9.306495000	0.743360000	0.952132000
C	-7.901888000	0.756384000	0.977677000
C	-5.020426000	-2.545233000	-2.694035000
C	-5.690256000	-3.106961000	-3.792169000
C	-7.051468000	-2.885385000	-3.990281000
C	-7.779628000	-2.128145000	-3.077536000
C	-7.152153000	-1.530777000	-1.970480000
C	-1.766945000	-2.761481000	-0.819685000
C	-1.139150000	-3.809876000	-1.471507000
C	-1.708122000	-4.391062000	-2.621710000
C	-2.957555000	-3.932066000	-3.021291000
C	-3.631851000	-2.878889000	-2.362522000
C	-2.984962000	2.211333000	1.287110000
C	-5.744278000	1.689288000	1.812453000
C	-7.191040000	-0.000029000	-0.000062000
C	-5.744248000	-1.689308000	-1.812586000
C	-2.984919000	-2.211274000	-1.287254000
C	-5.747446000	-0.000002000	-0.000074000
C	-5.036777000	0.915102000	0.818358000
C	-3.624505000	1.032501000	0.678262000
C	-2.920280000	0.000047000	-0.000100000
C	-3.624481000	-1.032439000	-0.678431000
C	-5.036760000	-0.915086000	-0.818510000
H	-3.442473000	-4.437407000	-3.857062000
H	-0.194026000	-4.199135000	-1.084844000
H	-1.315577000	-2.376802000	0.090973000
H	-5.149098000	-3.725779000	-4.507513000
H	-7.552144000	-3.318897000	-4.859383000
H	-8.846654000	-1.984236000	-3.246569000
H	-9.873896000	-1.330125000	-1.674411000
H	-11.091247000	-0.000119000	-0.000013000
H	-9.873920000	1.329938000	1.674354000
H	-8.846680000	1.984116000	3.246469000
H	-7.552194000	3.318807000	4.859279000
H	-5.149163000	3.725755000	4.507393000
H	-3.442559000	4.437416000	3.856956000
H	-0.194141000	4.199295000	1.084690000
H	-1.315645000	2.376939000	-0.091134000
H	3.442653000	4.437625000	-3.856713000
H	1.315679000	2.376928000	0.091224000
H	5.149269000	3.725979000	-4.507181000
H	7.552302000	3.319049000	-4.859037000
H	8.846753000	1.984264000	-3.246276000
H	9.873958000	1.330000000	-1.674173000
H	11.091249000	-0.000155000	0.000148000
H	9.873862000	-1.330255000	1.674439000
H	8.846587000	-1.984446000	3.246537000
H	7.552043000	-3.319183000	4.859263000
H	5.149003000	-3.726038000	4.507329000
H	1.315579000	-2.376835000	-0.091168000
H	0.194000000	-4.199221000	1.084535000
H	3.442396000	-4.437646000	3.856795000
H	0.194201000	4.199367000	-1.084504000
C	-0.991053000	5.494343000	3.356641000
H	-0.655108000	6.283943000	2.664546000
H	-1.629341000	5.959398000	4.122570000

H	-0.089441000	5.106425000	3.861142000
C	0.991106000	5.494464000	-3.356445000
H	0.090112000	5.106296000	-3.861865000
H	0.654171000	6.283533000	-2.664231000
H	1.629741000	5.960238000	-4.121649000
C	-0.990906000	-5.494209000	-3.356797000
H	-0.654446000	-6.283550000	-2.664659000
H	-1.629346000	-5.959625000	-4.122380000
H	-0.089609000	-5.106128000	-3.861741000
C	0.990783000	-5.494309000	3.356548000
H	0.090317000	-5.105862000	3.862702000
H	0.652992000	-6.282950000	2.664269000
H	1.629670000	-5.960683000	4.121178000

1c′-syn: E= -3703.302470

C	-0.677530000	-0.632756000	-1.203223000
C	0.714173000	-0.666619000	-1.182043000
C	1.430315000	-0.649907000	0.022023000
C	0.677544000	-0.632768000	1.203225000
C	-0.714159000	-0.666633000	1.182044000
C	-1.430301000	-0.649910000	-0.022021000
F	-1.370673000	-0.689589000	2.343589000
F	1.298644000	-0.603727000	2.384085000
F	1.370686000	-0.689566000	-2.343588000
F	-1.298630000	-0.603709000	-2.384083000
C	-5.041108000	0.286574000	0.747780000
C	-3.627556000	0.396732000	0.618627000
C	-2.920392000	-0.644306000	-0.043697000
C	-3.622352000	-1.676379000	-0.724444000
C	-5.033181000	-1.554410000	-0.876763000
C	-5.747961000	-0.632932000	-0.068696000
C	-2.990514000	1.580422000	1.218318000
C	-5.753496000	1.075031000	1.727267000
C	-7.191478000	-0.629225000	-0.078670000
C	-5.736095000	-2.327972000	-1.874822000
C	-2.980993000	-2.860831000	-1.324581000
C	-3.642820000	2.269640000	2.274067000
C	-2.974374000	3.334787000	2.922537000
C	-1.718637000	3.789013000	2.535138000
C	-1.144942000	3.174909000	1.402330000
C	-1.765875000	2.119549000	0.758772000
C	-7.162660000	0.920225000	1.877753000
C	-7.796756000	1.533464000	2.972291000
C	-7.073791000	2.303098000	3.878765000
C	-5.711185000	2.521070000	3.686461000
C	-5.034274000	1.943113000	2.601051000
C	-7.897460000	-1.389991000	-1.056381000
C	-9.302105000	-1.377473000	-1.037986000
C	-9.998878000	-0.628907000	-0.093314000
C	-9.311591000	0.121445000	0.856868000
C	-7.907089000	0.135295000	0.889292000
C	-5.007977000	-3.184770000	-2.751121000
C	-5.670042000	-3.743913000	-3.855143000
C	-7.029383000	-3.519626000	-4.063147000
C	-7.762889000	-2.762278000	-3.154834000
C	-7.142393000	-2.166706000	-2.042858000
C	-1.770430000	-3.410988000	-0.838856000
C	-1.135731000	-4.464811000	-1.481195000
C	-1.710613000	-5.031339000	-2.627305000
C	-2.948872000	-4.582075000	-3.058346000

C	-3.623892000	-3.524440000	-2.407678000
C	3.623903000	-3.524437000	2.407671000
C	2.948876000	-4.582067000	3.058339000
C	1.710602000	-5.031306000	2.627313000
C	1.135708000	-4.464753000	1.481220000
C	1.770415000	-3.410937000	0.838881000
C	7.142411000	-2.166714000	2.042844000
C	7.762909000	-2.762295000	3.154814000
C	7.029405000	-3.519649000	4.063123000
C	5.670063000	-3.743932000	3.855122000
C	5.007995000	-3.184781000	2.751106000
C	7.907101000	0.135319000	-0.889283000
C	9.311603000	0.121470000	-0.856861000
C	9.998892000	-0.628892000	0.093311000
C	9.302120000	-1.377469000	1.037976000
C	7.897475000	-1.389988000	1.056373000
C	5.034281000	1.943144000	-2.601031000
C	5.711191000	2.521112000	-3.686435000
C	7.073800000	2.303149000	-3.878737000
C	7.796766000	1.533511000	-2.972267000
C	7.162671000	0.920259000	-1.877736000
C	1.765851000	2.119505000	-0.758791000
C	1.144906000	3.174857000	-1.402349000
C	1.718608000	3.788987000	-2.535140000
C	2.974362000	3.334791000	-2.922525000
C	3.642820000	2.269652000	-2.274055000
C	2.981001000	-2.860814000	1.324586000
C	5.736112000	-2.327977000	1.874811000
C	7.191491000	-0.629212000	0.078671000
C	5.753506000	1.075058000	-1.727252000
C	2.990514000	1.580417000	-1.218318000
C	5.747975000	-0.632922000	0.068697000
C	5.033196000	-1.554406000	0.876759000
C	3.622367000	-1.676369000	0.724447000
C	2.920406000	-0.644301000	0.043699000
C	3.627567000	0.396736000	-0.618626000
C	5.041120000	0.286589000	-0.747773000
H	3.484716000	3.839421000	-3.738302000
H	0.193008000	3.538360000	-1.015077000
H	1.301059000	1.719933000	0.138257000
H	5.174485000	3.149647000	-4.396760000
H	7.579764000	2.749383000	-4.738287000
H	8.865049000	1.393424000	-3.136281000
H	9.882874000	0.712795000	-1.572096000
H	11.091588000	-0.629697000	0.098077000
H	9.865631000	-1.968497000	1.759661000
H	8.828377000	-2.615980000	3.331495000
H	7.524316000	-3.951247000	4.936439000
H	5.124715000	-4.363236000	4.566930000
H	3.421958000	-5.089722000	3.898812000
H	0.195277000	-4.856852000	1.088766000
H	1.330127000	-3.021579000	-0.075066000
H	-3.421953000	-5.089716000	-3.898828000
H	-1.330154000	-3.021657000	0.075107000
H	-5.124693000	-4.363216000	-4.566952000
H	-7.524292000	-3.951218000	-4.936467000
H	-8.828356000	-2.615962000	-3.331517000
H	-9.865614000	-1.968494000	-1.759678000
H	-11.091574000	-0.629713000	-0.098081000
H	-9.882864000	0.712762000	1.572108000
H	-8.865038000	1.393372000	3.136305000

H	-7.579756000	2.749324000	4.738320000
H	-5.174481000	3.149606000	4.396787000
H	-1.301095000	1.720008000	-0.138294000
H	-0.193063000	3.538441000	1.015041000
H	-3.484729000	3.839400000	3.738324000
H	-0.195317000	-4.856934000	-1.088725000
H	1.211939000	-5.851049000	3.149934000
H	-1.211958000	-5.851087000	-3.149924000
C	0.971140000	4.930194000	-3.241589000
C	-0.971186000	4.930237000	3.241579000
C	-0.742131000	6.088749000	2.243523000
H	-1.701400000	6.477072000	1.866009000
H	-0.204917000	6.917215000	2.733508000
H	-0.142110000	5.772587000	1.376501000
C	-1.750321000	5.476862000	4.450089000
H	-1.172872000	6.278582000	4.936343000
H	-2.721464000	5.903109000	4.152828000
H	-1.933278000	4.695175000	5.204043000
C	0.393781000	4.401360000	3.738960000
H	1.017939000	4.034043000	2.910182000
H	0.953779000	5.202601000	4.248473000
H	0.258016000	3.570613000	4.449335000
C	-0.393795000	4.401276000	-3.739014000
H	-1.017964000	4.033934000	-2.910255000
H	-0.953805000	5.202501000	-4.248538000
H	-0.257983000	3.570537000	-4.449389000
C	0.742019000	6.088691000	-2.243531000
H	1.701265000	6.477043000	-1.865988000
H	0.204791000	6.917142000	-2.733525000
H	0.141984000	5.772504000	-1.376528000
C	1.750293000	5.476855000	-4.450072000
H	1.172835000	6.278563000	-4.936333000
H	2.721416000	5.903125000	-4.152778000
H	1.933293000	4.695181000	-5.204029000

1c′-anti: E= -3703.301270

C	-0.704788000	-0.000523000	1.604549000
C	0.687575000	-0.020944000	1.605874000
C	1.421906000	-0.055653000	0.413394000
C	0.688392000	-0.040672000	-0.779853000
C	-0.700380000	0.050296000	-0.780731000
C	-1.436689000	0.049361000	0.411021000
F	-1.335454000	0.108556000	-1.953115000
F	1.326192000	-0.084348000	-1.951381000
F	1.326808000	-0.024974000	2.777290000
F	-1.346296000	-0.012128000	2.774642000
C	-5.044951000	-0.807130000	-0.445483000
C	-3.637528000	-0.944569000	-0.285063000
C	-2.926484000	0.076335000	0.404154000
C	-3.624543000	1.115077000	1.077867000
C	-5.040607000	1.017246000	1.197579000
C	-5.753513000	0.115363000	0.366075000
C	-3.005934000	-2.127638000	-0.890817000
C	-5.747574000	-1.569807000	-1.451909000
C	-7.196703000	0.134337000	0.346374000
C	-5.752049000	1.796676000	2.184908000
C	-2.976208000	2.284968000	1.698189000
C	-3.639208000	-2.778178000	-1.981654000
C	-2.959714000	-3.823051000	-2.650990000
C	-1.716339000	-4.298473000	-2.248815000

C	-1.172999000	-3.736647000	-1.073776000
C	-1.802666000	-2.697706000	-0.412185000
C	-7.151290000	-1.393536000	-1.627081000
C	-7.772242000	-1.982236000	-2.742424000
C	-7.041789000	-2.747510000	-3.646591000
C	-5.685454000	-2.984298000	-3.433274000
C	-5.021469000	-2.431167000	-2.327103000
C	-7.910671000	0.896844000	1.316910000
C	-9.314803000	0.903361000	1.271881000
C	-10.003394000	0.172454000	0.307604000
C	-9.308338000	-0.578124000	-0.636791000
C	-7.903826000	-0.610618000	-0.642975000
C	-5.029369000	2.638726000	3.079825000
C	-5.705438000	3.203436000	4.172424000
C	-7.071956000	2.998722000	4.351609000
C	-7.797918000	2.256438000	3.424986000
C	-7.163935000	1.656337000	2.323069000
C	-1.744898000	2.814276000	1.242647000
C	-1.108802000	3.858034000	1.899912000
C	-1.702292000	4.435023000	3.031163000
C	-2.957208000	4.005327000	3.433049000
C	-3.633194000	2.957961000	2.766977000
C	3.637108000	2.807848000	-1.929577000
C	2.964321000	3.865539000	-2.574312000
C	1.717777000	4.341595000	-2.167514000
C	1.168369000	3.761395000	-1.009875000
C	1.797844000	2.705210000	-0.365807000
C	7.144641000	1.409356000	-1.586226000
C	7.769905000	2.012273000	-2.691804000
C	7.043556000	2.790972000	-3.587405000
C	5.686927000	3.027457000	-3.374014000
C	5.019755000	2.461097000	-2.277164000
C	7.891817000	-0.926328000	1.324722000
C	9.296054000	-0.934135000	1.283870000
C	9.988694000	-0.189154000	0.333365000
C	9.297510000	0.576800000	-0.601435000
C	7.893048000	0.611066000	-0.611497000
C	5.003177000	-2.692118000	3.051316000
C	5.675374000	-3.275103000	4.136742000
C	7.041547000	-3.074990000	4.323175000
C	7.771087000	-2.318826000	3.410668000
C	7.141106000	-1.700538000	2.316594000
C	1.722856000	-2.833386000	1.203744000
C	1.083435000	-3.886252000	1.843211000
C	1.673579000	-4.482104000	2.966412000
C	2.928267000	-4.060871000	3.377686000
C	3.607509000	-3.004279000	2.729694000
C	2.997140000	2.140150000	-0.849087000
C	5.741007000	1.586027000	-1.412231000
C	7.181756000	-0.147792000	0.364128000
C	5.729471000	-1.836883000	2.171998000
C	2.954252000	-2.313519000	1.669978000
C	5.738429000	-0.126848000	0.379862000
C	5.033610000	0.809279000	-0.418923000
C	3.625835000	0.945612000	-0.260872000
C	2.911690000	-0.084951000	0.409536000
C	3.606243000	-1.135342000	1.069352000
C	5.021840000	-1.041163000	1.194944000
H	3.410294000	-4.590035000	4.199608000
H	0.126902000	-4.254252000	1.466645000
H	1.269522000	-2.419967000	0.307080000

H	5.132534000	-3.893684000	4.851109000
H	7.544719000	-3.524601000	5.182567000
H	8.841770000	-2.192100000	3.569964000
H	9.862351000	-1.538129000	1.992494000
H	11.081208000	-0.205476000	0.321101000
H	9.865899000	1.164125000	-1.322257000
H	8.833153000	1.856088000	-2.873117000
H	7.541913000	3.229487000	-4.455328000
H	5.147852000	3.662869000	-4.076406000
H	3.454231000	4.357326000	-3.413464000
H	0.235259000	4.138392000	-0.595550000
H	1.350471000	2.325673000	0.548989000
H	-3.442087000	4.520798000	4.261965000
H	-1.289093000	2.416624000	0.340059000
H	-5.165390000	3.811218000	4.898084000
H	-7.578189000	3.433939000	5.216596000
H	-8.868920000	2.125950000	3.579046000
H	-9.884177000	1.495374000	1.988119000
H	-11.095888000	0.187644000	0.292065000
H	-9.873813000	-1.154856000	-1.368383000
H	-8.835336000	-1.825891000	-2.924537000
H	-7.537156000	-3.175409000	-4.521506000
H	-5.144036000	-3.609572000	-4.142825000
H	-1.361229000	-2.333859000	0.511795000
H	-0.237915000	-4.125651000	-0.669388000
H	-3.449471000	-4.291582000	-3.500170000
H	-0.152678000	4.234005000	1.530297000
H	-1.203638000	5.247533000	3.564969000
H	1.172283000	-5.301937000	3.486355000
C	-0.949282000	-5.402534000	-2.992228000
C	-0.720005000	-6.602060000	-2.044797000
H	-1.679183000	-7.014874000	-1.693996000
H	-0.171789000	-7.403172000	-2.567152000
H	-0.129856000	-6.320524000	-1.159459000
C	-1.706480000	-5.903585000	-4.234033000
H	-1.115650000	-6.680633000	-4.743670000
H	-2.678786000	-6.348176000	-3.969048000
H	-1.883920000	-5.093385000	-4.958543000
C	0.416134000	-4.837424000	-3.447463000
H	1.023437000	-4.498081000	-2.594618000
H	0.992824000	-5.609711000	-3.982583000
H	0.279892000	-3.978709000	-4.123680000
C	1.026023000	5.475483000	-2.943029000
C	-0.343184000	5.835514000	-2.338471000
C	0.806337000	5.026484000	-4.405440000
H	1.756856000	4.793819000	-4.909360000
H	0.175004000	4.124976000	-4.448742000
H	0.307991000	5.823253000	-4.981780000
H	-1.028222000	4.973319000	-2.335399000
H	-0.815366000	6.633349000	-2.932656000
H	-0.252991000	6.203549000	-1.304535000
C	1.917067000	6.737841000	-2.917784000
H	2.092916000	7.073279000	-1.883408000
H	2.898019000	6.556858000	-3.383129000
H	1.433086000	7.561253000	-3.468356000

1c′-anti: E= -3703.301270

C	-0.704788000	-0.000523000	1.604549000
C	0.687575000	-0.020944000	1.605874000
C	1.421906000	-0.055653000	0.413394000

C	0.688392000	-0.040672000	-0.779853000
C	-0.700380000	0.050296000	-0.780731000
C	-1.436689000	0.049361000	0.411021000
F	-1.335454000	0.108556000	-1.953115000
F	1.326192000	-0.084348000	-1.951381000
F	1.326808000	-0.024974000	2.777290000
F	-1.346296000	-0.012128000	2.774642000
C	-5.044951000	-0.807130000	-0.445483000
C	-3.637528000	-0.944569000	-0.285063000
C	-2.926484000	0.076335000	0.404154000
C	-3.624543000	1.115077000	1.077867000
C	-5.040607000	1.017246000	1.197579000
C	-5.753513000	0.115363000	0.366075000
C	-3.005934000	-2.127638000	-0.890817000
C	-5.747574000	-1.569807000	-1.451909000
C	-7.196703000	0.134337000	0.346374000
C	-5.752049000	1.796676000	2.184908000
C	-2.976208000	2.284968000	1.698189000
C	-3.639208000	-2.778178000	-1.981654000
C	-2.959714000	-3.823051000	-2.650990000
C	-1.716339000	-4.298473000	-2.248815000
C	-1.172999000	-3.736647000	-1.073776000
C	-1.802666000	-2.697706000	-0.412185000
C	-7.151290000	-1.393536000	-1.627081000
C	-7.772242000	-1.982236000	-2.742424000
C	-7.041789000	-2.747510000	-3.646591000
C	-5.685454000	-2.984298000	-3.433274000
C	-5.021469000	-2.431167000	-2.327103000
C	-7.910671000	0.896844000	1.316910000
C	-9.314803000	0.903361000	1.271881000
C	-10.003394000	0.172454000	0.307604000
C	-9.308338000	-0.578124000	-0.636791000
C	-7.903826000	-0.610618000	-0.642975000
C	-5.029369000	2.638726000	3.079825000
C	-5.705438000	3.203436000	4.172424000
C	-7.071956000	2.998722000	4.351609000
C	-7.797918000	2.256438000	3.424986000
C	-7.163935000	1.656337000	2.323069000
C	-1.744898000	2.814276000	1.242647000
C	-1.108802000	3.858034000	1.899912000
C	-1.702292000	4.435023000	3.031163000
C	-2.957208000	4.005327000	3.433049000
C	-3.633194000	2.957961000	2.766977000
C	3.637108000	2.807848000	-1.929577000
C	2.964321000	3.865539000	-2.574312000
C	1.717777000	4.341595000	-2.167514000
C	1.168369000	3.761395000	-1.009875000
C	1.797844000	2.705210000	-0.365807000
C	7.144641000	1.409356000	-1.586226000
C	7.769905000	2.012273000	-2.691804000
C	7.043556000	2.790972000	-3.587405000
C	5.686927000	3.027457000	-3.374014000
C	5.019755000	2.461097000	-2.277164000
C	7.891817000	-0.926328000	1.324722000
C	9.296054000	-0.934135000	1.283870000
C	9.988694000	-0.189154000	0.333365000
C	9.297510000	0.576800000	-0.601435000
C	7.893048000	0.611066000	-0.611497000
C	5.003177000	-2.692118000	3.051316000
C	5.675374000	-3.275103000	4.136742000
C	7.041547000	-3.074990000	4.323175000

C	7.771087000	-2.318826000	3.410668000
C	7.141106000	-1.700538000	2.316594000
C	1.722856000	-2.833386000	1.203744000
C	1.083435000	-3.886252000	1.843211000
C	1.673579000	-4.482104000	2.966412000
C	2.928267000	-4.060871000	3.377686000
C	3.607509000	-3.004279000	2.729694000
C	2.997140000	2.140150000	-0.849087000
C	5.741007000	1.586027000	-1.412231000
C	7.181756000	-0.147792000	0.364128000
C	5.729471000	-1.836883000	2.171998000
C	2.954252000	-2.313519000	1.669978000
C	5.738429000	-0.126848000	0.379862000
C	5.033610000	0.809279000	-0.418923000
C	3.625835000	0.945612000	-0.260872000
C	2.911690000	-0.084951000	0.409536000
C	3.606243000	-1.135342000	1.069352000
C	5.021840000	-1.041163000	1.194944000
H	3.410294000	-4.590035000	4.199608000
H	0.126902000	-4.254252000	1.466645000
H	1.269522000	-2.419967000	0.307080000
H	5.132534000	-3.893684000	4.851109000
H	7.544719000	-3.524601000	5.182567000
H	8.841770000	-2.192100000	3.569964000
H	9.862351000	-1.538129000	1.992494000
H	11.081208000	-0.205476000	0.321101000
H	9.865899000	1.164125000	-1.322257000
H	8.833153000	1.856088000	-2.873117000
H	7.541913000	3.229487000	-4.455328000
H	5.147852000	3.662869000	-4.076406000
H	3.454231000	4.357326000	-3.413464000
H	0.235259000	4.138392000	-0.595550000
H	1.350471000	2.325673000	0.548989000
H	-3.442087000	4.520798000	4.261965000
H	-1.289093000	2.416624000	0.340059000
H	-5.165390000	3.811218000	4.898084000
H	-7.578189000	3.433939000	5.216596000
H	-8.868920000	2.125950000	3.579046000
H	-9.884177000	1.495374000	1.988119000
H	-11.095888000	0.187644000	0.292065000
H	-9.873813000	-1.154856000	-1.368383000
H	-8.835336000	-1.825891000	-2.924537000
H	-7.537156000	-3.175409000	-4.521506000
H	-5.144036000	-3.609572000	-4.142825000
H	-1.361229000	-2.333859000	0.511795000
H	-0.237915000	-4.125651000	-0.669388000
H	-3.449471000	-4.291582000	-3.500170000
H	-0.152678000	4.234005000	1.530297000
H	-1.203638000	5.247533000	3.564969000
H	1.172283000	-5.301937000	3.486355000
C	-0.949282000	-5.402534000	-2.992228000
C	-0.720005000	-6.602060000	-2.044797000
H	-1.679183000	-7.014874000	-1.693996000
H	-0.171789000	-7.403172000	-2.567152000
H	-0.129856000	-6.320524000	-1.159459000
C	-1.706480000	-5.903585000	-4.234033000
H	-1.115650000	-6.680633000	-4.743670000
H	-2.678786000	-6.348176000	-3.969048000
H	-1.883920000	-5.093385000	-4.958543000
C	0.416134000	-4.837424000	-3.447463000
H	1.023437000	-4.498081000	-2.594618000

H	0.992824000	-5.609711000	-3.982583000
H	0.279892000	-3.978709000	-4.123680000
C	1.026023000	5.475483000	-2.943029000
C	-0.343184000	5.835514000	-2.338471000
C	0.806337000	5.026484000	-4.405440000
H	1.756856000	4.793819000	-4.909360000
H	0.175004000	4.124976000	-4.448742000
H	0.307991000	5.823253000	-4.981780000
H	-1.028222000	4.973319000	-2.335399000
H	-0.815366000	6.633349000	-2.932656000
H	-0.252991000	6.203549000	-1.304535000
C	1.917067000	6.737841000	-2.917784000
H	2.092916000	7.073279000	-1.883408000
H	2.898019000	6.556858000	-3.383129000
H	1.433086000	7.561253000	-3.468356000

6′: E= -3470.055418

C	0.691532000	0.086516000	2.031556000
C	-0.691373000	-0.086708000	2.031667000
C	-1.427081000	-0.164162000	0.847201000
C	-0.693720000	-0.073933000	-0.342718000
C	0.693512000	0.074239000	-0.342831000
C	1.427064000	0.164194000	0.846974000
F	1.325279000	0.142889000	-1.516109000
F	-1.325679000	-0.142315000	-1.515905000
F	-1.312205000	-0.239736000	3.213182000
F	1.312577000	0.239254000	3.212986000
C	4.650535000	1.488122000	-0.512352000
C	3.437103000	1.542277000	0.213904000
C	2.915987000	0.320060000	0.736502000
C	3.737191000	-0.808480000	0.853201000
C	5.107562000	-0.740366000	0.437789000
C	5.493836000	0.340984000	-0.397524000
C	2.738582000	2.819398000	0.327353000
C	4.993783000	2.565345000	-1.415784000
C	6.714726000	0.265741000	-1.171626000
C	6.113526000	-1.754320000	0.781783000
C	3.143604000	-2.130001000	1.233540000
C	3.053700000	3.880644000	-0.561063000
C	2.350875000	5.101008000	-0.439809000
C	1.401225000	5.323151000	0.551290000
C	1.176424000	4.294906000	1.489361000
C	1.827153000	3.079859000	1.377551000
C	6.135724000	2.446230000	-2.261422000
C	6.375008000	3.447514000	-3.218327000
C	5.522604000	4.539695000	-3.347198000
C	4.423139000	4.672949000	-2.505683000
C	4.146575000	3.711258000	-1.520283000
C	7.583869000	-0.857338000	-1.027342000
C	8.695802000	-0.971018000	-1.878822000
C	8.976626000	0.009655000	-2.823824000
C	8.156009000	1.127327000	-2.938183000
C	7.017945000	1.281089000	-2.128616000
C	5.967554000	-2.616071000	1.897118000
C	6.923894000	-3.563711000	2.223193000
C	8.083546000	-3.682153000	1.442391000
C	8.277830000	-2.816238000	0.378597000
C	7.322451000	-1.830786000	0.034432000
C	3.144253000	-3.167807000	0.286726000

C	2.691834000	-4.444322000	0.626081000
C	2.236279000	-4.705415000	1.921103000
C	2.202314000	-3.672618000	2.862952000
C	2.643466000	-2.392937000	2.519671000
C	-3.053738000	-3.880671000	-0.560894000
C	-2.351163000	-5.101157000	-0.439383000
C	-1.401751000	-5.323414000	0.551921000
C	-1.176959000	-4.295132000	1.489957000
C	-1.827414000	-3.079961000	1.377886000
C	-6.135121000	-2.445820000	-2.262110000
C	-6.374488000	-3.447231000	-3.218849000
C	-5.522299000	-4.539629000	-3.347386000
C	-4.422987000	-4.672961000	-2.505691000
C	-4.146404000	-3.711193000	-1.520356000
C	-7.583292000	0.857736000	-1.028050000
C	-8.694643000	0.971961000	-1.880208000
C	-8.974955000	-0.008246000	-2.825852000
C	-8.154499000	-1.126058000	-2.940057000
C	-7.017065000	-1.280407000	-2.129713000
C	-5.968145000	2.615353000	1.897743000
C	-6.924728000	3.562699000	2.223954000
C	-8.084235000	3.681225000	1.442938000
C	-8.278089000	2.815739000	0.378716000
C	-7.322396000	1.830665000	0.034330000
C	-3.144602000	3.167995000	0.287944000
C	-2.692474000	4.444452000	0.627842000
C	-2.237119000	4.705126000	1.923026000
C	-2.203015000	3.672005000	2.864493000
C	-2.643876000	2.392355000	2.520654000
C	-2.738595000	-2.819399000	0.327495000
C	-4.993427000	-2.565122000	-1.416149000
C	-6.714189000	-0.265383000	-1.172282000
C	-6.113697000	1.754037000	0.782021000
C	-3.143884000	2.129849000	1.234395000
C	-5.493597000	-0.340870000	-0.397747000
C	-4.650274000	-1.487984000	-0.512581000
C	-3.437003000	-1.542245000	0.213916000
C	-2.916007000	-0.320082000	0.736740000
C	-3.737295000	0.808369000	0.853615000
C	-5.107568000	0.740276000	0.437929000
H	-1.839271000	3.863942000	3.877397000
H	-2.708448000	5.241916000	-0.119534000
H	-3.526583000	2.970326000	-0.716095000
H	-6.775405000	4.204006000	3.095935000
H	-8.844853000	4.426847000	1.687360000
H	-9.209940000	2.884576000	-0.181762000
H	-9.352318000	1.837690000	-1.812189000
H	-9.844147000	0.097658000	-3.479518000
H	-8.407871000	-1.881336000	-3.682527000
H	-7.230269000	-3.376513000	-3.888589000
H	-5.719727000	-5.296207000	-4.110657000
H	-3.771625000	-5.538175000	-2.625280000
H	-2.579340000	-5.921648000	-1.120573000
H	-0.493271000	-4.459530000	2.323622000
H	-1.675226000	-2.336562000	2.155789000
H	1.838464000	-3.864876000	3.875760000
H	3.526372000	-2.969865000	-0.717207000
H	6.774254000	-4.205326000	3.094894000
H	8.843932000	-4.428071000	1.686629000
H	9.209767000	-2.885110000	-0.181731000
H	9.353620000	-1.836629000	-1.810721000

H	9.846348000	-0.095779000	-3.476861000
H	8.409804000	1.882999000	-3.680107000
H	7.230846000	3.376817000	-3.888000000
H	5.719964000	5.296155000	-4.110604000
H	3.771617000	5.538017000	-2.625493000
H	1.674975000	2.336468000	2.155462000
H	0.492477000	4.459260000	2.322821000
H	2.578999000	5.921481000	-1.121045000
H	2.707638000	-5.241459000	-0.121654000
C	-0.643476000	-6.623071000	0.639452000
H	-0.550215000	-6.965909000	1.682381000
H	-1.128140000	-7.421454000	0.057688000
H	0.381181000	-6.499731000	0.248405000
H	1.897623000	-5.707982000	2.191987000
H	-1.898737000	5.707671000	2.194289000
C	0.642696000	6.622673000	0.638665000
H	-0.382312000	6.498770000	0.248741000
H	0.550354000	6.966180000	1.681462000
H	1.126589000	7.420797000	0.055911000
H	2.646495000	-1.600083000	3.267095000
H	5.094436000	-2.524781000	2.534728000
H	-5.095179000	2.523925000	2.535552000
H	-2.646819000	1.599167000	3.267729000
